# The Harvard Automated Processing Pipeline for Electroencephalography (HAPPE): Standardized Processing Software for Developmental and High-Artifact Data

**DOI:** 10.3389/fnins.2018.00097

**Published:** 2018-02-27

**Authors:** Laurel J. Gabard-Durnam, Adriana S. Mendez Leal, Carol L. Wilkinson, April R. Levin

**Affiliations:** ^1^Laboratories of Cognitive Neuroscience, Division of Developmental Medicine, Boston Children's Hospital, Harvard Medical School, Harvard University, Boston, MA, United States; ^2^Department of Neurology, Boston Children's Hospital, Boston, MA, United States

**Keywords:** EEG, electroencephalography, automated, pipeline, artifact removal, data quality, EEG processing, development

## Abstract

Electroenchephalography (EEG) recordings collected with developmental populations present particular challenges from a data processing perspective. These EEGs have a high degree of artifact contamination and often short recording lengths. As both sample sizes and EEG channel densities increase, traditional processing approaches like manual data rejection are becoming unsustainable. Moreover, such subjective approaches preclude standardized metrics of data quality, despite the heightened importance of such measures for EEGs with high rates of initial artifact contamination. There is presently a paucity of automated resources for processing these EEG data and no consistent reporting of data quality measures. To address these challenges, we propose the Harvard Automated Processing Pipeline for EEG (HAPPE) as a standardized, automated pipeline compatible with EEG recordings of variable lengths and artifact contamination levels, including high-artifact and short EEG recordings from young children or those with neurodevelopmental disorders. HAPPE processes event-related and resting-state EEG data from raw files through a series of filtering, artifact rejection, and re-referencing steps to processed EEG suitable for time-frequency-domain analyses. HAPPE also includes a post-processing report of data quality metrics to facilitate the evaluation and reporting of data quality in a standardized manner. Here, we describe each processing step in HAPPE, perform an example analysis with EEG files we have made freely available, and show that HAPPE outperforms seven alternative, widely-used processing approaches. HAPPE removes more artifact than all alternative approaches while simultaneously preserving greater or equivalent amounts of EEG signal in almost all instances. We also provide distributions of HAPPE's data quality metrics in an 867 file dataset as a reference distribution and in support of HAPPE's performance across EEG data with variable artifact contamination and recording lengths. HAPPE software is freely available under the terms of the GNU General Public License at https://github.com/lcnhappe/happe.

## Introduction

Electroencephalography (EEG) is a sensitive means to noninvasively capture neurophysiological activity with clinical and basic science utility across a number of fields. However, during acquisition, the EEG signal is contaminated by both experimental artifacts like electrical interference and electrode displacement, and participant-induced artifacts like eye and muscle movements. These artifact signals are in many cases far more prominent than the neurophysiological signal, significantly distorting EEG as a measure of brain function if left uncorrected (Cuevas et al., 2014; Keil et al., 2014). Therefore, a series of post-acquisition processing transformations are typically applied to the EEG signal to address these artifacts and prepare the data for analyses, including filtering, artifact removal, and signal re-referencing (Keil et al., 2014). However, the pipelines and parameters for EEG processing vary across studies with little standardization. For example, one common artifact removal approach is labor and training-intensive researcher selection of uncontaminated EEG data, the criteria for which is partially subjective and therefore inconsistent across individuals. Standardized, automatable EEG processing pipelines thus offer several advantages, including uniform application of artifact removal criteria, efficient workflow with large sample sizes, and the facilitation of data comparisons across studies, labs and sites in multi-institution projects. Accordingly, software tools automating various stages of EEG processing have become a methodological focus (e.g., PREP, Bigdely-Shamlo et al., 2015, FASTER, Nolan et al., 2010, ADJUST, Mognon et al., 2011, TAPEEG, Hatz et al., 2015, ASR, Mullen et al., 2013, MARA, Winkler et al., 2014, SASICA, Chaumon et al., 2015), but they have largely been developed and tested on healthy adult EEG data with low levels of artifact contamination (Nolan et al., 2010; Mognon et al., 2011; Mullen et al., 2013; Bigdely-Shamlo et al., 2015; Chaumon et al., 2015; Hatz et al., 2015).

Notably, EEG data from developmental populations like infants, young children, and people with neurodevelopmental disorders present further challenges to extracting uncontaminated signal. EEG signals from these populations have the highest levels of artifact contamination (e.g., infants cannot follow instructions to refrain from moving their mouth or eyes during data collection), and protocols typically have far shorter EEG collection times than those with healthy adults to accommodate reduced tolerance for testing (Tran et al., 2004; Cuevas et al., 2014). Moreover, additional polygraphic measurements used to identify physiological artifacts, like EOG electrodes, are not typically used during EEG acquisition with developmental populations due to both reduced tolerance of their acquisition and decreased signal quality. These factors combined have made it difficult to directly apply contemporary processing approaches from the adult EEG literature, like independent component analysis (ICA), that require longer recordings to most effectively parse artifact from signal (Makeig et al., 1996; Delorme and Makeig, 2004; Albera et al., 2012; Grandchamp et al., 2012) (although see Zima et al., 2012; Piazza et al., 2016). However, the typical manual artifact rejection approaches used for these developmental EEG data routinely remove the majority of the EEG recording, reducing experimental power and sacrificing the neurophysiologically relevant aspects of EEG also contained within the rejected segments (Tran et al., 2004; Tierney et al., 2012; Cuevas et al., 2014; Gabard-Durnam et al., 2015). Moreover, the emerging focus on collecting larger datasets through repositories and large-scale studies along with the use of higher-density EEG nets make manual data selection increasingly impractical as a processing strategy (Bigdely-Shamlo et al., 2015). Currently, there is an unmet and growing need for automated processing tools suitable for EEG recordings like those generated by these populations.

The purpose of the Harvard Automated Preprocessing Pipeline for EEG (HAPPE) is to provide an automated, standardized approach for processing these classes of EEG data. Specifically, HAPPE is designed for data with high levels of artifact or very short recording length (on the scale of several minutes), although the pipeline may appropriately be used with longer or less-contaminated data. HAPPE integrates Matlab-based (The Mathworks, Inc.) code with freely available academic software, including EEGLAB functions (Delorme and Makeig, 2004), to automatedly batch process resting-state and event-related EEG data from raw format to corrected signal prepared for analyses in the frequency domain. HAPPE comprises both a fully-automated and semi-automated setting so that users may visualize processing performance on individual EEG files at multiple stages in the semi-automated setting, and adjust user inputs if desired, before running the complete dataset through the fully-automated pipeline. The following sections detail each of the proposed HAPPE pipeline's processing steps and post-processing report metrics, demonstrate HAPPE's effectiveness under variable conditions of EEG artifact and recording length by including an analysis of 10 developmental files, and compare HAPPE's performance to that of seven alternative, widely-used processing approaches.

## The harvard automated preprocessing pipeline for EEG (HAPPE)

### HAPPE EEG inputs

**Figure 1:**
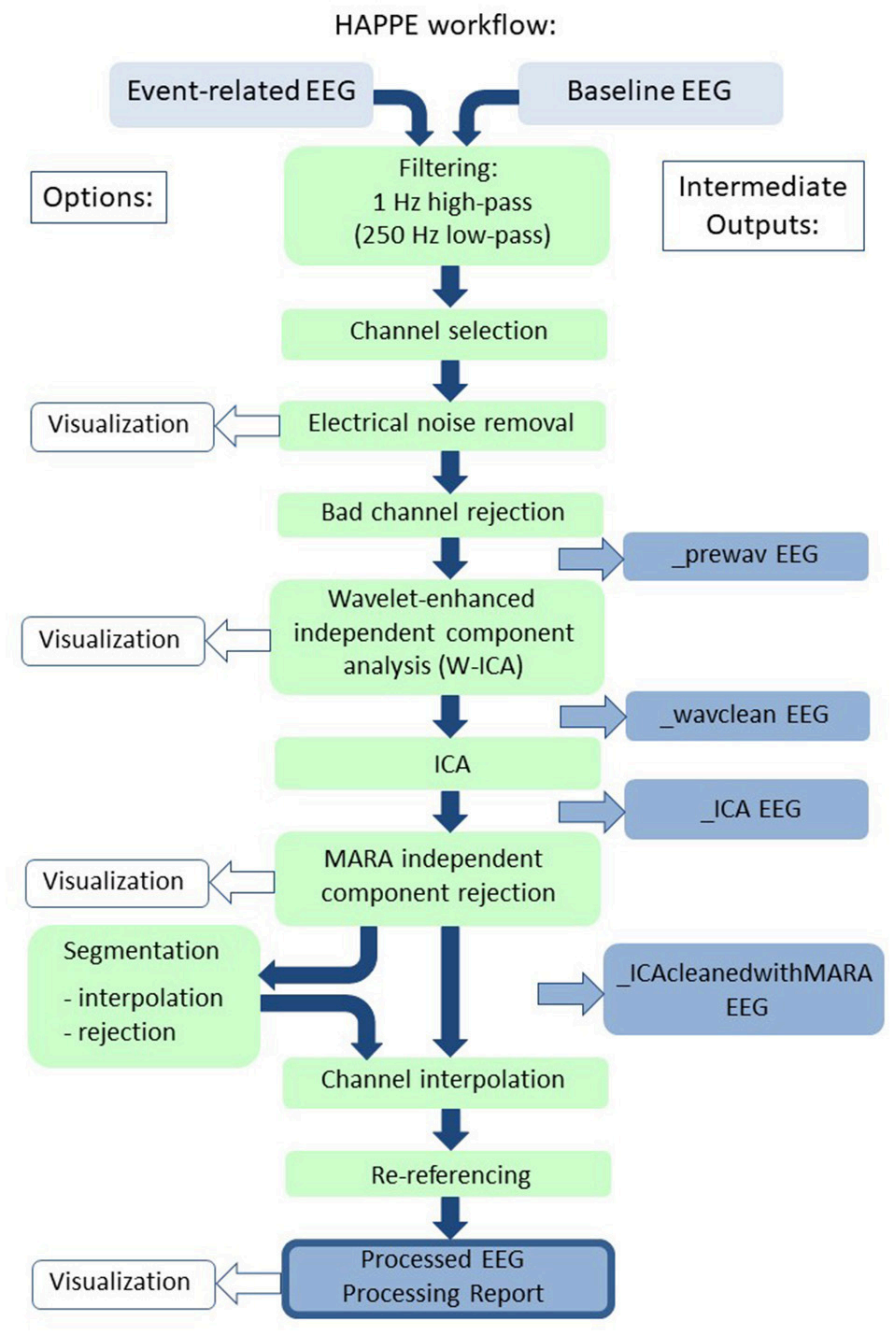
Schematic illustrating the HAPPE pipeline's processing steps. The intermediate output EEG files are indicated by the suffix added after that specific processing step in the blue boxes. The user options for segmentation steps and visualizing several steps in HAPPE with the semi-automated setting are also indicated. Independent component analysis is abbreviated to ICA.

HAPPE accommodates multiple types of EEG with different acquisition parameters as inputs. HAPPE reads data in from EGI-exported (Electrical Geodesics, Inc.) Matlab files for resting-state EEG, and the data may have differing identifying variable names across files. HAPPE reads EGI-exported simple binary files for event-related EEG presently. However, users can easily modify the importing code to read any file format for any resting-state or event-related EEG that EEGLAB accepts. An individual HAPPE run should include only resting-state data or only event-related data, and users must specify one or the other as the input file type. HAPPE is currently compatible with EEG layouts of 64 and 128 channels. Each run of HAPPE must include files collected with the same channel layout (company and electrode number) and users must specify the appropriate channel layout in a given HAPPE run. Users who wish to include different EEG channel layouts within a single pipeline run can easily do so by accessing HAPPE through the Batch EEG Automated Processing Platform (BEAPP) software, available at https://github.com/lcnbeapp/beapp. HAPPE processes data collected with any sampling rate, and files within a single run of HAPPE may differ in their individual sampling rates. A schematic of HAPPE's processing steps, options, and outputs is provided (Figure 1).

HAPPE consists of the following processing steps for EEG data:

1. Filtering
2. EEG channel subset selection
3. Electrical (line) noise removal
4. Bad channel rejection
5. Wavelet-enhanced thresholding (W-ICA)
6. ICA with automated component rejection
7. Automated segment rejection (optional)
  - Segmentation (optional)
  - Interpolation of bad channels for each epoch (optional)
8. Interpolation of bad channels
9. Re-referencing

Below, the implementation of each processing step in HAPPE is described in detail.

### Filtering

All files are subject to a 1 Hz high-pass filter. The filter removes non-stationary signal drift across the recording and serves as a pre-processing step for the electrical noise removal and ICA steps that follow (Bigdely-Shamlo et al., 2015; Winkler et al., 2015). ICA has been shown to perform best at separating signals following a 1–2 Hz high-pass filter on the data (Winkler et al., 2015). For files collected with sampling rates equal to or greater than 500 Hz, the 1Hz filter is incorporated into a band-pass filter from 1 to 249 Hz to constrain the signal decomposed by ICA.

### Selection of EEG channel subset

Users must specify a subset of 19 channels corresponding to the International 10–20 system electrodes (without the Cz reference electrode) for their channel layout (for use later with automated ICA artifact rejection) and any additional channels they wish to be processed in HAPPE. Channels that are not provided in the subset list are removed from subsequent processing and cannot be recovered later. For example, for data from a 128-channel net where the user selects 50 channels, the post-HAPPE processed data will contain only data for those 50 selected channels. Channel subset selection facilitates the use of ICA within the context of short EEG recordings where robust ICA decomposition is unlikely if all of the high-density channels are inputs. Typically, ICA decomposes signal into the same number of signal sources as net channels (assuming no channel interpolation has occurred yet) (Makeig et al., 1996). To generate a robust, stable ICA decomposition and avoid overlearning in the data, there are recommended constraints on how many channels may be decomposed given the length and sampling rate of an EEG recording (Särelä et al., 2003). Specifically, it is presently recommended that an EEG recording have at least 30 ^*^ (the number of channels)^2^ data samples to undergo ICA decomposition (e.g., Onton and Makeig, 2006). For example, an EEG acquired with a 128-channel net and sampling rate of 500 Hz (500 samples/second) would need at least 491,520 samples (30 ^*^ 128^2^ samples), that is, 983.04 s of recording (491,520 samples/500 Hz) to be reliably decomposed with ICA.

However, short EEG recordings, like those usually captured with developmental populations, and especially short EEG recordings made with high-density channel layouts (e.g., 128, 256 channels), do not provide enough data samples for reliable ICA decomposition without data dimension reduction. Therefore, HAPPE implements channel subset selection to improve the robustness of the ICA decomposition for these types of data. The number of channels that can be processed in a single HAPPE run will therefore depend on how long a user's EEG recordings are and the sampling rate during acquisition. For example, selecting 40 out of 128 channels to process abaseline EEG recording with a 250 Hz sampling rate would require 48,000 samples for adequate ICA (30 ^*^ 40^2^ = 48,000). A 5-min recording with 75,000 samples would easily provide adequate data samples. The 19 channels from the 10–20 electrodes must be included in the total number of electrodes users select (i.e., in this example, the user may select an additional 21 electrodes from their channel layout to include with the 10–20 electrodes in their channel subset). It should be noted that if the length and sampling rate of the EEG files in a study permit ICA decomposition on the entire number of channels, then the full set of channels may be entered in the user input as the channel “subset.”

### Electrical (line) noise removal

HAPPE removes electrical noise (e.g., 60 or 50 Hz artifact signal) from the EEG through the multi-taper regression approach implemented by the CleanLine program (Mullen, 2012). Multi-taper regression can remove electrical noise without sacrificing or distorting the underlying EEG signal in the nearby frequencies, drawbacks of the notch-filtering approach to line-noise removal (Mitra and Pesaran, 1999). Specifically, HAPPE applies CleanLine's multi-taper regression with enabled scanning for exact line-noise frequency near the user-specified frequency ± 2 Hz, a 4-s window with a 1-s step size and a smoothing tau of 100 during the fast Fourier transform, and a significance threshold of *p* = 0.01 for sinusoid regression coefficients during electrical noise removal. Any remaining line-noise in the data after CleanLine regression is further addressed through the wavelet-thresholding, ICA, and re-referencing (if average re-reference is selected) steps that follow.

### Bad channel rejection

HAPPE identifies and removes channels, including channels with high impedances or displacement during recording. HAPPE labels such channels as “bad channels,” and their data are not included in further processing or analyses. HAPPE determines bad channels by evaluating the normed joint probability of the average log power from 1 to 125 Hz across the user-specified subset of included channels. Channels whose probability falls more than 3 standard deviations from the mean are removed as bad channels. The bad channel evaluation is performed twice per data file, as channels that would otherwise be manually identified as bad channels (e.g., no signal, displacement visually evident) were found to remain in the data after the initial joint probability evaluation, but were successfully identified during the second evaluation during HAPPE development. Channels removed as bad channels have their data interpolated from nearby channels in a later processing step (after ICA decompositions) to preserve the complete user-selected channel set for post-processing analyses.

### Wavelet-thresholding (W-ICA)

For studies with lower levels of EEG artifact, data segments with obvious artifact contamination, especially non-stereotyped artifact (e.g., signal discontinuity), are commonly rejected before performing ICA as an artifact rejection approach (Grin-Yatsenko et al., 2010; Piazza et al., 2016). The initial data rejection step improves subsequent ICA segregation into artifact and neural components. However, in developmental EEG data files, the high degree of artifact coupled with often brief recording times would lead to inefficient data sacrifice through this segment rejection approach (Cuevas et al., 2014). Therefore, HAPPE implements a wavelet-enhanced ICA (W-ICA) approach described below in detail as a preliminary step to correct for EEG artifact while retaining the entire length of the data file, before performing ICA to reject artifact components. This approach of W-ICA followed by ICA is supported by prior work showing that using wavelet-thresholding approaches before ICA improves the resulting ICA decomposition of the EEG data (Rong-Yi and Zhong, 2005). The W-ICA step removes multiple classes of artifact, including eye and muscle-generated artifacts, high-amplitude artifacts (e.g., blinks), and signal discontinuities (e.g., electrodes losing contact with the scalp).

W-ICA entails first performing an ICA decomposition of the EEG signal into components, after which all of the components' timeseries are subjected to wavelet transform and thresholded to remove artifact before all of the components' timeseries are translated back to EEG channel format (Castellanos and Makarov, 2006). That is, all ICA components are subjected to the wavelet thresholding to remove artifact within each component, but no ICA components are entirely rejected at this stage in the pipeline. Although the initial ICA insufficiently segregates the data into neural and artifact components for optimal ICA component rejection at this stage, artifact is more clustered into specific components compared to the raw channel-wise data. Wavelet-thresholding the ICA-derived components, instead of the raw data, increases the contrast between artifact and neural signal magnitudes to circumvent tuning threshold parameters while also improving W-ICA performance (Castellanos and Makarov, 2006). HAPPE performs the ICA step of W-ICA using the extended Infomax algorithm to increase sensitivity to any remaining electrical noise and other sources with subgaussian or supergaussian activity distributions (Jung et al., 1998). Relative to other ICA algorithms and decomposition methods, the extended Infomax algorithm has been shown to be an optimal approach for decomposing electrophysiological signals like EEG (Delorme et al., 2007).

The wavelet-thresholding step of W-ICA first subjects the component time series to wavelet transform, which produces a series of coefficients to describe the EEG signal. Here, stationary wavelet transform of the complete set of independent components is carried out using a Coiflets (level 5) wavelet. The Coiflets wavelet family was selected because it has been found to provide optimal extraction of neural from artifact signal across both typical and epileptic EEG recordings (Gandhi et al., 2011). HAPPE decomposes data into detail coefficients for frequencies below approximately 125 Hz and above approximately 8 Hz (i.e., the frequency resolution of the wavelet transform; although HAPPE was not tested on EEG data with pathological waveforms, like epileptic EEG, HAPPE should preserve the low-frequency abnormalities like spike-and-slow wave complexes observed in these populations. Further testing on epileptic or other abnormal EEG data should be performed in the future to confirm HAPPE as a robust processing strategy for these cases). The coefficients are then subjected to thresholding, such that coefficients with values smaller than the threshold have their contribution to the data substantially suppressed (similar to Rong-Yi and Zhong, 2005; Jansen, 2012). Thresholding in HAPPE is performed using the Matlab function ddencmp. The global, soft threshold is determined automatically for each file using the signal's variance and length, following the formula:

$$Threshold=\frac{median\left[abs\left(D\right)\right]}{0.6745}*\sqrt{2\log\left(N\right)}$$

where *D* is the set of detailed coefficients provided by the wavelet transform, and *N* is the length of the ICA components

This formula is a scaled version of the universal threshold first proposed by Donoho and Johnstone (1994) that also incorporates a robust estimate of the signal variance. Soft-thresholding (Donoho, 1995) has been implemented in prior studies of wavelet-thresholding electrophysiological data for artifact rejection (e.g., Al-Qazzaz et al., 2017). As in prior W-ICA studies, given that the magnitude of artifacts can be far greater than that of neurophysiological signals, the component time series whose amplitudes are large enough to survive the wavelet-thresholding are taken as the artifact timeseries (similar to Castellanos and Makarov, 2006). These artifact time series are then subtracted from the pre-thresholded timeseries to remove those artifacts from the EEG data.

### ICA with automated component rejection

After W-ICA removes some of the most severe artifacts, the EEG data is more suitable for ICA decomposition with automated component rejection to address the remaining artifacts. HAPPE implements the ICA extended-Infomax algorithm as before. Automated component rejection is achieved through the Multiple Artifact Rejection Algorithm (MARA), a machine-learning algorithm that evaluates the ICA-derived components (Winkler et al., 2011, 2014). Although other algorithms exist to detect specific categories of artifact automatically (e.g., eye-movement artifact and signal discontinuities, Mognon et al., 2011), MARA has been trained on manual component classifications, and so captures the wide range of artifact that manual rejection detects. MARA has proven especially effective at detecting and removing muscle artifact components (see approach comparisons in section HAPPE Compared to Other Common Processing Approaches below). Specifically, MARA evaluates each component on the 6 algorithm features described below and then assigns the component a probability that it is dominated by artifact signal. This probability may be interpreted as the percent of artifact contamination estimated to be in the component. As in the original applications of MARA, HAPPE automatically rejects any components with artifact probabilities greater than 0.5 (i.e., more than 50% likely to be an artifact component) (Winkler et al., 2011, 2014). Statistics for all retained components' artifact probabilities are used to generate data quality metrics detailed in section Median_Artifact_Probability_of_Kept_ICs Mean_Artifact_Probability_of_Kept_ICs Range_Artifact_ Probability_of_Kept_ICs Min_Artifact_Probability_of_Kept_ICs Max_Artifact_Probability_of_Kept_ICs below.

MARA uses 6 data features based on temporal, spectral, and spatial information to assign artifact probability to an independent component, as briefly described below (and detailed in Winkler et al., 2014).

1. Mean local skewness: The first feature is mean local skewness in the data (a temporal feature calculated over 15 s increments). The local skewness feature identifies components with time series outliers, where higher skewness values indicate likely artifact (e.g., a component capturing blinks, or an electrode losing contact with the scalp).

The next three features all rely on information from the frequency domain.

2. Log alpha power: The second feature is the average log power in the alpha band (defined as 8–13 Hz). Brain-derived components typically manifest robust levels of alpha-band power, whereas artifact-driven components do not (this feature does not flag any specific artifact types, but instead reflects a shared, general feature of artifact components). The application of this feature to developmental data is discussed in detail below.

3. Lambda: The third feature, lambda, captures the degree to which a component's power spectrum deviates from the prototypical 1/f distribution observed in cerebral-derived components. The power for six frequencies across the power spectrum are sampled for each component to generate its spectrum power curve and calculate lambda. This feature is particularly sensitive to muscle artifact, which typically manifests as a power spectrum with very poor fit to the 1/f distribution (including sharply increased power in the higher frequencies like beta and gamma after an initial decrease in power through the lower frequencies).

4. Fit error: Similarly, the fourth feature, fit error, represents the mean squared error of the approximation of the f distribution to each component's distribution specifically in the 8–15 Hz range that captures alpha band power and the transition to beta band power. The fit error feature is nonspecific to artifact types but instead serves as a generalized marker of artifact probability.

The last two features make use of spatial information to detect artifactual components.

5. Range within pattern: Specifically, the fifth feature, range within pattern, takes the (log) difference between the largest and smallest activation magnitudes across the scalp for a component, where artifact components typically exhibit larger range within patterns (e.g., eye artifacts and muscle movement result in concentrated areas of very high magnitude relative to the other electrodes' magnitude, whereas cerebral-derived components tend to have more consistent magnitudes across electrodes, and thus smaller magnitude ranges).

6. Current density norm: The final feature, current density norm, makes use of the 10–20 channel locations input to MARA and reflects the solution to source-modeling the component using a model that was designed to fit cerebral-based activity (the minimum current density norm value reflects the simplest source model that is most-likely to be cerebral activity). Since external artifacts were not meant to be modeled with this approach, artifact-driven components return very high current density norms (reflecting overly complex source models). The current density norm feature is similarly non-specific to certain types of artifact components, but instead captures general artifact probability.

Together, these 6 features address a comprehensive range of artifacts observed in independent components.

MARA was not trained specifically on developmental or patient data, but several findings support its application in these contexts. First, the anatomical correlations of the 10–20 electrodes that MARA uses to calculate its spatial features are highly consistent across infant and adult brains (Kabdebon et al., 2014). That is, comparable information is supplied to MARA's spatial features from the 10–20 electrodes regardless of age. Second, a potential concern during HAPPE development was that one of MARA's spectral features evaluates EEG alpha band power, where very young infants or clinical populations may show a different frequency power peak than the alpha band observed in healthy adults (Stroganova et al., 1999; Lansbergen et al., 2011). However, empirically, even the youngest infants tested (3 months of age) in the present dataset had enough alpha power in the components to make use of this MARA criterion appropriately. Indeed, the 3-month files in the example analysis below had the lowest rates of MARA component rejection in the sample. Variation in the alpha band peaks was also preserved across the developmental datasets and was consistent within individual files before and after MARA component rejection, suggesting alpha peaks were unperturbed during the component rejection algorithm. Lastly, the rates of MARA component rejection for the datasets run through HAPPE were comparable to both the rejection rates for the adult data used to validate MARA (Winkler et al., 2011, 2014) and to rates of manual component rejection with developmental data decomposed with ICA (Piazza et al., 2016). HAPPE therefore includes MARA as a robust evaluation tool for component rejection suitable for developmental and clinical data.

### Segmentation (optional)

HAPPE includes an optional data segmentation step along with several additional artifact rejection steps to further optimize processing. For data with event markers (e.g., event-related EEG data), data can be segmented around events as specified by user inputs. For data without event markers (e.g., resting-state EEG), regularly marked segments of any duration specified by the user are generated from the start of the EEG file for the duration of the recording.

After segmentation, several additional artifact-reduction options are available, although users may also segment their data without applying the following options. Post-segmentation artifact reduction parameters that the user chooses may depend on the number of available segments, as well as the extent of artifact contamination remaining in individual segments after prior preprocessing steps.

Users with relatively short data files, for whom segment rejection would lead to an unacceptably low remaining number of segments for analysis, may choose an optional post-segmentation step involving the interpolation of data for channels determined to be artifact-contaminated within each individual segment, as implemented by FASTER software (Nolan et al., 2010). Each channel in each segment is evaluated on the four FASTER criteria (variance, median gradient, amplitude range, and deviation from mean amplitude), and the Z score (a measure of standard deviation from the mean) for each channel in that segment is generated for each of the four metrics. Any channels with one or more Z scores that are greater than 3 standard deviations from the mean for an individual segment are marked bad for that segment. These criteria may identify segments with residual high-amplitude artifacts (e.g., eye artifacts), electrode discontinuity (e.g., electrode has lost contact with the scalp temporarily), and muscle artifact. Subsequently, for each segment, the bad channels have their data interpolated with spherical splines, as in FASTER. This allows users to maintain the maximum number of available segments, while still maximizing artifact rejection within individual segments.

Alternatively, for users with relatively long data files (for whom some segment rejection is less of a concern), or for users who wish to avoid interpolating data within individual segments, the second optional step is segment rejection based on both amplitude and joint probability criteria. Amplitude-based rejection is useful for high-amplitude artifacts like eye blinks, while joint probability-based rejection catches other classes of artifacts, especially high-frequency artifacts like muscle artifact. Together, these two criteria are an effective and time-efficient combination for determining artifact-contaminated segments. This combination has previously been used with developmental EEG data (Delorme et al., 2007; Piazza et al., 2016). Users specify an artifact amplitude threshold for the amplitude-based rejection step, such that any segment with at least one channel whose amplitude crosses the threshold will be marked for rejection. The HAPPE default for the artifact threshold is 40 microvolts, reflecting the smaller overall signal amplitude that results from the wavelet-thresholding and ICA steps. However, users are encouraged to run the semi-automated HAPPE setting on at least several files to visually check that this default amplitude results in appropriate segment rejection in their own datasets. Next, two joint probabilities are calculated with EEGLAB's pop_jointprob function. The joint probability of an electrode's activity in a segment given that same electrode's activity in all other segments is calculated (single electrode probability), and the joint probability of an electrode's activity in a segment given all other electrodes' activities for that same segment is calculated (electrode group probability). These joint probabilities are evaluated such that any segment is marked for rejection when either (1) a channel's single electrode probability or (2) its electrode group probability is outside of 3 standard deviations from the mean are marked for rejection. This criterion most successfully identified segments with remaining high-frequency artifact during HAPPE development. All segments marked from either the amplitude or joint probability criteria are then rejected simultaneously in a single step.

Notably, this segment rejection step may be runon all user-specified channels, or on asubset of channels for a specific region of interest (ROI). The ROI-channel subset option allows users to tailor segment rejection for a specific ROI analysis if multiple ROIs were included in the channels selected for HAPPE processing.

### Interpolation of bad channels

For all HAPPE runs (regardless of segmentation options), any channels removed during the bad channel rejection processing step are now subject to spherical interpolation (with Legendre polynomials up to the 7th order) of their signal. Channel interpolation repopulates data for the complete channel subset specified by the user and reduces bias in re-referencing if the average re-reference option is selected. The identity of all interpolated channels, if any, for a file are recorded in HAPPE's processing report for users who wish to monitor the percentage or identity of interpolated channels in their datasets before further analysis.

### Rereferencing

HAPPE's final processing step is to re-reference the EEG data. The user may specify either re-referencing using an average across all channels (i.e., average re-reference) or using a channel subset of one or multiple channels. For both re-referencing options, only channels within the user-specified channel set selected for HAPPE processing can be used for re-referencing. Rereferencing also reduces artifact signals that exist consistently across electrodes, including residual line-noise.

### HAPPE EEG outputs

HAPPE generates several folders containing EEG files that are located within the user-specified folder of files for processing. EEG files are saved out after several intermediate processing steps so that users can explore in-depth and visualize how those steps affected the EEG signal in their own datasets. The intermediate files include minimally-processed EEG data, post-wavelet-thresholded data, data post-ICA with the component information intact, post-component rejection EEG data, and if segmentation parameters are selected, files with post-segmentation EEG data. HAPPE outputs fully-processed files that are suitable inputs for further analyses (e.g., time-frequency decomposition) in one of several formats to increase compatibility with other software for data visualizations or statistical analyses. HAPPE also outputs the HAPPE processing report (described below) for the file batch, and, if users ran HAPPE in the semi-automated setting, an image for each EEG file containing the fully-processed EEG's power spectrum.

### HAPPE processing report

For each run, HAPPE generates a report table of descriptive statistics and data metrics for each EEG file in the batch in a single spreadsheet to aid in quickly and effectively evaluating pipeline performance and data quality across participants within or across studies (see example in Table 1). The report table with all of these metrics is provided as a .csv file in the “processed” folder generated during HAPPE.

**Table 1:** Example HAPPE processing report for the 10 files in the example dataset.

**Filename**	**File_ length_ in_sec**	**Number_ channels_ userSelected**	**Number_ epochs_ post_ epoch _ rejection**	**Number_ good _ channels_ selected**	**Percent_ good _ channels_ selected**	**Interpolated_ channel_ IDs**	**Number_ Ics_ rejected**	**Percent_ ICs_ rejected**	**Percent_ variance_ kept_of_ post_ waveleted_ data**	**Median_ artifact_ probibility_ of_ kept_ ICs**	**Mean_ artifact_ probibility_ of_ kept_ ICs**	**Range_ artifact_ probibility_ of_kept_ ICs**	**Min_ artifact_ probibility_ of_kept_ ICs**	**Max_artifact_ probibility_ of_ kept_ ICs**
baselinEEG01	348.996	39	72	38		C4	19	50	48.28	0.09529	0.13629	0.49321	0.00582	0.49903
baselineEEG04	188.996	39	80	36	92.31	FP1 FP2 T4	14	38.89	80.39	0.16189	0.15533	0.47391	0.00030	0.47421
baselineEEG05	163.996	39	34	35	89.74	E112 E117 FP2 PZ	13	37.14	79.08	0.01309	0.08033	0.46015	0.00025	0.46040
baselineEEG06	783.996	39	358	34	87.18	E112 E13 FP2 T4 T6	1	2.94	98.07	0.02761	0.05101	0.26725	0.00034	0.26758
baselineEEG07	144.996	39	60	36	92.31	F8 FP1 T5	1	2.78	99.13	0.04811	0.04776	0.12606	0.00112	0.12717
baselineEEG08	278.998	39	131	33	84.62	E13 E4 F7 FP1 PZ T3	25	75.76	43.67	0.24807	0.20781	0.41921	0.00351	0.42272
baselineEEG09	160.998	39	65	35	89.74	E112 E75 O1 O2	25	71.43	24.25	0.08369	0.15050	0.45486	0.00381	0.45867
baselineEEG10	377.998	39	43	32	82.05	E112 E75 F7 O1 O2 PZ T6	14	43.75	83.82	0.11978	0.15529	0.46351	0.00072	0.46423
baselineEEG11	183.996	39	55	31	79.49	C4 E112 E118 E123 FP1 FP2 PZ T6	11	35.48	85.15	0.02869	0.07420	0.42093	0.00248	0.42341
baselineEEG12	283.996	39	91	35	89.74	E102 E112 E118 E98	22	62.86	35.67	0.17657	0.22590	0.41931	0.01900	0.43831

The data metrics are each briefly described below. In addition, to inform understanding about the distribution of values for each metric that may be expected in a developmental population, we provide descriptive distributions from a sample of 867 developmental EEGs. This dataset includes EEGs from participants spanning 3 months to 36 months of age, and contains three participant groups as part of a larger, longitudinal study on the emergence of Autism Spectrum Disorder (ASD) (see Tierney et al., 2012) for detailed description of the project). Typically developing infants (“Low Risk/No Autism” group), infants at high risk for ASD by virtue of having an older sibling with an ASD, but who did not go on to receive an ASD diagnosis themselves (“High Risk/no Autism” group), and infants at high risk for ASD who did go on to receive an ASD diagnosis (“High Risk/Autism” group) contribute data to this sample. The project was approved by the Institutional Review Board (the local ethics committee) at Boston University and Boston Children's Hospital (#X06-08-0374), and was carried out with written informed consent from all caregivers prior to their child's participation in the study. For each metric, the distribution of values across this large sample is presented grouped by age, and separately, by clinical risk status. The descriptive statistics for the entire 867 file sample for all metrics are also provided in Table 2. These distributions may aid users in setting thresholds for removing files from further analysis due to poor data quality, and in comparing HAPPE performance in their own data to HAPPE performance with the present sample.

**Table 2:** Descriptive statistics for data quality metrics in a developmental sample.

	**File length**	**% Good channels**	**% ICs rejected**	**% Variance kept**	**Mean artifact probability**	**Median artifact probability**
Mean	208.27	91.65	40.57	67.14	0.14	0.1
Std	125.45	5.29	12.4	16.75	0.05	0.07
25%	134	89.7	32.14	56.84	0.11	0.05
50%	166	92.3	40.54	68.98	0.14	0.09
75%	264	94.87	48.65	79.09	0.18	0.14

*Sample size for the dataset is 867 EEG files. For each metric, the mean value (mean), standard deviation (std), and values corresponding to the 25th, 50th, and 75th percentile are given*.

#### Number_epochs_post_epoch_rejection

First, if the user selected the segment rejection option in HAPPE, they may evaluate the number of data segments remaining post-rejection for each file to identify any files that cannot contribute enough clean data to be included in further analyses (user discretion). The user may also easily tabulate the descriptive statistics for remaining segments to report in their manuscript's Methods section (e.g., the mean and standard deviation of the number of usable data segments per file in their study).

#### Percent_good_channels_selected Interpolated_channel_IDs

**Figure 2:**
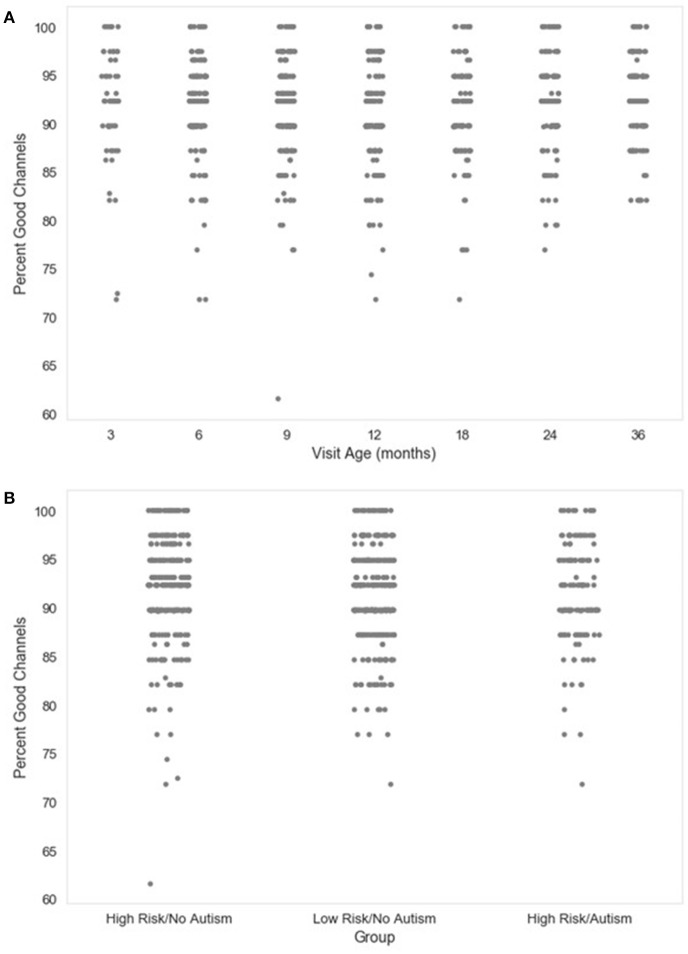
Percent good channels retained. The distribution of the percent of channels retained as good channels during channel rejection is shown as a function of age when the EEG was acquired **(A)**, or clinical group status **(B)** for a developmental sample.

Next, the percentage of channels contributing uninterpolated data (“good channels”) and the identity of interpolated channels are provided. Users wishing to limit the amount of interpolated data in further analyses can easily identify files for removal using these two metrics. The distributions of the percent of good channels for the developmental sample included here are visualized in Figure 2.

#### Number_ICs_rejected Percent_ICs_rejected

**Figure 3:**
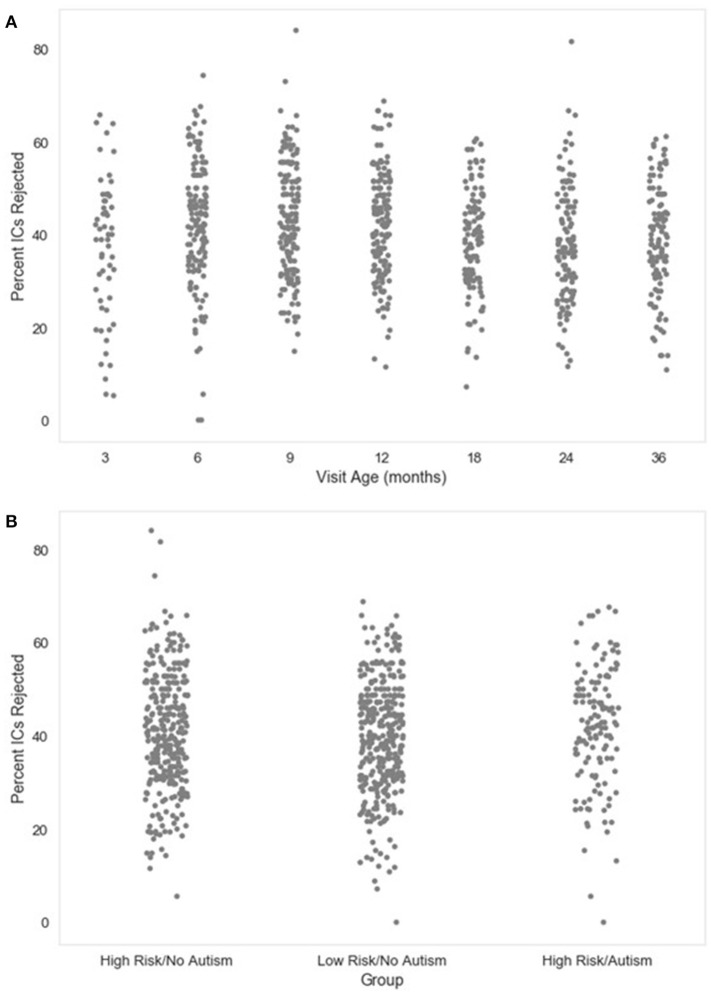
Percent of independent components (ICs) rejected. The distribution of the percent of independent components rejected by MARA after ICA decomposition is shown as a function of age when the EEG was acquired **(A)**, or clinical group status **(B)** for a developmental sample.

The number and percent of the independent components rejected as artifact components by MARA after the post-wavelet-thresholded ICA decomposition are also given. These measures may be useful for evaluating how ICA with MARA performs on the user's data across files, since poor artifact segregation from neural signal would result in MARA rejecting most components consistently across files. The distributions of the percent of components rejected in the developmental sample included here are visualized in Figure 3.

#### Percent_variance_kept_of_post_waveleted_data

**Figure 4:**
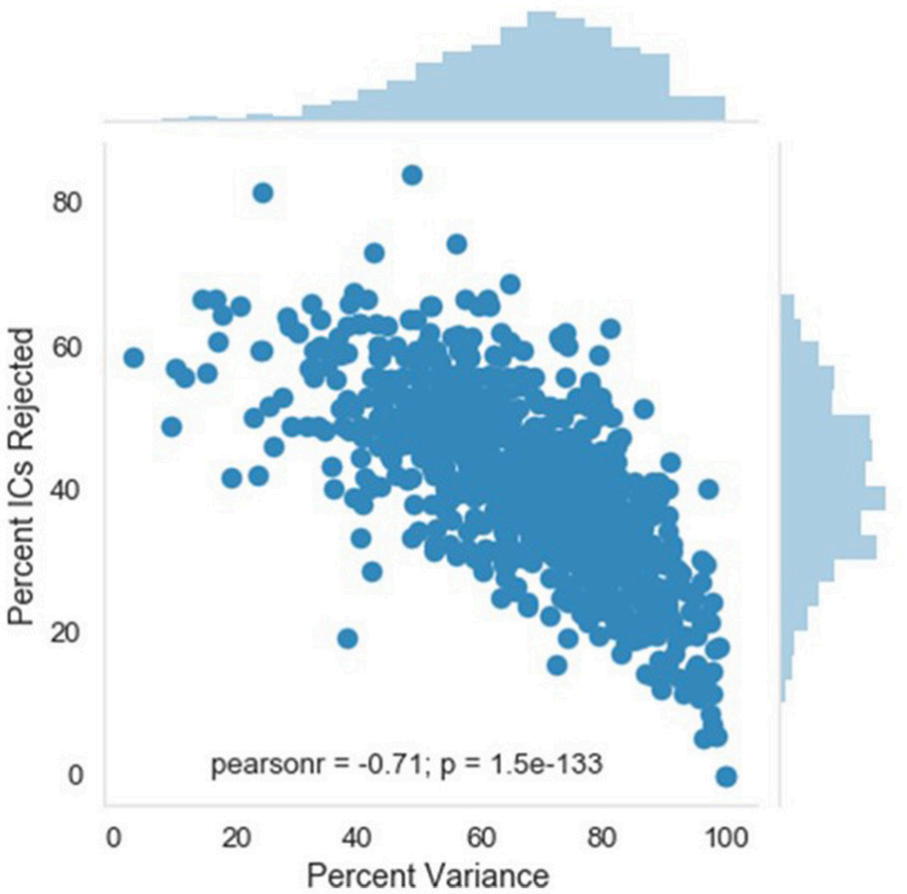
Relation between independent component (IC) rejection and the percent of data variance retained. The relation between the percent of variance in the EEG retained after MARA rejection of ICs (x-axis) and the percent of ICs rejected by MARA (y-axis) is shown for a developmental sample. The distributions for each metric in the same sample are shown opposite the labeled axes; top distribution is for percent of variance retained, right distribution is for percent of ICs rejected.

**Figure 5:**
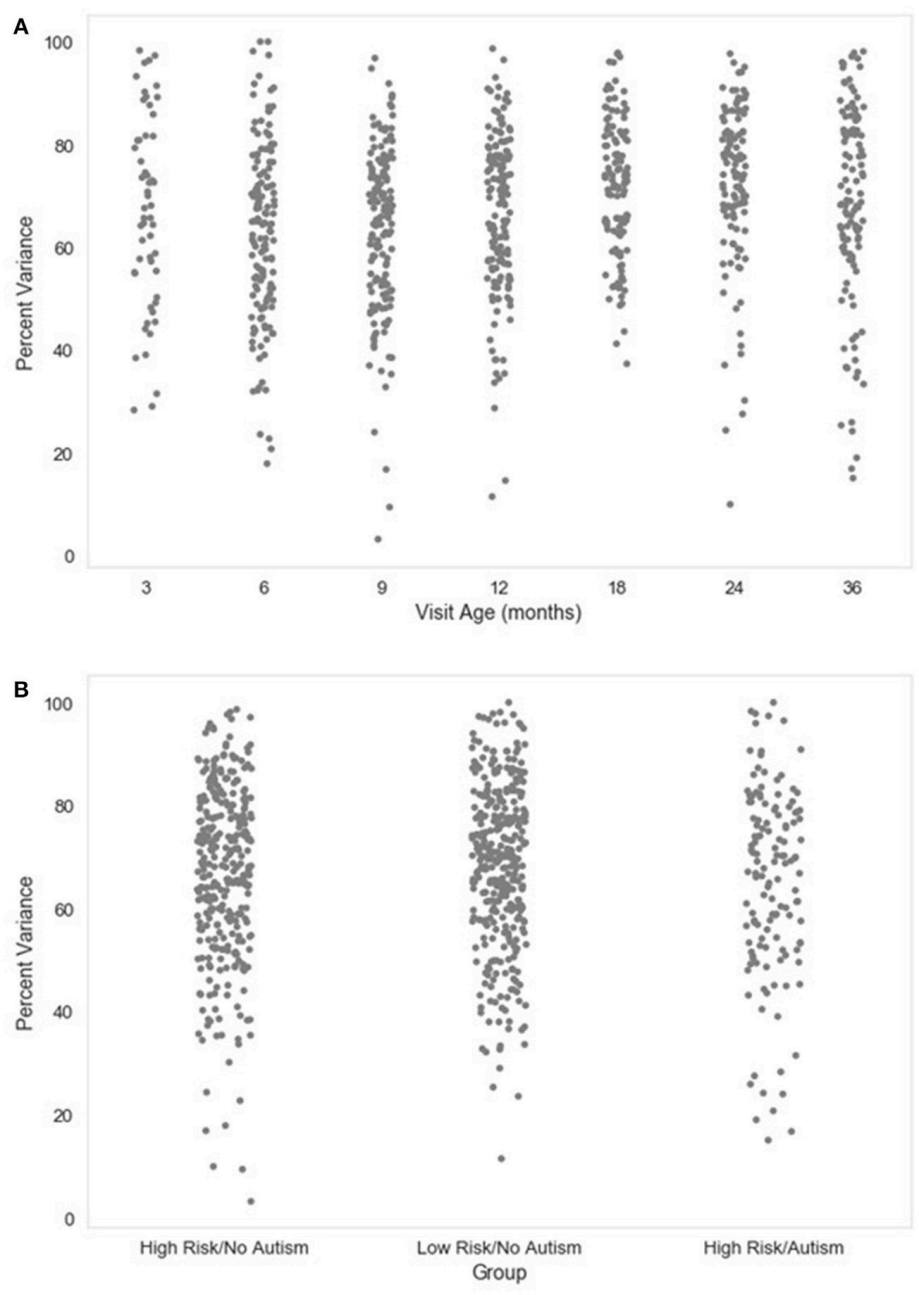
Percent variance retained post-MARA rejection. The distribution of the percent of variance in the EEG signal retained after MARA rejection of independent components is shown as a function of age when the EEG was acquired **(A)**, or clinical group status **(B)** for a developmental sample.

A complementary metric to the component rejection variables is the measure of variance in the data retained after MARA rejection relative to before MARA rejection of components. Here, the retained variance across all electrodes is calculated using the compvar function (Delorme et al., 2001) in EEGLAB. The amount of variance in the data that each independent component accounts for can be dramatically different across components for a file. Thus, a given percentage of components rejected does not necessarily indicate whether a small part of the EEG signal or even the majority of the EEG signal was removed. This relation is illustrated for the large developmental sample included here (Figure 4). Accordingly, the variance-kept metric may be useful to distinguish how much of the EEG signal was rejected by MARA, although users should take into consideration that artifact signal contributes more than neural signal to the signal variance in the first place. The variance-kept and the rejected component metrics together may be used to set a tolerance threshold (e.g., 20%) for the degree of data rejection with easy identification of files for removal from further analysis. For the distributions of the percent of EEG variance kept in the developmental sample included here, see Figure 5.

#### Median_artifact_probability_of_kept_ICs Artifact_probability_of_kept_ICs Range_artifact_probability_of_kept_ICs Min_artifact_probability_of_kept_ICs Max_artifact_probability_of_kept_ICs

**Figure 6:**
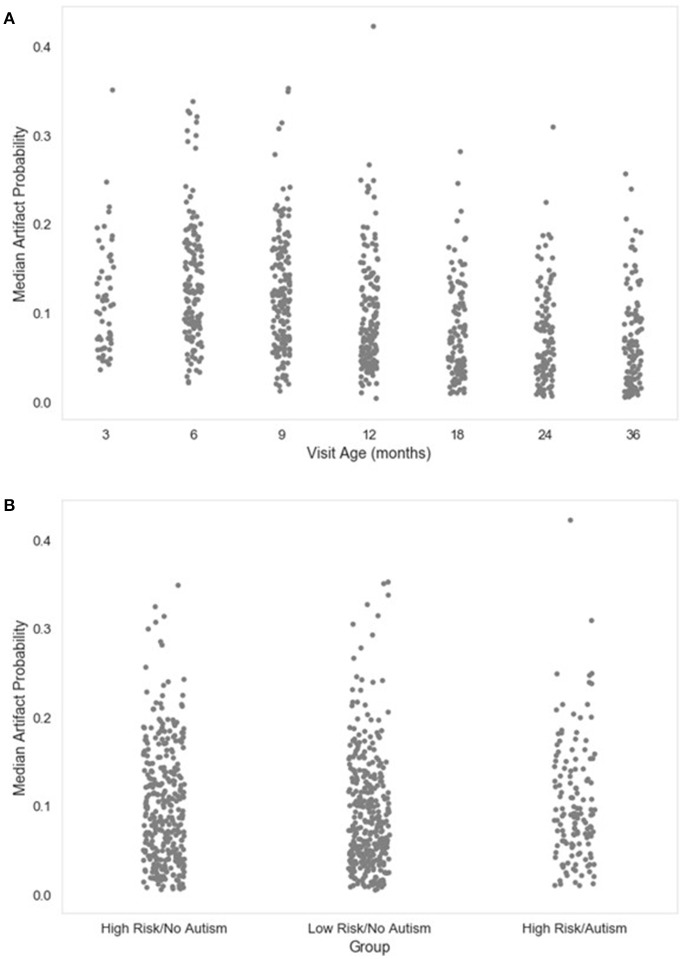
Median artifact probability of retained EEG. The distribution of the median artifact probability value for retained independent components post-MARA rejection is shown as a function of age when the EEG was acquired **(A)**, or clinical group status **(B)** for a developmental sample.

**Figure 7:**
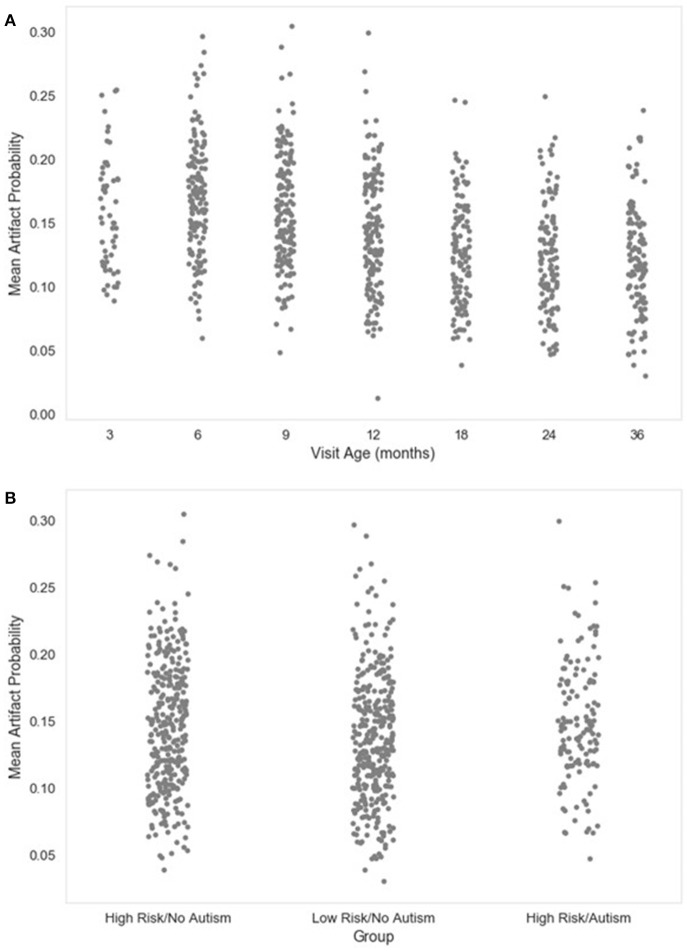
Mean artifact probability of retained EEG. The distribution of the mean (average) artifact probability value for the retained independent components post-MARA rejection is shown as a function of age when the EEG was acquired **(A)**, or clinical group status **(B)** for a developmental sample.

The final set of metrics provided by HAPPE include descriptive statistics for the MARA-generated probabilities that the independent components surviving rejection are artifact-contaminated (artifact probability metrics). It should be noted that these values are derived before any segment rejection or segment-level channel interpolation occurs, so these metrics will overestimate the artifact levels in the fully-processed data if either segment rejection or interpolation options have been selected. Still, the artifact probability metrics, especially the median and mean artifact probabilities, may inform which files remain too artifact-contaminated to contribute to further analyses. Distributions for the median and mean artifact probabilities for the developmental sample included here are provided (Figures 6, 7, respectively).

Through this report table of metrics, HAPPE therefore aims to provide a rich, quantifiable, yet easily accessible way to effectively evaluate data quality for even very large datasets in the context of automated processing. Visual examination of each file is not required, although it is available. Over and above the purposes of rejecting files that no longer meet quality standards for a study and evaluating HAPPE performance on a given dataset, we also hope to encourage more rigorous reporting of data quality metrics in manuscripts by providing these outputs already tabulated and easily transformed into descriptive statistics for inclusion. Users may also wish to include one or several of these metrics as continuous nuisance covariates in statistical analyses to better account for differences in data quality between files, or verify whether there are statistically significant differences in data quality post-processing between study groups of interest. For further guidance about using the processing report metrics to evaluate data, users may consult the HAPPE README file distributed with HAPPE software.

## Example analysis with HAPPE

In this section, the specifications for and results from one run of HAPPE with 10 developmental EEG files are provided. The EEG files contributing to this example dataset may be freely accessed through Zenodo at: https://zenodo.org/record/998965#.WdBg2BNSxBw. Acquisition parameters for each of the 10 data files are given in Table 3. In accordance with HAPPE's aim to deliver a processing strategy compatible with short EEG recordings, the median length of the 10 sample EEG files is only 3.8 min (with files ranging from 2.4 to 13 min). The HAPPE script with the configurations selected as run in this example is provided in Supplemental Materials (Please note that users will still need to change the path specifying the folder with the downloaded data to match the destination on their own machines). This iteration of HAPPE was implemented with MATLAB version 2017a and EEGLAB version 14.0.0b on an iMac running OS X El Capitan (Version 10.11.6) with one 2.7 GHz Intel Core i5 processor. This example analysis is included to enable users to replicate HAPPE performance independently and to facilitate implementing HAPPE with their own datasets after working through this example.

**Table 3:** Acquisition parameters for a 10-file example dataset.

**EEG identifier**	**Participant age (months)**	**EEG length (s)**	**EEG sampling rate (Hz)**	**EEG net size (No. of channels)**
baselinEEG01	6	349	250	128
baselineEEG04	36	189	250	128
baselineEEG05	18	164	250	128
baselineEEG06	3	784	250	128
baselineEEG07	3	145	250	128
baselineEEG08	12	279	500	128
baselineEEG09	12	161	500	128
baselineEEG10	24	349	500	128
baselineEEG11	9	184	250	128
baselineEEG12	6	284	250	128

**Figure 8:**
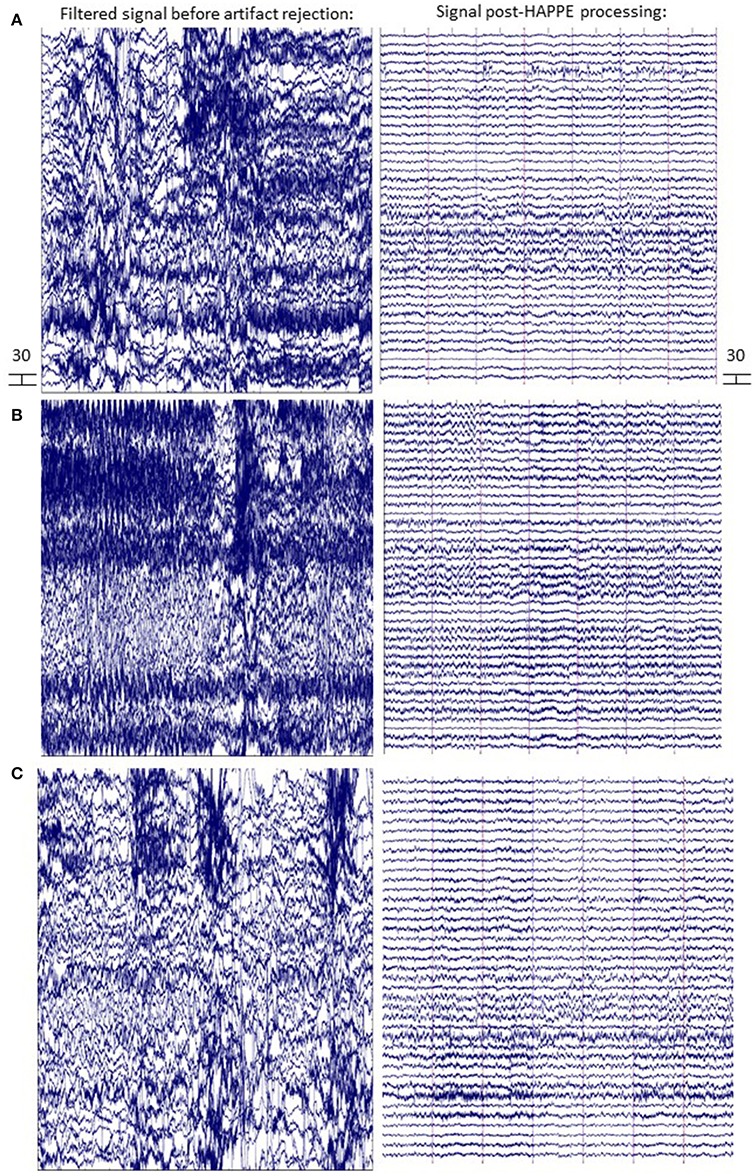
EEG signal before and after HAPPE processing. Three files from the example dataset are shown **(A-C)** with 14 s of data extracted from the first 30 s of the recording. The EEG signal after minimal processing (i.e., filtering, channel subset selection, and average re-referencing) is shown in the left panel. The EEG signal after HAPPE processing as described in the example analysis results section is shown in the right panel. All scales are in microvolts.

The following is a resting-state analysis of developmental EEG data collected with HydroCel 128-channel Geodesic Sensor Nets (EGI, Eugene, OR). The 39-channel subset selected for preprocessing contains bilateral frontal and temporal channels (in addition to the 10–20 channels), as these locations typically have the most extreme artifact levels, and therefore provide the greatest challenge to HAPPE's performance. To illustrate both the degree of artifact that occurs in these example files, and HAPPE performance for the same EEGs, data during the first 30 s of several EEGs is provided before and after HAPPE processing (Figure 8). HAPPE was run in the semi-automated setting to generate visualizations for each file at multiple steps in the pipeline (these visualizations for each example file are included in Supplemental Materials). Data were segmented into 2-s long segments with the segment rejection option selected, using artifact amplitude thresholds of −40 and 40 microvolts. Data were re-referenced using an average re-reference. It should be noted that during processing, ICA does not return identical results each time it is run on the same data because the decomposition solution searching begins with a randomly-generated weight matrix each time (Onton and Makeig, 2006). Therefore users replicating these analyses on the same data may find small discrepancies between their own set of results and those described here with respect to percent variance accounted for in each component, and MARA artifact probability assigned to each component (and accordingly, the number of components rejected by MARA, the artifact probabilities of remaining components, and the variance kept in the data post-MARA). The HAPPE processing report for this example analysis is presented in Table 1.

Data quality and HAPPE's effectiveness for this dataset are first discussed by way of the HAPPE processing report metrics. The first evaluation point for assessing data quality is the number of channels contributing uninterpolated data to further analysis (i.e., the good channels that weren't rejected during processing). In the present dataset, an average of 88% of the channels from the user-selected channel subset for each file were retained as good channels for further processing. Only one file contained fewer than 80% good channels (79%, baselineEEG11), with most of the bad channels covering the right frontal region of interest (ROI) for that file. This file may merit removal from further analysis depending on the ROI(s) to be examined (i.e., frontal ROIs or temporal ROIs). For this processing example exclusive of specific statistical analyses, baselineEEG11 is retained.

Next, the effectiveness of W-ICA and ICA in segregating and rejecting artifact from the neurophysiological signal are evaluated using the post-MARA data metrics. For this sample of 10 files, on average, 42% of the components were rejected per file during HAPPE, comparable to both the rate of MARA component rejection in adult EEG (Winkler et al., 2014) and the rate of manual component rejection with infant EEG data (Piazza et al., 2016). Moreover, following component rejection, 68% of the EEG variance was retained per file on average, suggesting MARA-based component rejection did not result in an unacceptable level of data loss generally. Only one file (baselineEEG9) retained a minimal amount of variance post-MARA rejection (24% variance retained), meriting further review or visualization of the data to determine whether it should be removed from post-processing analyses. (It was retained in the dataset for this example analysis). Additionally, on average, the median and mean artifact probabilities of the retained components post-MARA rejection was only 0.10 and .13, respectively, suggesting W-ICA and ICA with MARA achieved robust segregation of neurophysiological and artifact signals during the decomposition in the developmental dataset. No individual files post-MARA rejection had average or median artifact probabilities greater than 0.30, or even a more conservative 0.25, therefore no files were considered for removal from further analyses based on residual artifact at this step in the pipeline.

Finally, the number of segments retained after the segment rejection step, if selected, may be evaluated to determine whether any files can no longer contribute sufficient data for further analyses. For the present sample, each EEG file was segmented into 2-s long windows and each segment was evaluated for rejection. For the present sample, an average of 66% of the 2-s segments were retained per EEG file after this step. Post-segment rejection, no individual file had fewer than 20 retained segments (average number of retained segments = 99), a sufficient number of segment samples for calculating power (Cuevas et al., 2014; Gabard-Durnam et al., 2015), so no individual files were considered for removal from post-processing analyses. Therefore, despite initial high levels of artifact contamination in the dataset, all 10 files were successfully processed in HAPPE with the potential for inclusion in further analyses.

**Figure 9:**
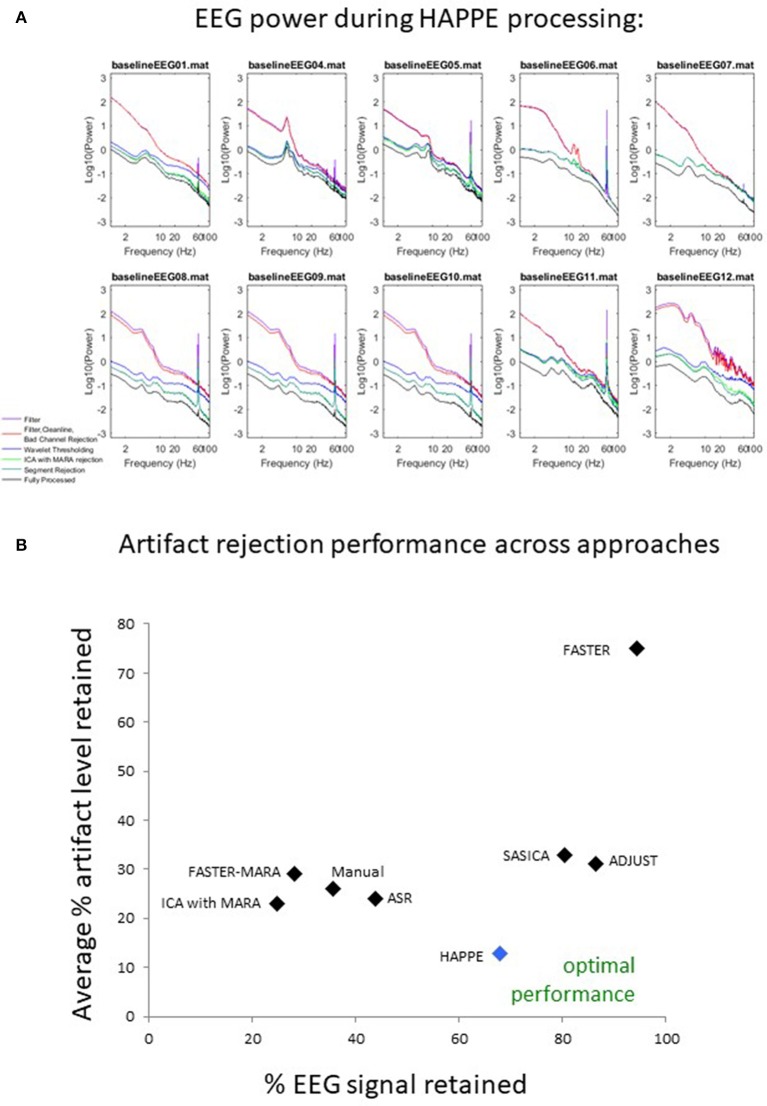
Results from HAPPE processing steps and comparison to alternative approaches. For each file in the example dataset, the EEG power (y-axis, in microvolts squared) across a range of EEG signal frequencies (x-axis) is shown as a function of several processing steps within HAPPE. Power spectrums are generated after the filtering step (filter), after basic preprocessing (filter, CleanLine, bad channel rejection), wavelet-enhanced independent component analysis (wavelet thresholding), independent component analysis with MARA rejection (ICA with MARA rejection), segment rejection for the retained data (segment rejection), and after the final channel interpolation and re-referencing steps (fully-processed) **(A)**. All 8 approaches for artifact rejection are compared in terms of the percent EEG data variance retained (x-axis) and the average artifact level in the retained EEG data (y-axis), where optimal performance would place an approach near the bottom right corner of the chart, retaining most of the EEG variance with low levels of artifact **(B)**.

Data quality may also be assessed subjectively for these 10 files by examining the EEG power spectrum for each file after each processing step in HAPPE (see Figure 9). Post-HAPPE processing, there is a dramatic reduction in the electrical noise signal (60 Hz spike) across the 10 files, relative to the earliest stages of processing. In several cases (especially baselineEEG06 and baselineEEG07), alpha peaks in power, a normative feature of EEG power spectrums, are revealed post-HAPPE processing relative to the earliest processing steps. Moreover, baselineEEG12 initially contained a very large amount of artifact, visible in the power spectrum for frequencies greater than 20 Hz, that is robustly removed during HAPPE processing. Across the 10 files, the power spectrums post-HAPPE processing appear more uniform in shape and scale than those for the earliest processing steps, where artifact contamination had not yet been addressed. Therefore, through visual inspection of the power spectrums for the 10 example files, no individual file post-HAPPE processing appears to merit removal from further analyses. When HAPPE is run in the semi-automated setting, as in this example analysis, users may monitor these power spectrum features generated post-HAPPE processing for their own files as well.

## HAPPE compared to other common processing approaches

**Figure 10:**
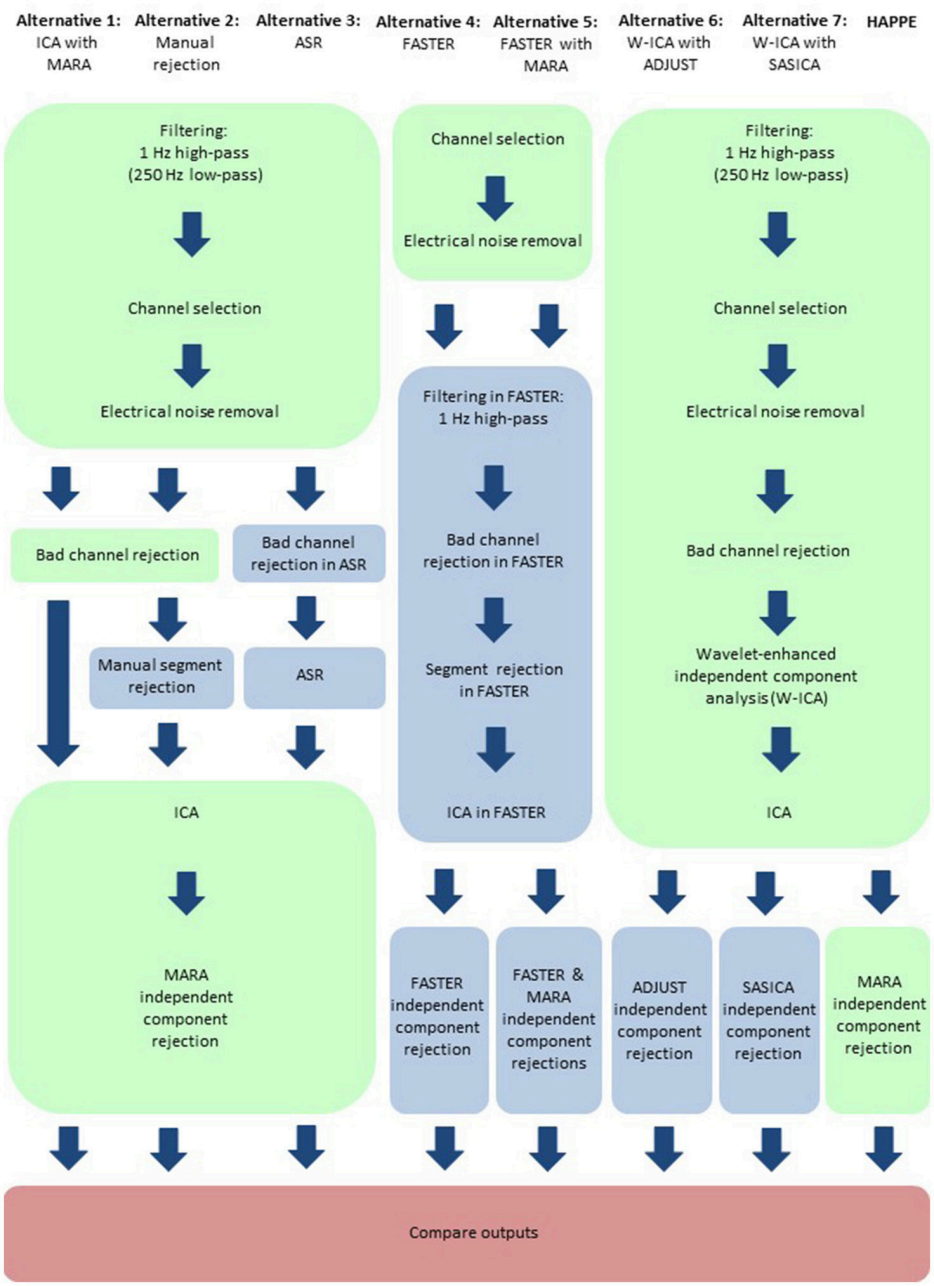
Schematic illustrating the HAPPE pipeline in relation to seven alternative processing approaches. Processing steps that are consistent across approaches, and implemented in HAPPE, are highlighted in light green. Processing steps that are unique to the alternative approaches are highlighted in light blue. Independent component analysis is abbreviated to ICA. Multiple Artifact Rejection Algorithm is abbreviated to MARA. Wavelet-thresholded ICA is abbreviated to W-ICA. Artifact Subspace Reconstruction is abbreviated to ASR. Fully Automated Statistical Thresholding for EEG artifact Rejection is abbreviated to FASTER. Automatic EEG artifact Detector based on the Joint Use of Spatial and Temporal features is abbreviated to ADJUST. SemiAutomated Selection of Independent Components for Artifact Correction in the EEG is abbreviated to SASICA.

Here, HAPPE is compared to seven alternative processing approaches for removing experimental and participant-induced artifact with the same 10 developmental files. Of these alternative approaches, one (FASTER) is a comprehensive processing pipeline that, like HAPPE, takes raw EEG files as inputs and produces fully-processed EEG data suitable for analyses. The remaining six alternative approaches consist of artifact-rejection steps. Therefore, for these alternative artifact-rejection approaches, data are processed in HAPPE for all steps besides the alternative rejection steps. Each approach's performance is evaluated using the data quality metrics provided in the HAPPE processing report for consistency across methods (Figure 9B). Specifically, the percent of independent components rejected by each method and the percent of EEG variance retained after component rejection metrics quantify EEG signal preservation in the artifact rejection process (summarized in Table 4), while the mean and median artifact probability metrics of retained components index successful artifact rejection (summarized in Table 5). The most successful approaches should combine high percentages of retained EEG variance and components with low artifact estimates in the retained data, indicating excellent separation and rejection of artifacts from the EEG signal of interest. Alternatively, approaches with either high or low percentages of variance and components retained alongside high levels of retained artifact indicate incomplete artifact rejection. Student's paired sample *t*-tests were performed on the data metrics for formal comparisons between approaches. The comparison between HAPPE and each alternative approach is provided below (Figure 9B, and see Figure 10 for a summary of each approach's processing steps).

**Table 4:** Data quality metrics measuring the amount of EEG data that is retained post-processing compared for HAPPE and alternative approaches.

**Filename**	**Percent independent components rejected**								**Percent variance kept after rejection**							
	**HAPPE**	**ICA**	**Manual**	**ASR**	**ADJUST**	**SASICA**	**FASTER**	**FASTER-MARA**	**HAPPE**	**ICA**	**Manual**	**ASR**	**ADJUST**	**SASICA**	**FASTER**	**FASTER-MARA**
baselineEEG01	50	97.37	92.11	82.86	26.32	44.74	5.26	94.44	48.28	1.90	4.21	50.59	85.90	63.13	96.10	4.17
baselineEEG04	38.89	80.56	72.22	64.10	38.89	38.89	2.70	63.89	80.39	37.85	52.92	80.30	95.08	80.10	97.55	21.87
baselineEEG05	37.14	45.71	37.14	53.85	8.57	20.00	5.26	38.89	79.08	74.28	77.48	69.27	78.61	87.49	93.89	80.12
baselineEEG06	2.94	82.35	82.35	81.58	17.65	26.47	2.63	70.27	98.07	52.89	26.15	37.97	96.44	89.65	96.53	55.86
baselineEEG07	2.78	97.22	100	86.49	11.11	11.11	5.41	85.71	99.13	2.50	0	18.88	95.42	91.87	89.00	24.91
baselineEEG08	75.76	96.97	87.88	83.78	15.15	18.18	2.70	83.33	43.67	8.86	22.28	43.02	74.07	86.66	93.28	16.89
baselineEEG09	71.43	97.14	74.29	79.49	62.86	22.86	2.70	86.11	24.25	7.56	30.38	33.58	78.77	77.17	93.28	16.26
baselineEEG10	43.75	78.13	53.13	61.54	53.13	56.25	2.70	83.33	83.82	23.12	70.68	49.18	84.13	62.04	93.40	17.93
baselineEEG11	35.48	90.32	48.39	74.36	16.13	19.35	2.70	75.00	85.15	37.73	59.9	42.41	79.79	89.89	95.75	41.54
baselineEEG12	62.86	97.14	88.57	94.44	54.29	37.14	2.63	94.59	35.67	1.33	11.89	13.53	96.86	75.96	94.46	2.39
**Dataset average**	**42.10**	86.29^***^	73.61^*^	76.25^**^	30.41	29.50	3.47^**^	77.56^**^	**67.75**	24.80^***^	35.59^*^	43.87^*^	86.51^*^	80.40	94.32^*^	28.19^**^

* *p < 0.05*.

** *p < 0.01*.

*** *p < 0.001. Significant differences between approaches where HAPPE shows the more favorable result are bolded*.

**Table 5:** Data quality metrics measuring the amount of artifact retained post-processing in the EEG data compared for HAPPE and alternative approaches.

**Filename**	**Mean artifact probability of retained components**								**Median artifact probability of retained components**							
	**HAPPE**	**ICA**	**Manual**	**ASR**	**ADJUST**	**SASICA**	**FASTER**	**FASTER-MARA**	**HAPPE**	**ICA**	**Manual**	**ASR**	**ADJUST**	**SASICA**	**FASTER**	**FASTER-MARA**
baselineEEG01	0.14	0.25	0.41	0.18	0.41	0.28	0.87	0.35	0.10	0.25	0.43	0.19	0.26	0.11	0.93	0.35
baselineEEG04	0.16	0.20	0.15	0.20	0.27	0.30	0.63	0.22	0.16	0.2	0.12	0.18	0.20	0.19	0.78	0.29
baselineEEG05	0.08	0.28	0.21	0.25	0.35	0.35	0.46	0.25	0.01	0.34	0.18	0.26	0.10	0.10	0.41	0.28
baselineEEG06	0.05	0.21	0.36	0.30	0.03	0.03	0.67	0.31	0.03	0.22	0.43	0.27	0.03	0.02	0.76	0.38
baselineEEG07	0.05	0.45		0.26	0.04	0.04	0.79	0.36	0.05	0.45		0.24	0.04	0.05	0.89	0.34
baselineEEG08	0.21	0.13	0.23	0.32	0.66	0.69	0.82	0.30	0.25	0.13	0.20	0.28	0.76	0.77	0.94	0.32
baselineEEG09	0.15	0.26	0.21	0.25	0.52	0.63	0.82	0.30	0.08	0.26	0.20	0.23	0.46	0.83	0.94	0.36
baselineEEG10	0.16	0.1	0.24	0.14	0.13	0.14	0.82	0.31	0.12	0.1	0.31	0.03	0.08	0.08	0.95	0.36
baselineEEG11	0.07	0.35	0.25	0.33	0.23	0.23	0.71	0.23	0.03	0.41	0.23	0.45	0.04	0.03	0.86	0.22
baselineEEG12	0.23	0.12	0.33	0.15	0.47	0.57	0.89	0.27	0.18	0.12	0.33	0.15	0.37	0.56	0.96	0.27
**Dataset average**	**0.13**	**0.23**^∓^	0.26^**^	0.24^*^	0.31^**^	0.33^**^	0.75^***^	0.29^***^	**0.10**	0.25^*^	0.27^*^	0.23^*^	**0.23**^∓^	**0.27**^∓^	0.84^***^	0.32^***^

∓ *p < 0.1*.

* *p < 0.05*.

** *p < 0.01*.

*** *p < 0.001. Significant differences between approaches where HAPPE shows the more favorable result are bolded*.

### Independent component analysis (ICA)

The ICA alternative approach remains fully-automatable, and simply removes the wavelet-thresholding step from the processing sequence of HAPPE. That is, data are fed directly into a single ICA decomposition for the purpose of rejecting artifact. Like HAPPE, this alternative approach does not sacrifice any data segments during artifact rejection, an appealing feature for processing short data recordings. Data were processed in HAPPE for the initial steps of filtering, channel subset selection, line noise removal through CCleanLine, and bad channel detection, as these steps may all improve ICA decomposition performance (Winkler et al., 2015). Where the wavelet-thresholding step follows in HAPPE, data were instead extracted from the HAPPE pipeline and input to ICA with the same extended Infomax algorithm settings as in HAPPE. MARA component rejection was then carried out using the same settings as in HAPPE. Data post-MARA processing were then compared to HAPPE post-MARA data (before segment rejection) (see Tables 4, 5).

First, independent component rejection rates and retained variance were compared across approaches as an indicator of how well the ICA segregated artifact from signal. The ICA alternative approach rejected significantly more components (86.3%) compared to HAPPE (42%) [*t*_(9)_ = 5.31, *p* = 0.00058, *n* = 10]. For half of the files (5), only a single component was preserved as neurophysiological signal, an unacceptably low retention rate. The ICA approach also removed a significantly greater amount of the variance from the data than HAPPE [*t*_(9)_ = 5.48, *p* = 0.00039, *n* = 10], leaving only 24.8% of the data's variance on average, barely above the rejection threshold for evaluating HAPPE. Next, to assess the quality of the retained data post-MARA rejection across approaches, the median and mean artifact probability metrics were compared. Despite the higher rates of component rejection, the remaining components after the ICA alternative approach had median and mean artifact probabilities about twice as large as the components after HAPPE processing [median artifact probability: *t*_(9)_ = 2.52, *p* = 0.0033, *n* = 10; mean artifact probability: *t*_(9)_ = 2.07, *p* = 0.068, *n* = 10]. Applying even the more liberal retained-artifact probability threshold of 0.3 used to evaluate HAPPE performance, the ICA approach resulted in 3 files meeting criteria for removal from further analyses, compared to 0 files in HAPPE. Overall, the alternative ICA approach without an artifact rejection step preceding it (like W-ICA in HAPPE) performed much more poorly on the developmental files relative to HAPPE, rejecting far more components but also retaining higher artifact levels in the data.

### Manual segment rejection with ICA (manual)

The manual alternative approach reintroduces artifact rejection before the ICA decomposition and MARA rejection steps. However, the manual approach is not automatable and results in data loss from files that already have short lengths. For the manual approach, data files were processed in HAPPE for filtering, channel subset selection, line noise removal, and bad channel detection. Data was then removed from HAPPE and segmented into 2-s segments for manual artifact rejection (by an experimenter with extensive manual EEG processing training who was also blind to the HAPPE results). After manual rejection, each file was input to ICA (as in HAPPE) with MARA component rejection.

HAPPE retained more EEG data than the manual rejection approach across all measures. First, as the manual approach involves segment rejection, the number of retained data segments was compared to the retained segment count after the optional, automated segment rejection step in HAPPE. HAPPE retained a significantly greater number of data segments relative to the manual segment rejection approach [*t*_(9)_ = 3.11, *p* = 0.0125, *n* = 10], preserving 2.9 times the number of segments retained through manual rejection (HAPPE mean segment number: 98.9; manual segment number: 33.6). The manual approach left 3 files with fewer than 20 segments, which may be insufficient for further post-processing analyses. The manual approach also resulted in MARA rejecting significantly more ICA components than HAPPE [*t*_(9)_ = 3.02, *p* = 0.0145, *n* = 10], and retained significantly less data variance after component rejection [*t*_(9)_ = 3.18, *p* = 0.0112, *n* = 10], keeping only about half of the variance retained through HAPPE on average. Indeed, 3 files processed with the manual approach did not retain the minimum of 20% of the data variance to be included in post-processing analyses (criteria set for evaluating HAPPE performance previously). Despite the higher rates of data rejection, the manual approach also had median and mean artifact probabilities more than twice as large as those obtained in HAPPE, [median artifact: *t*_(8)_ = 3.15, *p* = 0.0117, *n* = 9; mean artifact: *t*_(8)_ = 3.42, *p* = 0.0070, *n* = 9]. Accordingly, half of the files had artifact probabilities above the 0.3 threshold for rejection from further analyses. Therefore, in addition to the disadvantage of requiring manual input for each file, the manual approach did not result in either greater retained data or better retained data quality compared with HAPPE.

### Artifact subspace reconstruction (ASR) with ICA

The ASR approach interpolates artifact-contaminated regions in continuous data, guided by a period of clean data within the same file (here, clean data was determined by the ASR algorithm). Although ASR remains fully-automatable, unlike HAPPE, it is not appropriate for event-related EEG processing. Still, the performance of ASR was compared to HAPPE for processing resting-state EEG data. Data were processed as in HAPPE for filtering, channel selection, and electrical noise removal steps. The ASR approach was then carried out with EEGlab's Clean_rawdata functions adopted from BCILAB (Kothe and Makeig, 2013; Mullen et al., 2015). Clean_rawdata contains bad channel rejection and ASR artifact interpolation steps. Channels were removed if they were flat for more than 10 s or had correlations below 0.8 with the other channels' data. Channels were not removed in the presence of line-noise due to the prior use of CleanLine. ASR was used to interpolate artifact “bursts” with variance more than 5 standard deviations different from the automatedly detected clean data, as in prior work with clinical populations (Grummett et al., 2014). Data segments post-interpolation were removed with a time-window rejection setting of 0.05 (aggressive segment rejection). Data were then submitted to ICA and MARA component rejection (as in HAPPE).

HAPPE retained more EEG data than the ASR approach across all measures. Although ASR was designed for brief “bursts” of artifact in otherwise clean data, due to the high degree of artifact contamination in the developmental data, the ASR approach interpolated an average of 35.7% of the EEG data per file, which may constitute a prohibitively high interpolation rate. Even with the high rate of artifact interpolation, MARA rejected far higher percentages of independent components [*t*_(9)_ = 4.86, *p* = 0.001, *n* = 10] and retained far less EEG data variance [*t*_(9)_ = 4.02, *p* = 0.003, *n* = 10] on the ASR-treated data than in HAPPE. The ASR approach also retained higher mean [*t*_(9)_ = 3.04, *p* = 0.014, *n* = 10] and median [*t*_(9)_ = 2.55, *p* = 0.031, *n* = 10] artifact levels in the data post-component rejection compared to HAPPE. Consequently, the ASR approach resulted in half of the example files meeting either the retained variance or retained artifact criteria for removal from further analyses, Thus, by rejecting more EEG data but also retaining higher levels of artifact, the ASR approach performed less successfully than HAPPE across all measures in the context of developmental resting-state EEG files.

### Fully automated statistical thresholding for EEG artifact (FASTER)

FASTER is a fully-automated pipeline that transforms EEG data from raw files to processed data inputs for analyses, with artifact rejection steps implemented here at the channel, epoch, and independent component levels. For each rejection step, FASTER calculates statistical parameters for the data and rejects channels, epochs, or components with Z-scores above a pre-specified threshold as outliers (here the default settings of Z-scores of 3 were applied). FASTER's single-channel, single-epoch interpolation step was not included in the comparison to HAPPE because this option has been integrated into HAPPE (an optional segment rejection setting) with acknowledgement of FASTER for this capability. FASTER performs optimally with higher numbers of data points and channels, and assumes normal distributions of uncontaminated EEG data, conditions that may not be met with highly-contaminated, short EEG recordings. FASTER also uses information from EOG channels as one means to reject independent components, but EOG channel recordings are not typically performed with developmental populations (including the present data files), which may further impair FASTER performance in this context. Here, data were passed through the channel selection and CleanLine line noise removal steps first as in HAPPE. Data were then input to FASTER, where filtering, channel rejection, segmentation (into regular 2-s epochs as in HAPPE), segment rejection, and ICA with component rejection steps were performed. To generate the retained artifact metrics for FASTER-processed data for comparison across approaches, data were then classified (but not rejected) according to the 6 artifact features in MARA.

The FASTER approach retained more data than HAPPE, but also retained much higher rates of artifact. Specifically, FASTER rejected a lower percentage of independent components [*t*_(9)_ = −4.79, *p* = 0.001, *n* = 10] and retained significantly more EEG data variance [*t*_(9)_ = 3.07, *p* = 0.013, *n* = 10]. However, FASTER also retained far higher mean [*t*_(9)_ = 17.36, *p* = 0.000000031, *n* = 10] and median [*t*_(9)_ = 16.14, *p* = 0.00000006, *n* = 10] artifact levels in the data, with mean artifact levels almost 6 times higher and median artifact levels more than 8 times higher than those found in HAPPE -processed data. Every one of the example files met artifact retention criteria for removal from further analyses post-FASTER processing. This pattern of high levels of retained data together with high levels of retained artifact through FASTER processing is consistent with incomplete artifact segregation and rejection. Thus, in the context of developmental EEG files, HAPPE outperforms FASTER in rejecting EEG artifact.

### FASTER with MARA component rejection (FASTER-MARA)

Next, we tested whether combining the FASTER approach with MARA independent component rejection would lead to improved artifact rejection for developmental EEG files compared to the combination of W-ICA with MARA rejection in HAPPE. Data were processed as above in FASTER [see section Fully Automated Statistical Thresholding for EEG Artifact (FASTER)] and all retained independent components were then subjected to MARA classification and rejection. The combined FASTER-MARA approach rejected significantly more components [*t*_(9)_ = 4.4, *p* = 0.002, *n* = 10] and retained far less EEG data variance than HAPPE [*t*_(9)_ = −5.23, *p* = 0.001, *n* = 10]. Moreover, the FASTER-MARA approach resulted in higher mean [*t*_(9)_ = 7.41, *p* = 0.00018, *n* = 10] and median [*t*_(9)_ = 7.41, *p* = 0.000040, *n* = 10] artifact levels in the preserved components compared to HAPPE. Indeed, 6 of the 10 example files met either the retained variance or retained artifact criteria for removal from further analyses using the FASTER-MARA approach. Therefore, HAPPE retained EEG data variance while rejecting EEG artifact more successfully than the FASTER-MARA approach for these developmental EEG files.

### W-ICA with automatic EEG artifact detection based on the joint use of spatial and temporal features (W-ICA with ADJUST)

ADJUST is a fully-automated algorithm for classifying and rejecting independent components using a combination of spatial and temporal data features optimized for detecting blinks, eye movements, and generic discontinuities. Importantly, ADJUST was not designed to robustly detect other kinds of artifact, including EMG artifact that frequently occurs in developmental and patient populations. Here, filtering, channel selection, electrical noise removal, bad channel rejection, W-ICA, and ICA steps were all performed as in HAPPE to optimize the ICA decomposition classified by ADJUST. Data were then subjected to ADJUST component rejection instead of MARA-based rejection. As with the FASTER approach, retained artifact metrics for comparison across approaches were then generated through the artifact feature classification in MARA (without component rejection).

The ADJUST approach retained somewhat more EEG data variance than HAPPE [*t*_(9)_ = 2.33, *p* = 0.044, *n* = 10], although it did not reject significantly fewer components than HAPPE [*t*_(9)_ = −1.64, *p* = 0.14, *n* = 10]. However, ADJUST retained higher mean [*t*_(9)_ = 3.48, *p* = 0.007, *n* = 10] and median [*t*_(9)_ = 2.27, *p* = 0.050, *n* = 10] artifact levels compared to HAPPE, and half of the example files met the artifact retention criteria for removal from further analysis with the ADJUST approach. Taken together, this pattern of increased data variance retention along with increased artifact levels suggests more incomplete artifact segregation and rejection during ADJUST compared with HAPPE, most likely due to the EMG artifacts that ADJUST was not designed to robustly eliminate.

### W-ICA with semiautomatic selection of independent components for artifact correction in EEG (W-ICA with SASICA)

The last approach compared to HAPPE was SASICA, a semi-automated software for classifying and rejecting independent components using a combination of data features and statistical thresholds to guide manual component rejection. SASICA was not designed to be fully-automated, a drawback in processing large datasets. Unfortunately, as in FASTER, SASICA performs optimally when EOG channel information and dipole fit information is present, which is typically not present or reliably calculated for developmental data. Thus, the observed SASICA performance with developmental EEG may not reflect its entire capability with the lower-artifact, adult EEG data on which it was tested. Here, filtering, channel selection, electrical noise removal, bad channel rejection, W-ICA, and ICA steps were all performed as in HAPPE to optimize the ICA decomposition classified by SASICA. Data were then segmented into regular 2-s epochs and subjected to SASICA component classification, with manual rejection of all recommended components. All features that could be calculated with the developmental example files were employed to classify components, including the autocorrelation feature (with automatic correlation threshold with 20 ms lag selected), focal component feature (automatic Z threshold selected), focal trial activity (automatic Z threshold selected), signal to noise ratio feature (with default settings and threshold ratio of 1), and correlation with other channels feature (automatic comparison to other channels and threshold of 4). Retained artifact metrics for comparison across approaches were then generated as before through artifact feature classification in MARA without rejection.

SASICA did not significantly differ from HAPPE in the amount of EEG signal retained post-component rejection by any metric. That is, SASICA did not statistically differ from HAPPE in either the percent of components rejected [*t*_(9)_ = −1.530, *p* = 0.160, *n* = 10] or the amount of EEG variance retained after rejection [*t*_(9)_ = 1.61, *p* = 0.143, *n* = 10]. However, SASICA retained significantly higher mean [*t*_(9)_ = 3.302, *p* = 0.009, *n* = 10] artifact levels after component rejection compared with HAPPE. Indeed, half of the data files met the artifact retention criteria for removal from further analysis with the SASICA approach. There was also a trend-level difference with higher median artifact levels in retained data through SASICA compared with HAPPE [*t*_(9)_ = 1.96, *p* = 0.082, *n* = 10]. Thus, while SASICA retains comparable amounts of EEG variance, the higher level of retained artifact makes it less successful than HAPPE processing developmental data.

## Discussion

EEG recordings like those collected with developmental populations present particular challenges from a data processing perspective, as they typically contain a high degree of artifact contamination, can by necessity be shorter than recordings collected in adults, and are often recorded in the absence of polygraphic signals for localizing physiological artifact. Multiple toolboxes and pipelines exist for various steps of EEG processing (e.g., FASTER, SASICA, ADJUST, ASR, TAPEEG), but these softwares are often optimized for conditions that are not met for these EEG classes due to data's constraints. To date, there is a paucity of resources tested with or targeting EEG data with high-artifact levels, short recording lengths, or absent physiological signal co-recordings. As demonstrated above, direct application of contemporary processing strategies used with typically-functioning adults, like ICA, without additional pre-processing considerations are not effective under these short-length, high-noise conditions. However, as fields move toward larger sample sizes with higher-density EEG channel layouts, the traditional manual data rejection approach used in many labs is becoming unsustainable. Moreover, despite the heightened importance of EEG data quality monitoring and reporting in fields with the highest rates of artifact contamination, no standardized metrics are currently systematically referenced in the literature (Cuevas et al., 2014; Keil et al., 2014).

HAPPE addresses each of these challenges as an automated EEG pipeline optimized for short recordings and/or high levels of artifact. HAPPE also encourages standardized reporting of processing performance through its report of data quality metrics and the sample distributions for these metrics from a large developmental dataset for reference. Evaluation with these data quality metrics revealed that HAPPE's combination of W-ICA and ICA with MARA-based component rejection outperformed seven alternative artifact rejection approaches under conditions of high artifact, short EEGs. That is, relative to these other approaches, HAPPE both rejected a greater proportion of artifacts and in almost all cases, simultaneously preserved a greater proportion of the underlying signal. HAPPE also retained more files per dataset with sufficient data for analyses than any of the alternative approaches. This robust performance achieved with the small example dataset is supported by the post-HAPPE data quality metric distributions across a much larger developmental dataset of 867 files. HAPPE thus constitutes a robust approach and pipeline to meet the growing need for automated, accessible pipelines for EEG processing, especially for developmental neuroscience and psychology fields.

There are several limitations to HAPPE that should also be considered. Foremost is that in most cases users must select a subset of channels for processing in HAPPE ashigh-density EEG recordings in developmental samples most likely will not meet the data-length requirements for robust ICA decomposition without a dimension reduction step. An alternative reduction approach to channel subset selection is to perform principal component analysis (PCA) on the entire channel set and then pass a subset of the resulting PCA components to ICA (instead of channel-level data). It should be noted, though, that the PCA approach introduces nonlinearities into the data (a slight corruption of the signal), and through selecting a PCA component subset, some amount of brain-origin signal is discarded with the rejected components (Onton and Makeig, 2006). For these reasons, the current version of HAPPE implements the channel subset approach, ensuring the entirety of the selected channels' data is processed and preserved as native EEG signal. A second limitation is that HAPPE is not currently suitable for preprocessing data intended for Event-related potential (ERP) analyses, due to its utilization of a 1 Hz high-pass filter (Acunzo et al., 2012). A complimentary HAPPE pipeline appropriate for ERP pre-processing is currently being developed and will be made publically available. Lastly, the amplitude of the EEG signal (and thus any EEG power estimates) is often decreased through both W-ICA and ICA approaches. Prior research has also found power spectrum and coherence measure distortions after using ICA. However, these disruptions are improved using the W-ICA approach (although formal statistical comparisons were not reported; Castellanos and Makarov, 2006). To the extent that W-ICA reduces the number of components rejected through subsequent ICA in HAPPE, this potential distortion may be reduced relative to alternative ICA approaches in HAPPE as well. In the present example files, the EEG signal morphology and shape of the EEG power spectrum appear preserved (see also Levin et al., under review, for illustrations of this effect). Still, he magnitude of absolute (raw) power values generated on HAPPE-processed EEG data should not be directly compared to those from data processed without either W-ICA or ICA due to the established differences in signal magnitude.

HAPPE is freely available, covered under the terms of the GNU General Public License (version 3) (Free Software Foundation, 2007). HAPPE can be used as stand-alone software as presented here, and through the Batch EEG Automated Processing Platform (BEAPP) software (see accompanying manuscript submission to this issue). HAPPE and associated files may be accessed at: https://github.com/lcnhappe/happe.

## Author contributions

LG-D conceived of HAPPE and drafted the manuscript. LG-D, AM contributed and tested code. LG-D, AM, CW, and AL contributed to the design of HAPPE, analysis, and interpretation of data, critically revised the manuscript for intellectual content, and approved the final version for submission.

### Conflict of interest statement

The authors declare that the research was conducted in the absence of any commercial or financial relationships that could be construed as a potential conflict of interest.
