# Supplementary material for: The Harvard Automated Processing Pipeline for Electroencephalography (HAPPE): Standardized Processing Software for Developmental and High-Artifact Data
Source: Front Neurosci. 2018 Feb 27;12:97. doi: 10.3389/fnins.2018.00097 (PMC5835235; doi:10.3389/fnins.2018.00097)
Supplement: Supplementary file 1 [file DataSheet1.PDF]

```
%~~~~~~~~~~~~~~~~~~~~~~~~~~~~~~~~~~~~~~~~~~~~~~~~~~~~~~~~~~~~~~~~~~~~~~~~~~
% The Harvard Automated Pre-Processing Pipeline for EEG (HAPPE)
% Version 1.0
%
% Developed at Boston Children's Hospital Labs of Cognitive Neuroscience
%
% For a detailed description of the pipeline and user options, please see the
following manuscript:
% Gabard-Durnam, et al., (submitted).To be updated upon acceptance for
publication.
% Contributors to HAPP-E:
%
% Laurel Joy Gabard-Durnam (laurel.gabarddurnam@gmail.com)
% Adriana S. Mendez Leal (asmendezleal@gmail.com)
% Carol L. Wilkinson (carol.wilkinson@childrens.harvard.edu)
% April R. Levin (april.levin@childrens.harvard.edu)
%
% HAPP-E includes code that is dependent on the following third-party software.
% Please reference this third-party software in any manuscripts making use of
HAPP-E as below:
%
% EEGLab A Delorme & S Makeig (2004) EEGLAB: an open source toolbox for
% analysis of single-trial EEG dynamics. Journal of Neuroscience Methods 134:9-
21
%
% Cleanline by Tim Mullen (as an EEGlab plug-in): Mullen, T. (2012). NITRC:
% CleanLine: Tool/Resource Info. Available online at:
http://www.nitrc.org/projects/cleanline.
%
% MARA by Irene Winkler (as an EEGlab plug-in): Winkler, et al. Automatic
% Classification of Artifactual ICA-Components for Artifact Removal in
% EEG Signals. Behavioral and Brain Functions 7:30 (2011).
%
% FASTER segment-level channel interpolation code: Nolan*, H., Whelan*, R.,
% & Reilly, R.B. (2010). FASTER: Fully Automated Statistical Thresholding
% for EEG artifact     Rejection. Journal of Neuroscience Methods, 192,152-162.
%
% I have modified Matlab central code for the wICA function originally posted by
% Jordan Sorokin (2015)
https://www.mathworks.com/matlabcentral/fileexchange/55413-wica-data-varargin-
%
% Any code that is not part of the third-party dependencies is released
% under the GNU General Public License version 3.

% This software is being distributed with the hope that it will be useful,
% but WITHOUT ANY WARRANTY; without even implied warranty of
% MERCHANTABILITY or FITNESS FOR A PARTICULAR PURPOSE. See GNU General
% Public License for more details.
%
% In no event shall Boston Childrenís Hospital (BCH), the BCH Division of
Developmental Medicine, the
% Laboratories of Cognitive Neuroscience (LCN), or pipeline contributors to
BEAPP be
% liable to any party for direct, indirect, special, incidental, or
% consequential damages, including lost profits, arising out of the use of
```

```
% this software and its documentation, even if Boston Childrenís Hospital,
% the Lab of Cognitive Neuroscience, and software contributors have been
% advised of the possibility of such damage. Software and documentation is
% provided ìas is.î Boston Childrenís Hospital, the Lab of Cognitive
% Neuroscience, and software contributors are under no obligation to
% provide maintenance, support, updates, enhancements, or modifications.
%
% This program is free software: you can redistribute it and/or modify it
% under the terms of the GNU General Public License (version 3) as
% published by the Free Software Foundation.
%~~~~~~~~~~~~~~~~~~~~~~~~~~~~~~~~~~~~~~~~~~~~~~~~~~~~~~~~~~~~~~~~~~~~~~~~~~
%%   10 USER INPUTS TO EDIT:

% 1. enter path to the folder that has the datasets you want to analyze
src_folder_name='C:\happe_testing';

% 2. Which acquisition would you like to use?
% Note: Users wishing to use a different net type can run HAPPE in
% the Batch EEG Automated Processing Platform (BEAPP),  as described in the
HAPPE manuscript
% EGI Geodesic Sensor Net 64 Channel v2.0: layout_type = 1;
% EGI Hydrocel Geodesic Sensor Net 128 v1.0 : layout_type = 2;
layout_type = 2;

% 3. list channels of interest, including the 10-20 channels. User defined
channels occur at the end of the sequence e.g. 'E39' 'E40'
%the 18 "10-20" channels that NEED to be in the chan_IDs: 'FP1' 'FP2' 'F3'
% 'F4' 'F7' 'F8' 'C3' 'C4' 'T3' 'T4' 'PZ' 'O1' 'O2' 'T5' 'T6' 'P3' 'P4' 'Fz'
chan_IDs={'FP1' 'FP2' 'F3' 'F4' 'F7' 'F8' 'C3' 'C4' 'T3' 'T4' 'PZ' 'O1' 'O2' ...
    'T5' 'T6' 'P3' 'P4' 'Fz' 'E27' 'E23' 'E19' 'E20' 'E28' 'E13' 'E41' 'E40'
'E46'...
    'E47' 'E75' 'E3' 'E4' 'E123' 'E118' 'E112' 'E117' 'E109' 'E102' 'E98'
'E103'};

% 4. run HAPPE in semi-automated setting with visualizations (=1) or fully-
automated, no visualizations setting ( = 0)
pipeline_visualizations_semiautomated = 0;
%if semi-automated, what is the minimum and maximum frequency you want to
visualize in the power spectrum figure for each file?
vis_freq_min = 2;
vis_freq_max = 57;
%if semi-automated, which frequencies do you want to generate spatial topoplots
for within the figure?
freq_to_plot = [6 10 20 30 55];

% 5. for resting-state EEG, set task_EEG_processing = 0
%for task-related EEG, set task_EEG_processing = 1
task_EEG_processing = 0;

%if task-related EEG:
task_conditions = {'near' 'devr'}; %enter the stimulus condition tags

%if resting-state EEG:
% list all potential names of the matlab variable that contains the EEG data for
your files:
```

```
% Note that variable names that include the string 'Segment' may cause
% difficulties with pop_importegimat (as data should not be pre-epoched).
% Consider renaming these variables if difficulties arise
potential_eeg_var_names = {'Category_1_Segment1','Category_1'};

% 6. do you want to segment your data? yes (=1) or no (=0)
segment_data = 1;

%if you are segmenting your task-related EEG:
%parameters to segment the data for each stimulus, in seconds:
task_segment_start = -0.5;
task_segment_end = 1.5;

%if you are segmenting your resting-state EEG:
%how long do you want your segments to be in seconds? here, 2 seconds is the
default
segment_length = 2;

% 7. do you want to interpolate the specific channels' data determined to be
artifact/bad within each segment?
%yes = 1, no = 0.
%This is segment-level channel interpolation from the FASTER EEGlab plug-in.
segment_interpolation = 1;

% 8. do you want to do segment rejection (using amplitude and joint probability
criteria)?
%yes = 1, no = 0.
segment_rejection = 0;

% if you are rejecting segments, what minimum/maximum signal amplitude do you
want to use as the artifact threshold?
reject_min_amp = -40;
reject_max_amp = 40;

% do you want to do segment rejection using all user-specified channels above (
= 0) or a subset of channels in an ROI ( = 1)?
ROI_channels_only = 0;

% if you want to do ROI segment rejection, which channels should be used in the
ROI?
% ex ROI_channels = {'E27','E20'};
ROI_channels = {'E27','E20'};

% 9. Select the type of re-referencing you want. Average re-reference (=1)
% or re-referencing to another channel/subset of channels (=0)
average_rereference = 1;

% if you are referencing to another channel/subset of channels, what are they?
% make sure to use the channel name given above
% ex ROI_channels = {'E57','E100'};
NO_AVERAGE_REREF_channel_subset = {'E57','E100'};

% 10. Select the format to save your processed data at the end of HAPPE!
%save_as_format = 2 will save the processed data as a .mat file (matlab format)
```

```
%save_as_format = 1 will save the processed data as a .txt file.(electrodes as
columns, time as rows)
%save_as_format = 0 will save the processed data as a .set file (EEGlab format)
save_as_format = 0;

%~~~~~~~~~~~~~~~~~~~~~~ no need to edit beyond this point ~~~~~~~~~~~~~~~~~~
%~~~~~~~~~~~~~~~~~~~~~~~~~~~~~~~~~~~~~~~~~~~~~~~~~~~~~~~~~~~~~~~~~~~~~~~~~~
%% make output folders:

%make folder for intermediate waveleted files
if ~isdir ([src_folder_name filesep 'intermediate1_wavclean'])
    mkdir ([src_folder_name filesep 'intermediate1_wavclean']);
end

%make folder for post-ICA, uninterpolated files
if ~isdir ([src_folder_name filesep 'intermediate2_ICAclean'])
    mkdir ([src_folder_name filesep 'intermediate2_ICAclean']);
end

%make folder for segment-level files (if user selects segmentation option)
if ~isdir ([src_folder_name filesep 'intermediate3_segmented']) && segment_data
==1
    mkdir ([src_folder_name filesep 'intermediate3_segmented']);
end
%make folder for final preprocessed files
if ~isdir ([src_folder_name filesep 'processed'])
    mkdir ([src_folder_name filesep 'processed']);
end

%% go to the folder with data, pull file names to feed script,and initialize the
arrays to store file specific data quality metrics

% add HAPPE scripts path
happe_directory_path = fileparts(which('HAPPE_pipeline_v1_0.m'));
addpath(genpath(happe_directory_path));

% add cleanline path
if exist('cleanline','file')
    cleanline_path = which('eegplugin_cleanline.m');
    cleanline_path =
cleanline_path(1:findstr(cleanline_path,'eegplugin_cleanline.m')-1);
    addpath(genpath(cleanline_path));
else
    error('please make sure cleanline is on your path');
end

% find path to sensor layout
if layout_type ==1
    chan_locations = [happe_directory_path filesep
'acquisition_layout_information' filesep 'GSN64v2_0.sfp'];
elseif layout_type ==2
    chan_locations = [happe_directory_path filesep
'acquisition_layout_information' filesep 'GSN-HydroCel-128.sfp'];
else
    error ('Invalid sensor layout selection')
```

```
end

% cd get file list
cd (src_folder_name);
if task_EEG_processing == 1
    src_file_ext = '.raw';
else
    src_file_ext = '.mat';
end

FileNames=dir(['*' src_file_ext]);
FileNames={FileNames.name};

% intialize report metrics
chan_index=[1:length(chan_IDs)];
Number_ICs_Rejected=[];
Number_Good_Channels_Selected=[];
Interpolated_Channel_IDs=[];
Percent_ICs_Rejected=[];
Percent_Variance_Kept_of_Post_Waveleted_Data=[];
File_Length_In_Secs=[];
Number_Channels_User_Selected=[];
Percent_Good_Channels_Selected=[];
Median_Artifact_Probability_of_Kept_ICs=[];
Mean_Artifact_Probability_of_Kept_ICs=[];
Range_Artifact_Probability_of_Kept_ICs=[];
Min_Artifact_Probability_of_Kept_ICs=[];
Max_Artifact_Probability_of_Kept_ICs=[];
Number_Segments_Post_Segment_Rejection=[];

%iterate the following preprocessing pipeline over all your data files:
for current_file = 1:length(FileNames)

    cd (src_folder_name);

    %% load file and get sampling rate, save with double precision

    % import data into eeglab and store the file's length in seconds for
outputting later
    if task_EEG_processing == 1
        EEGloaded = pop_readegi(FileNames{current_file}, [],[],'auto');
        events=EEGloaded.event;
        complete_event_info=EEGloaded.urevent;
        srate=double(EEGloaded.srate);
    end

    if task_EEG_processing == 0
        load(FileNames{current_file});
        srate=double(samplingRate);
        file_eeg_vname = intersect(who,potential_eeg_var_names);

        try
            EEGloaded = pop_importegimat(FileNames{current_file}, srate, 0.00,
file_eeg_vname{1});
        catch err_msg
```

```
            if strcmp(err_msg.identifier,'MATLAB:badsubscript')
                error('sorry, could not read the variable name of the EEG data,
please check your file')
            else
                error(err_msg.message);
            end
        end
    end

    EEGloaded.setname='rawEEG';
    EEG = eeg_checkset(EEGloaded);
    File_Length_In_Secs(current_file)=EEG.xmax;

    % edit channel locations (does not import properly from netstation by
default)
    EEG=pop_chanedit(EEG, 'load',{chan_locations 'filetype' 'autodetect'});
    EEG = eeg_checkset( EEG );

    %load 10-20 EEG system labels for electrode names (for MARA to reference)
    load('happe_netdata_lib.mat')
    %for 128 channel nets:
    if EEG.nbchan >65
        for i=1:length(netdata_lib.net128.lead_nums_sub)
            EEG=pop_chanedit(EEG,
'changefield',{netdata_lib.net128.lead_nums_sub(i)  'labels'
netdata_lib.net128.lead_list_sub{i}});
        end
        %for 64 channel nets
    elseif EEG.nbchan > 50
        for i=1:length(netdata_lib.net64.lead_nums_sub)
            EEG=pop_chanedit(EEG,
'changefield',{netdata_lib.net64.lead_nums_sub(i)  'labels'
netdata_lib.net64.lead_list_sub{i}});
        end
    end
    EEG = eeg_checkset( EEG );

    %% filter the data with 1hz highpass (for srate 250), bandpass 1hz-249hz
(for srate 500, ICA doesn't reliably work well with frequencies above 250hz)
    if srate<500
        EEG = pop_eegfiltnew(EEG, [],1,[],1,[],0);
    elseif srate >= 500
        EEG = pop_eegfiltnew(EEG, 1,249,[],0,[],0);
    end
    EEG.setname='rawEEG_f';
    EEG = eeg_checkset( EEG );

    %% select EEG channels of interest for analyses and 10-20 channels from the
list you specified at the top of the script

    EEG = pop_select( EEG,'channel', chan_IDs);
    EEG.setname='rawEEG_f_cs';
    EEG = eeg_checkset( EEG );
    full_selected_channels = EEG.chanlocs;
```

```
    %% reduce line noise in the data (note: may not completely eliminate, re-
referencing helps at the end as well)
    EEG = pop_cleanline(EEG,
'bandwidth',2,'chanlist',chan_index,'computepower',1,'linefreqs',[60 120]
,'normSpectrum',0,'p',0.01,'pad',2,'plotfigures',0,'scanforlines',1,'sigtype','C
hannels','tau',100,'verb',0,'winsize',4,'winstep',1,
'ComputeSpectralPower','False');
    EEG.setname='rawEEG_f_cs_ln';
    EEG = eeg_checkset( EEG );

    % close window if visualizations are turned off
    if pipeline_visualizations_semiautomated == 0
        close all;
    end
    %% crude bad channel detection using spectrum criteria and 3SDeviations as
channel outlier threshold, done twice
    EEG = pop_rejchan(EEG, 'elec',chan_index,'threshold',[-3
3],'norm','on','measure','spec','freqrange',[1 125] );
    EEG.setname='rawEEG_f_cs_ln_badc';
    EEG = eeg_checkset( EEG );

    EEG = pop_rejchan(EEG, 'elec',[1:EEG.nbchan],'threshold',[-3
3],'norm','on','measure','spec','freqrange',[1 125] );
    EEG.setname='rawEEG_f_cs_ln_badc2x';
    EEG = eeg_checkset( EEG );
    selected_channel_locations=EEG.chanlocs;

    %save the names of the rejected channels for output table after the pipeline
finishes
    selected_channel_labels={selected_channel_locations.labels};
    bad_channels_removed= setdiff(chan_IDs, selected_channel_labels);
    [~,ROI_indices_in_selected_chanlocs] =
intersect(selected_channel_labels,ROI_channels);

    %% run wavelet-ICA (ICA first for clustering the data, then wavelet
thresholding on the ICs)
    %uses a soft, global threshold for the wavelets, wavelet family is coiflet
(level 5), threshold multiplier .75 to remove more high frequency noise
    %for details, see wICA.m function
    if pipeline_visualizations_semiautomated == 0
        [wIC, A, W, IC] = wICA(EEG,'runica', 1, 0, [], 5);
    elseif pipeline_visualizations_semiautomated == 1
        [wIC, A, W, IC] = wICA(EEG,'runica', 1, 1, srate, 5);
    end

    %reconstruct artifact signal as channelsxsamples format from the wavelet
coefficients
    artifacts = A*wIC;

    %reshape EEG signal from EEGlab format to channelsxsamples format
    EEG2D=reshape(EEG.data, size(EEG.data,1), []);

    %subtract out wavelet artifact signal from EEG signal
    wavcleanEEG=EEG2D-artifacts;
```

```
    %save wavelet cleaned EEG data file to folder with extension _wavclean.mat
    cd ([src_folder_name filesep 'intermediate1_wavclean']);

    save(strrep(FileNames{current_file},
src_file_ext,'_wavclean.mat'),'wavcleanEEG')
    save(strrep(FileNames{current_file}, src_file_ext,'_prewav.mat'),'EEG2D')

    %% reimport into EEGlab
    EEG =
pop_importdata('dataformat','matlab','nbchan',0,'data',strrep(FileNames{current
_file},src_file_ext,'_wavclean.mat'),'srate',srate,'pnts',0,'xmin',0,'chanlocs',
selected_channel_locations);
    EEG.setname='wavcleanedEEG';
    EEG = eeg_checkset( EEG );

    % import event tags if needed
    if task_EEG_processing == 1
        EEG.event=events;
        EEG.urevent=complete_event_info;
    end

    %% run ICA to evaluate components this time
    EEG = pop_runica(EEG, 'extended',1,'interupt','on');
    EEG = eeg_checkset( EEG );

    %save the ICA decomposition intermediate file before cleaning with MARA
    EEG = pop_saveset(EEG, 'filename',strrep(FileNames{current_file},
src_file_ext,'_ICA.set'),'filepath',[src_folder_name filesep
'intermediate2_ICAclean']);

    %% use MARA to flag artifactual IComponents automatically if artifact
probability > .5
    [~,EEG,~]=processMARA ( EEG,EEG,EEG, [0, 0,
pipeline_visualizations_semiautomated,...
        pipeline_visualizations_semiautomated ,
pipeline_visualizations_semiautomated] );

    EEG.reject.gcompreject = zeros(size(EEG.reject.gcompreject));
    EEG.reject.gcompreject(EEG.reject.MARAinfo.posterior_artefactprob > 0.5) =
1;
    EEG.setname='wavcleanedEEG_ICA_MARA';
    EEG = eeg_checkset( EEG );

    % store MARA related variables to assess ICA/data quality
    index_ICs_kept=(EEG.reject.MARAinfo.posterior_artefactprob < 0.5);
    median_artif_prob_good_ICs =
median(EEG.reject.MARAinfo.posterior_artefactprob(index_ICs_kept));
    mean_artif_prob_good_ICs =
mean(EEG.reject.MARAinfo.posterior_artefactprob(index_ICs_kept));
    range_artif_prob_good_ICs =
range(EEG.reject.MARAinfo.posterior_artefactprob(index_ICs_kept));
    min_artif_prob_good_ICs =
min(EEG.reject.MARAinfo.posterior_artefactprob(index_ICs_kept));
    max_artif_prob_good_ICs =
max(EEG.reject.MARAinfo.posterior_artefactprob(index_ICs_kept));
```

```
    %store IC variables and calculate variance of data that will be kept after
IC rejection:
    ICs_to_keep =find(EEG.reject.gcompreject == 0);
    ICA_act = EEG.icaact;
    ICA_winv =EEG.icawinv;

    %variance of wavelet-cleaned data to be kept = varianceWav:
    [projWav, varianceWav] =compvar(EEG.data, ICA_act, ICA_winv, ICs_to_keep);

    %% reject the ICs that MARA flagged as artifact
    artifact_ICs=find(EEG.reject.gcompreject == 1);
    EEG = pop_subcomp( EEG, artifact_ICs, 0);
    EEG.setname='wavcleanedEEG_ICA_MARA_rej';
    EEG = eeg_checkset( EEG );

    %% save the post-MARA cleaned intermediate file before interpolating
anything
    EEG = pop_saveset(EEG, 'filename',strrep(FileNames{current_file},
src_file_ext,'_ICAcleanedwithMARA.set'),'filepath',[src_folder_name filesep
'intermediate2_ICAclean']);

    %% segment data according to data type
    if segment_data
        if ~task_EEG_processing
            EEG=eeg_regepochs(EEG,'recurrence',segment_length,'limits',[0
segment_length], 'rmbase', [NaN]);
        else
            EEG = pop_epoch(EEG, task_conditions, [task_segment_start
task_segment_end], 'verbose', 'no', 'epochinfo', 'yes');
        end
    end

    EEG = pop_saveset( EEG,
'filename',strrep(FileNames{current_file},src_file_ext,'_segmented.set'),'filepa
th',[src_folder_name filesep 'intermediate3_segmented']);
    %% if selected option, interpolate bad data within segments from "good
channels" only:
    if segment_interpolation

        %use only the good channels to evaluate data:
        eeg_chans=[1:length(selected_channel_locations)];

        %evaluate the channels for each segment and interpolate channels with
bad
        %data for that each segment using the FASTER program, interpolating
channels scoring above/below z threshold of 3 for an segment:
        ext_chans=[];
        o.epoch_interp_options.rejection_options.measure = [1 1 1 1];
        o.epoch_interp_options.rejection_options.z = [3 3 3 3];

        if  length(size(EEG.data)) > 2
            status = '';
            lengths_ep=cell(1,size(EEG.data,3));
            for v=1:size(EEG.data,3)
```

```
                list_properties =
single_epoch_channel_properties(EEG,v,eeg_chans);

lengths_ep{v}=eeg_chans(logical(min_z(list_properties,o.epoch_interp_options.rej
ection_options)));
                status = [status sprintf('%d: ',v) sprintf('%d ',lengths_ep{v})
sprintf('\n')];
            end
            EEG=h_epoch_interp_spl(EEG,lengths_ep,ext_chans);
            EEG.saved='no';

            %add the info about which channels were interpolated for each
segment to the EEG file
            EEG.etc.epoch_interp_info=[status];
        end

        EEG = pop_saveset(EEG, 'filename',strrep(FileNames{current_file},
src_file_ext,'_segments_interp.set'),'filepath',[src_folder_name filesep
'intermediate3_segmented']);
    end

    %% rejection of bad segments using amplitude-based and joint probability
artifact detection
    if ROI_channels_only == 0
        EEG = pop_eegthresh(EEG,1,[1:EEG.nbchan]
,[reject_min_amp],[reject_max_amp],[EEG.xmin],[EEG.xmax],2,0);
        EEG =
pop_jointprob(EEG,1,[1:EEG.nbchan],3,3,pipeline_visualizations_semiautomated,...

0,pipeline_visualizations_semiautomated,[],pipeline_visualizations_semiautomate
d);
    else
        EEG =
pop_eegthresh(EEG,1,[ROI_indices_in_selected_chanlocs]',[reject_min_amp],[reject
_max_amp],[EEG.xmin],[EEG.xmax],2,0);
        EEG =
pop_jointprob(EEG,1,[ROI_indices_in_selected_chanlocs]',3,3,pipeline_visualizati
ons_semiautomated,...

0,pipeline_visualizations_semiautomated,[],pipeline_visualizations_semiautomate
d);
    end

    EEG = eeg_rejsuperpose(EEG, 1, 0, 1, 1, 1, 1, 1, 1);
    EEG = pop_rejepoch(EEG, [EEG.reject.rejglobal] ,0);
    EEG = eeg_checkset(EEG );
    EEG = pop_saveset(EEG, 'filename',strrep(FileNames{current_file},
src_file_ext,'_segments_postreject.set'),'filepath',[src_folder_name filesep
'intermediate3_segmented']);

    %% interpolate the channels that were flagged as bad earlier:
    EEG = pop_interp(EEG, full_selected_channels, 'spherical');
    EEG.setname='wavcleanedEEG_ICA_MARA_rej_chan_int';
    EEG = eeg_checkset(EEG );
```

```
    %% re-reference the data: average reference used here

    if average_rereference == 1;
        EEG = pop_reref(EEG, []);
        EEG.setname='wavcleanedEEG_ICA_MARA_rej_chan_int_avgreref';
        EEG = eeg_checkset(EEG);
    else
        [~,ref_chan_indices_in_full_selected_chanlocs] =
intersect({full_selected_channels.labels},NO_AVERAGE_REREF_channel_subset);
        EEG = pop_reref(EEG, ref_chan_indices_in_full_selected_chanlocs);
        EEG.setname='wavcleanedEEG_ICA_MARA_rej_chan_int_chansubsetreref';
        EEG = eeg_checkset(EEG);
    end

    %% store outputs and report metrics
    Number_Channels_User_Selected(current_file)=size(chan_IDs,2);
    Number_ICs_Rejected(current_file)=length(artifact_ICs);

Number_Good_Channels_Selected(current_file)=size(selected_channel_locations,2);

Percent_Good_Channels_Selected(current_file)=Number_Good_Channels_Selected(curre
nt_file)/Number_Channels_User_Selected(current_file)* 100;

Percent_ICs_Rejected(current_file)=Number_ICs_Rejected(current_file)/Number_Good
_Channels_Selected(current_file)* 100;
    Percent_Variance_Kept_of_Post_Waveleted_Data(current_file)=varianceWav;
    if isempty(bad_channels_removed)
        Interpolated_Channel_IDs{current_file} = 'none';
    else
        Interpolated_Channel_IDs{current_file}=[sprintf('%s
',bad_channels_removed{1:end-1}),bad_channels_removed{end}];
    end

Median_Artifact_Probability_of_Kept_ICs(current_file)=median_artif_prob_good_ICs
;

Mean_Artifact_Probability_of_Kept_ICs(current_file)=mean_artif_prob_good_ICs;

Range_Artifact_Probability_of_Kept_ICs(current_file)=range_artif_prob_good_ICs;
    Min_Artifact_Probability_of_Kept_ICs(current_file)=min_artif_prob_good_ICs;
    Max_Artifact_Probability_of_Kept_ICs(current_file)=max_artif_prob_good_ICs;
    Number_Segments_Post_Segment_Rejection(current_file)=EEG.trials;
    cd ([src_folder_name filesep 'processed']);
    %% save preprocessed dataset with subject ID as either txt file (user
specified) or eeglab .set file

    switch save_as_format
        case 1 % txt file
             pop_export(EEG,strrep(FileNames{current_file},
src_file_ext,'_processed.txt'),'transpose','on','precision',8);
        case 2 % .mat file
             save(strrep(FileNames{current_file},
src_file_ext,'_processed.mat'), 'EEG');
        case 3 % .set file
```

```
            EEG = pop_saveset(EEG, 'filename',strrep(FileNames{current_file},
src_file_ext,'_processed.set'),'filepath',[src_folder_name filesep
'processed']);
    end

    %% generate power spectrum and topoplot visualization if user requested:
    %plot the spectrum across channels to evaluate pipeline performance
    if pipeline_visualizations_semiautomated == 1
        figure; pop_spectopo(EEG, 1, [], 'EEG' , 'freq', [[freq_to_plot]],
'freqrange',[[vis_freq_min] [vis_freq_max]],'electrodes','off');
        saveas (gcf,strrep(FileNames{current_file},
src_file_ext,'_processedspectrum.jpg'));
    end
end

%% generate output table in the "preprocessed" subfolder listing the subject
file name and relevant variables for assesssing how good/bad that datafile was
and how well the pipeline worked
outputtable=table(FileNames',File_Length_In_Secs',Number_Channels_User_Selected'
,Number_Segments_Post_Segment_Rejection',...
    Number_Good_Channels_Selected', Percent_Good_Channels_Selected',
Interpolated_Channel_IDs',Number_ICs_Rejected',...
    Percent_ICs_Rejected',
Percent_Variance_Kept_of_Post_Waveleted_Data',Median_Artifact_Probability_of_Kep
t_ICs',...

Mean_Artifact_Probability_of_Kept_ICs',Range_Artifact_Probability_of_Kept_ICs',M
in_Artifact_Probability_of_Kept_ICs',...
    Max_Artifact_Probability_of_Kept_ICs');
outputtable.Properties.VariableNames
={'FileNames','File_Length_In_Secs','Number_Channels_User_Selected','Number_Seg
ments_Post_Segment_Rejection',...
    'Number_Good_Channels_Selected', 'Percent_Good_Channels_Selected',
'Interpolated_Channel_IDs','Number_ICs_Rejected',...
    'Percent_ICs_Rejected',
'Percent_Variance_Kept_of_Post_Waveleted_Data','Median_Artifact_Probability_of_K
ept_ICs',...

'Mean_Artifact_Probability_of_Kept_ICs','Range_Artifact_Probability_of_Kept_ICs'
,'Min_Artifact_Probability_of_Kept_ICs',...
    'Max_Artifact_Probability_of_Kept_ICs'};
writetable(outputtable, ['HAPPE_all_subs_output_table ',datestr(now,'dd-mm-
yyyy'),'.csv']);

rmpath(genpath(cleanline_path));
```
