# Supplementary material for: The Harvard Automated Processing Pipeline for Electroencephalography (HAPPE): Standardized Processing Software for Developmental and High-Artifact Data
Source: Front Neurosci. 2018 Feb 27;12:97. doi: 10.3389/fnins.2018.00097 (PMC5835235; doi:10.3389/fnins.2018.00097)

baselineEEG01 HAPPE visualizations:

W-ICA visual:

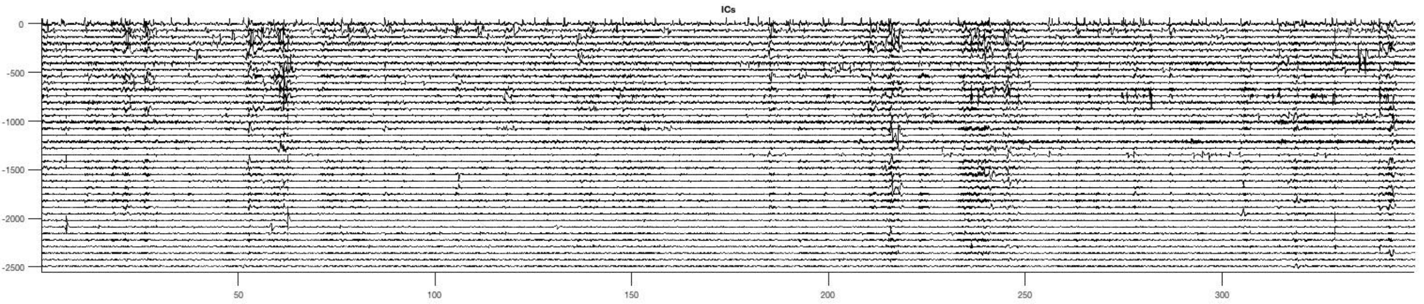

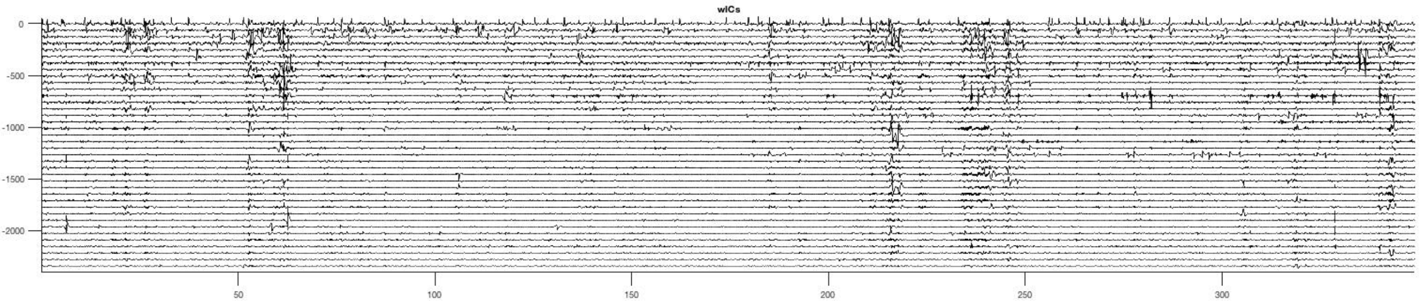

MARA visuals:

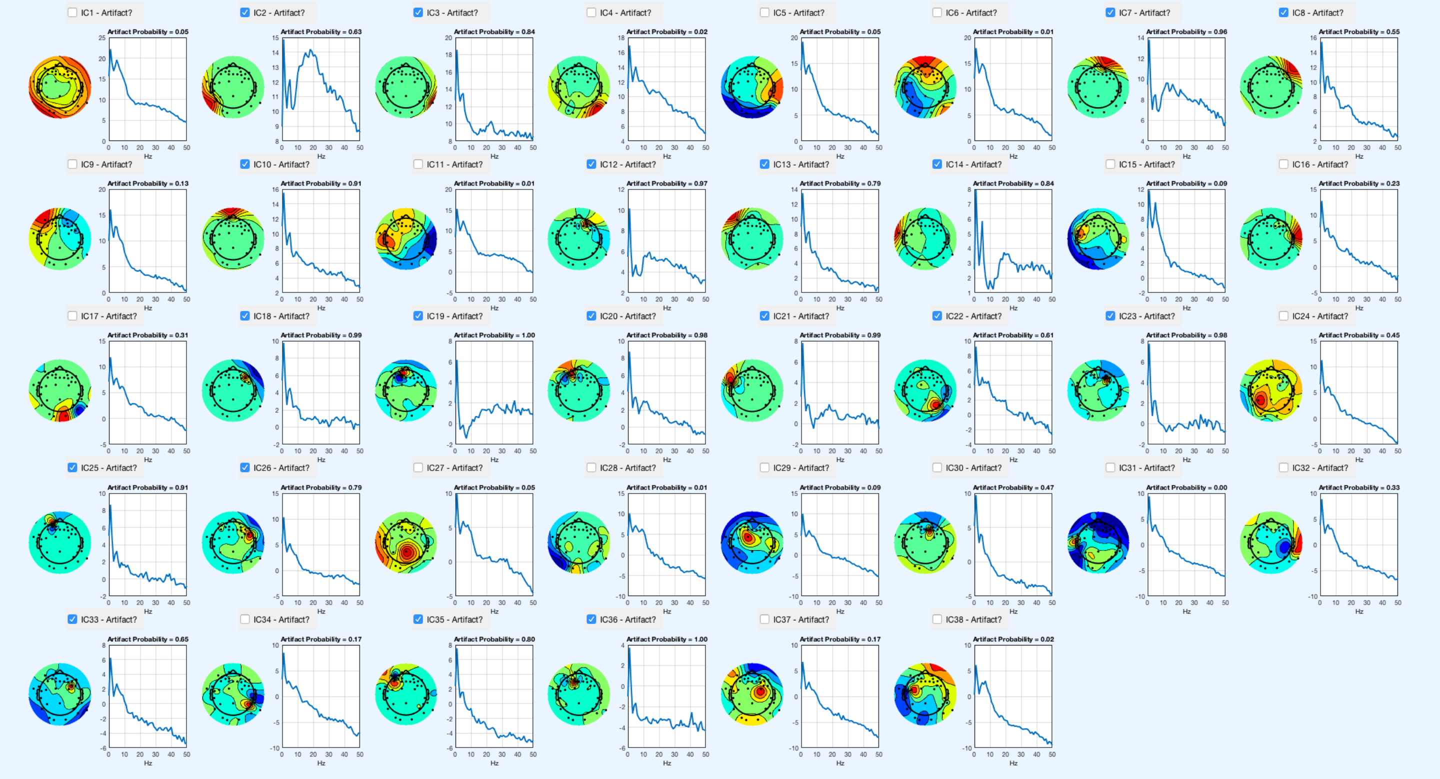

baselineEEG01 HAPPE visualizations continued

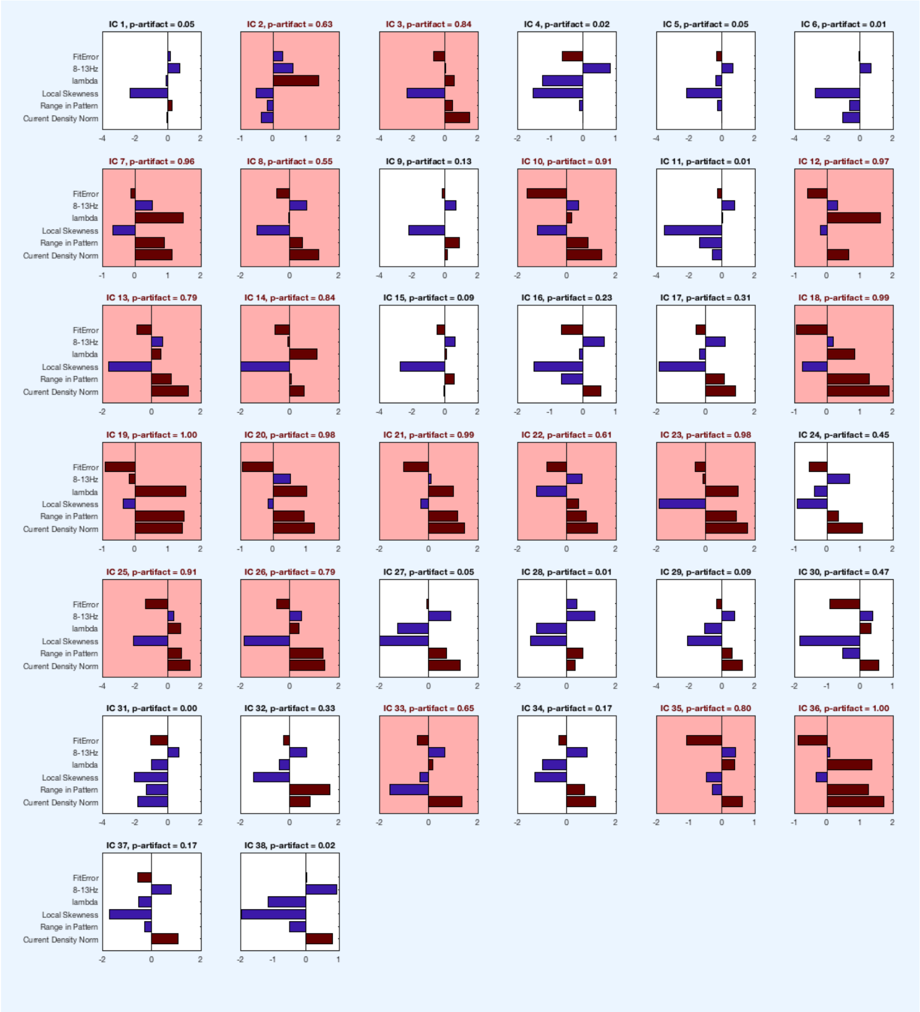

Post-processed power spectrum:

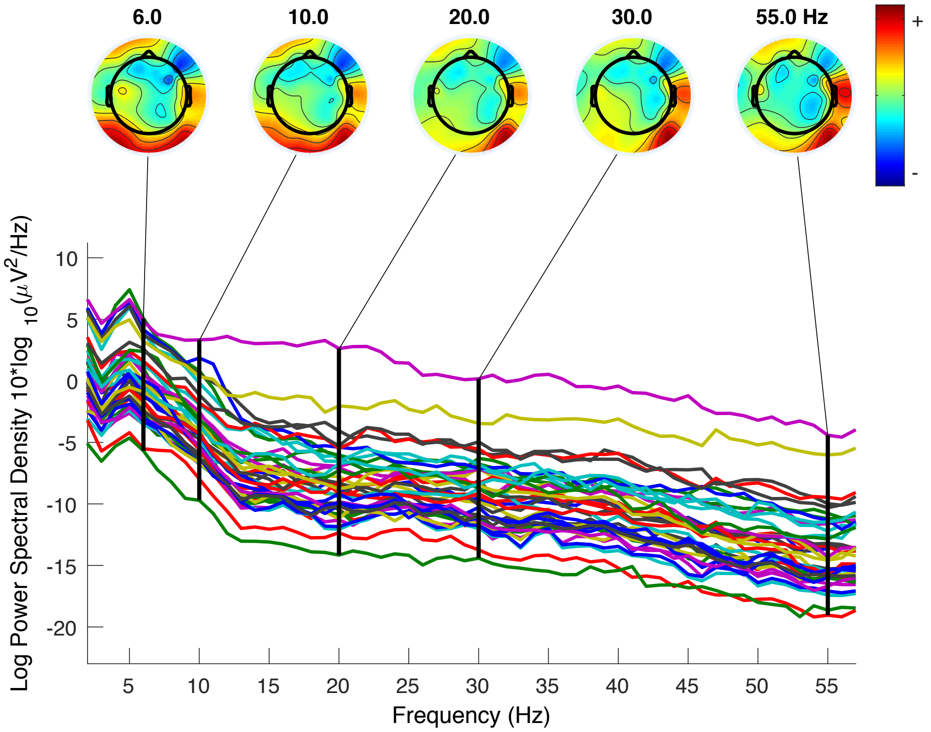

baselineEEG04 HAPPE visualizations:

W-ICA visual:

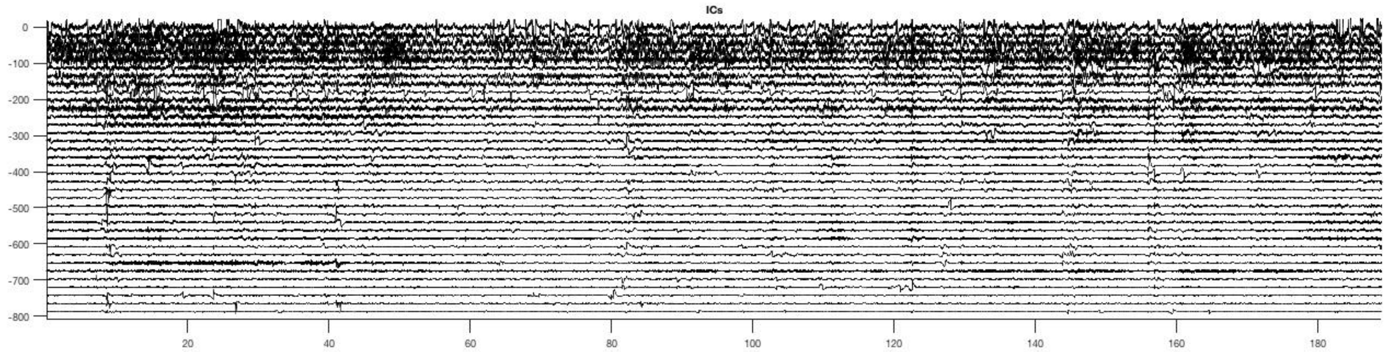

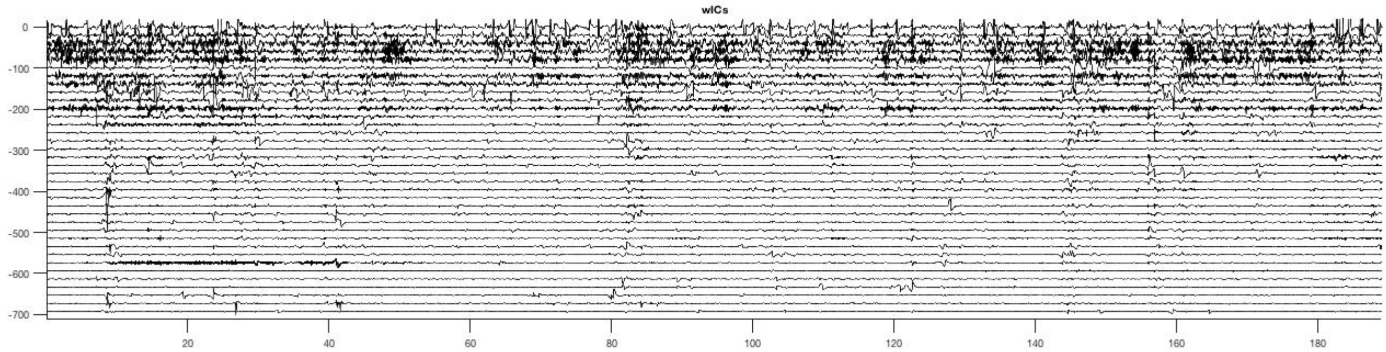

MARA visuals:

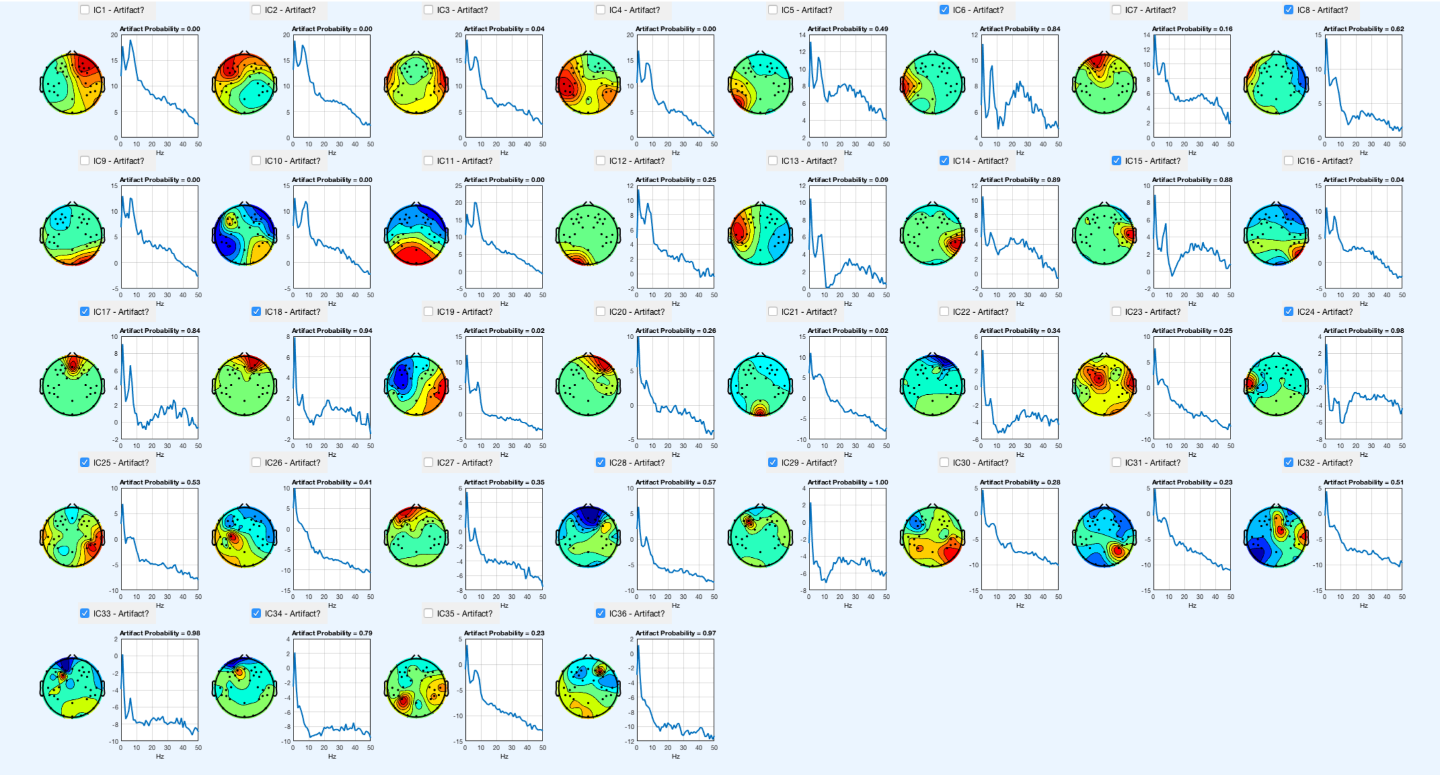

baselineEEG04 HAPPE visualizations continued

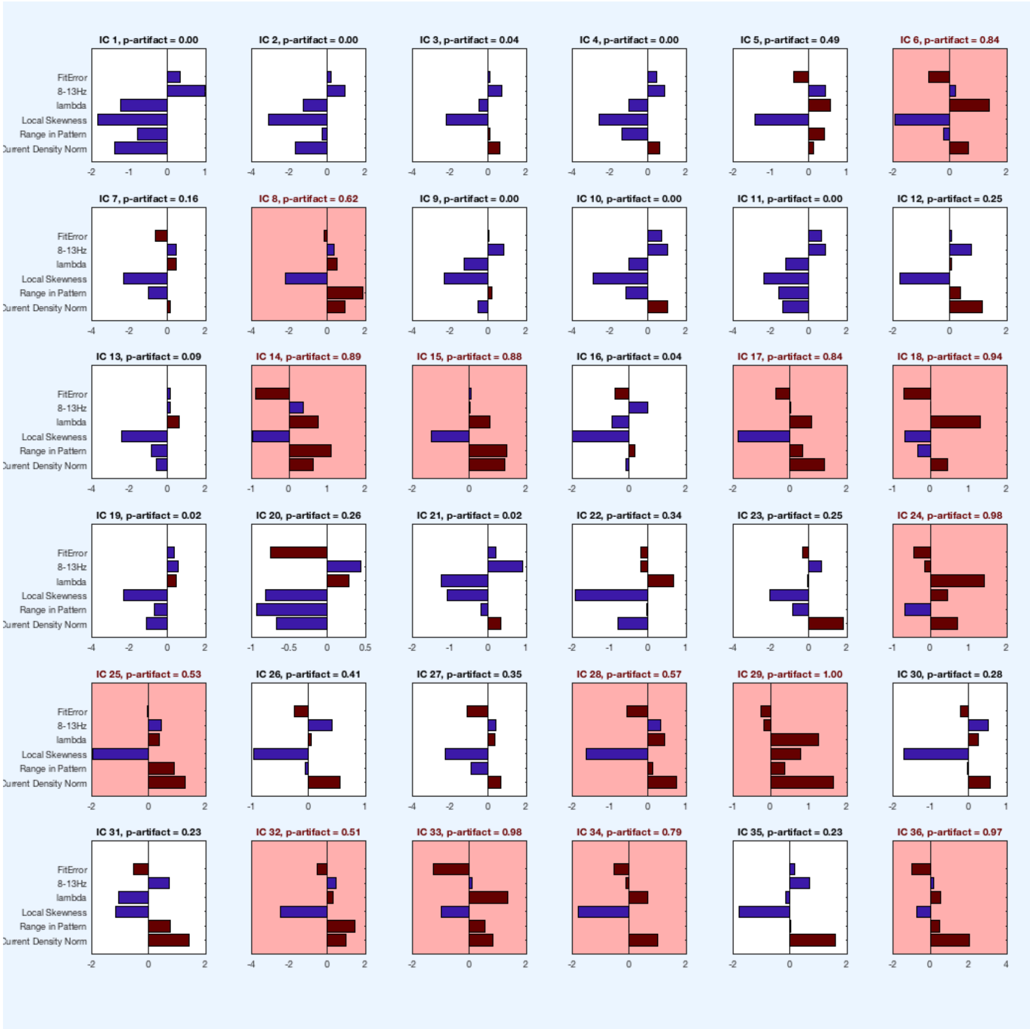

Post-processed power spectrum:

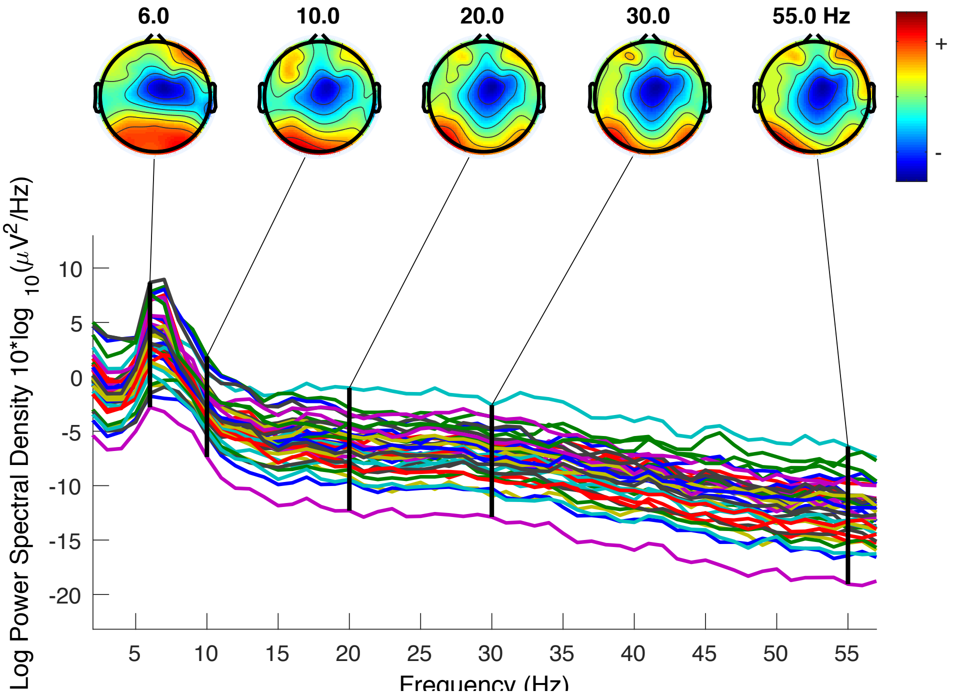

baselineEEG05 HAPPE visualizations:

W-ICA visual:

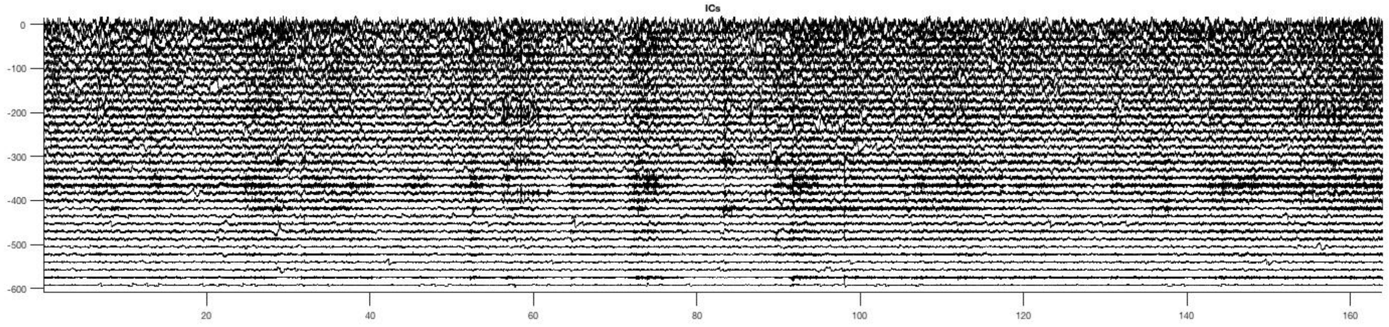

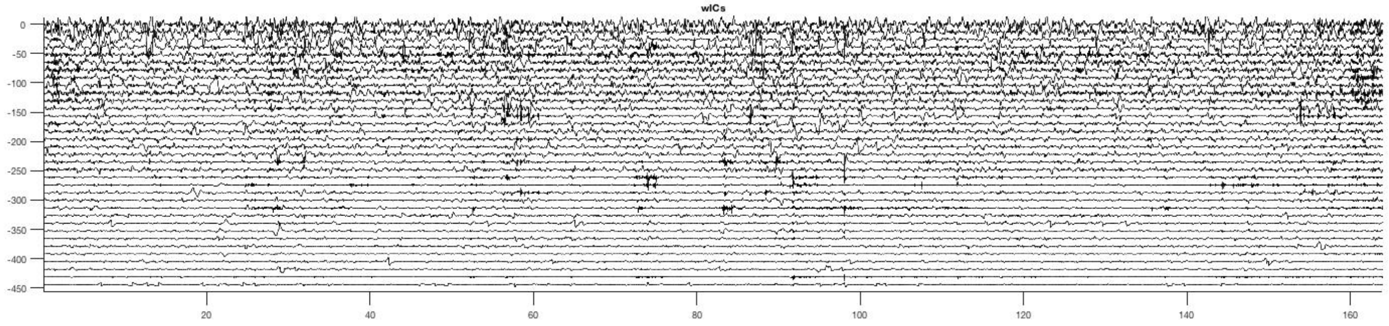

MARA visuals:

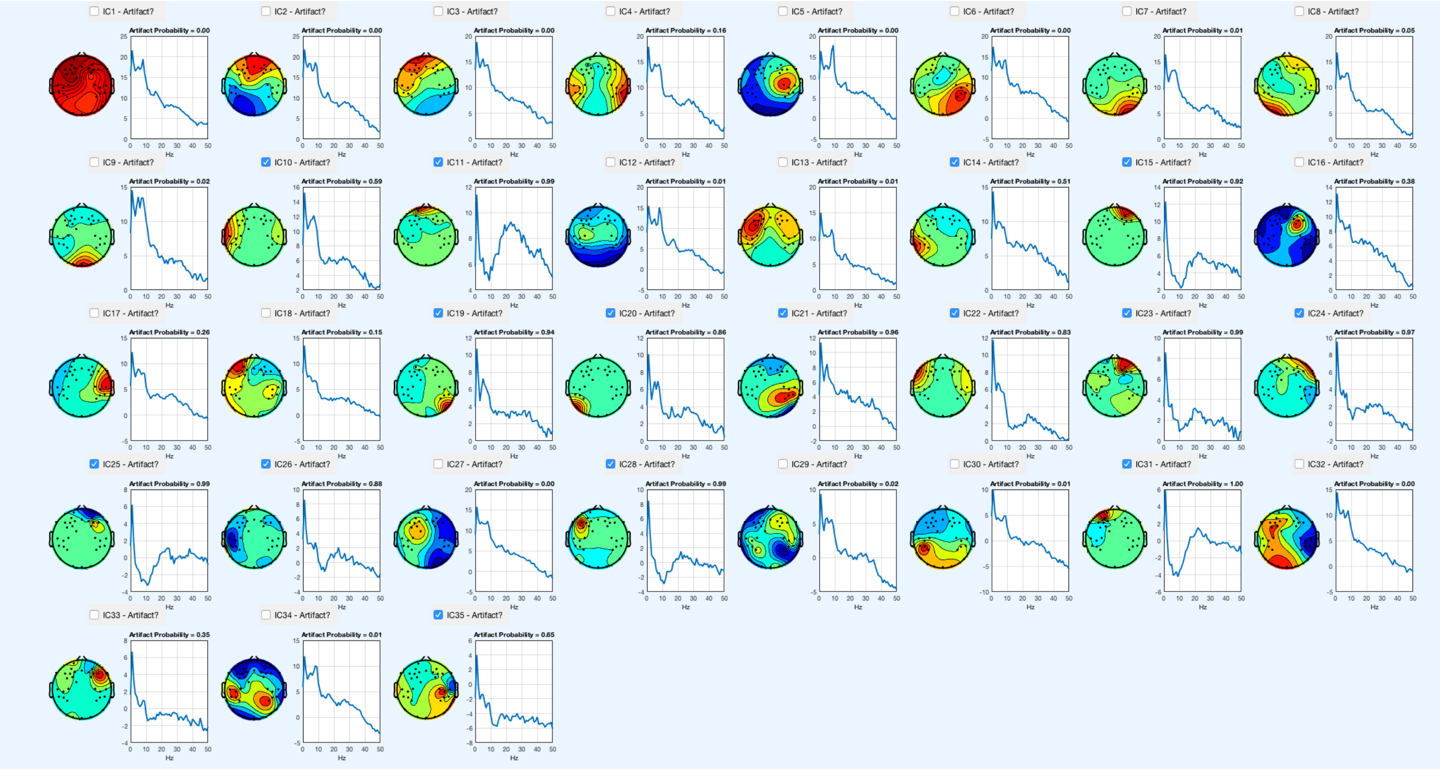

baselineEEG05 HAPPE visualizations continued

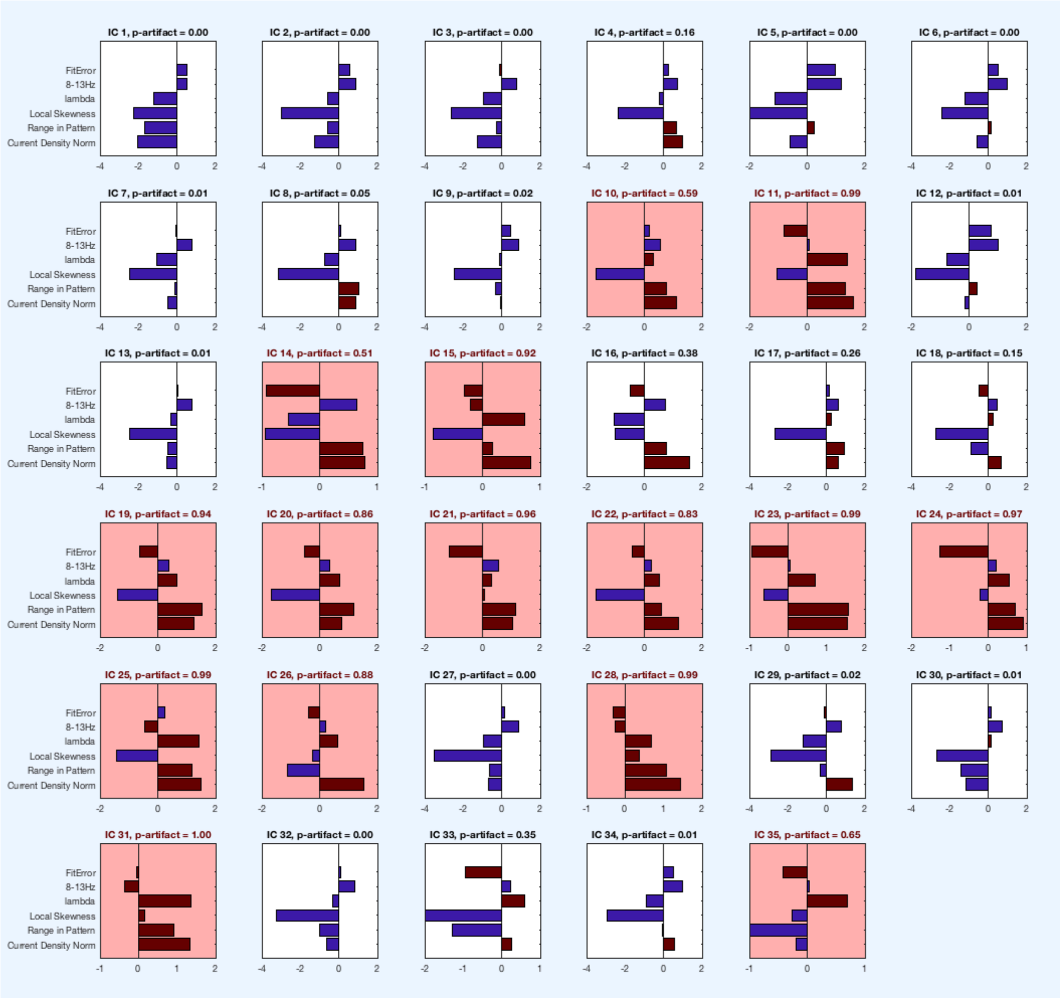

Post-processed power spectrum:

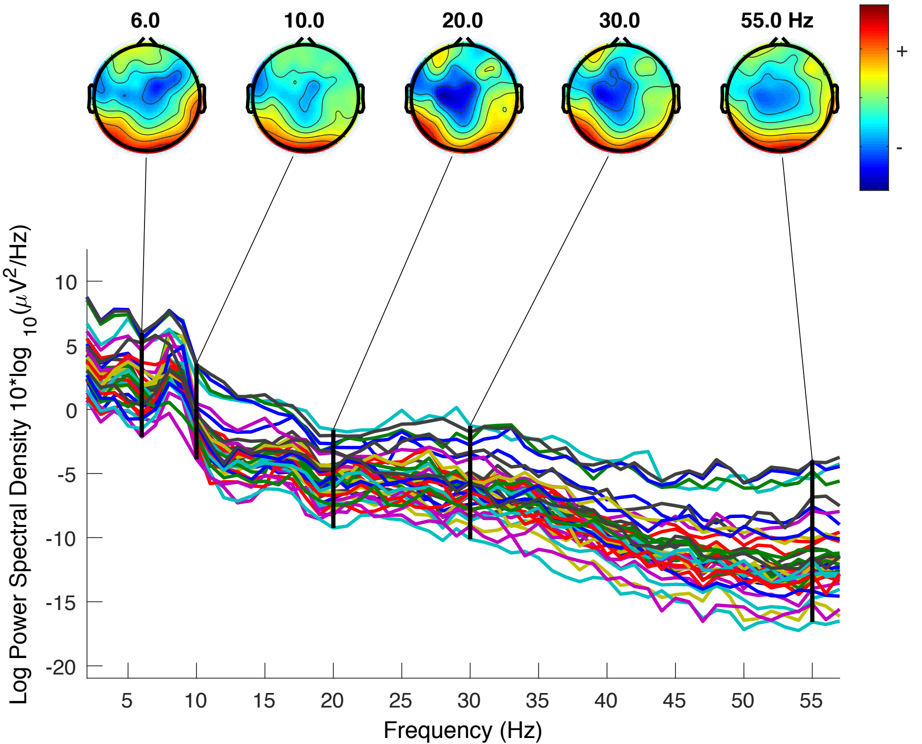

baselineEEG06 HAPPE visualizations:

W-ICA visual:

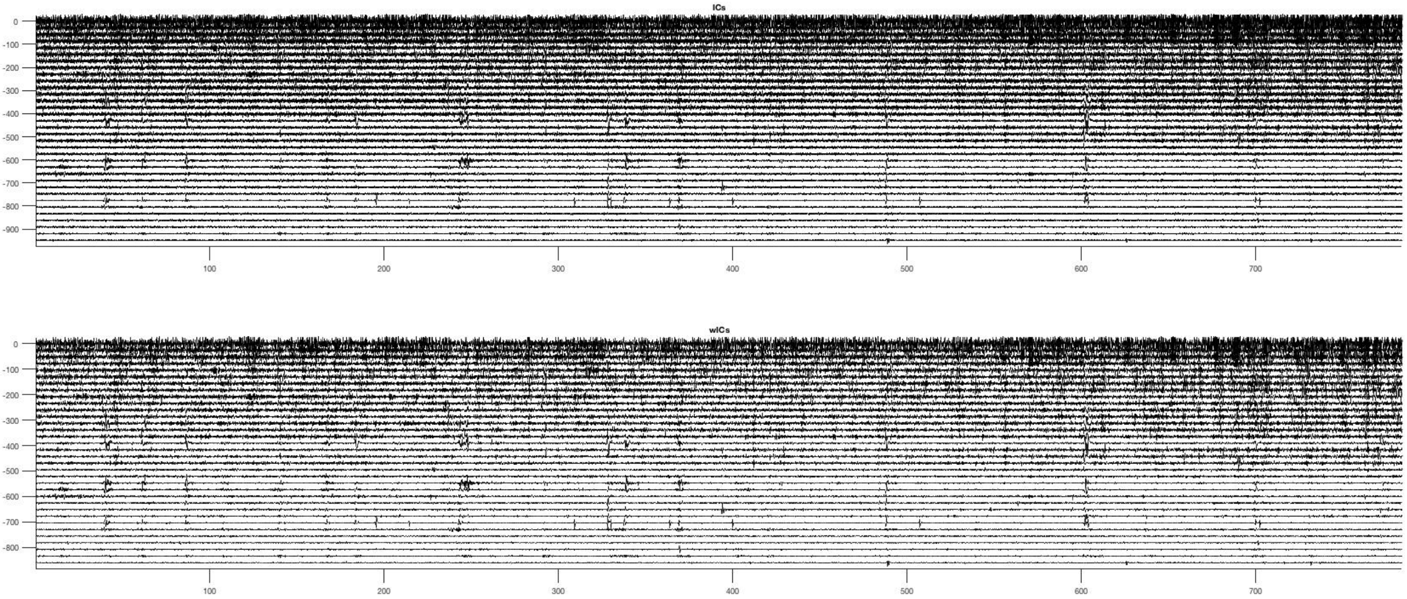

MARA visuals:

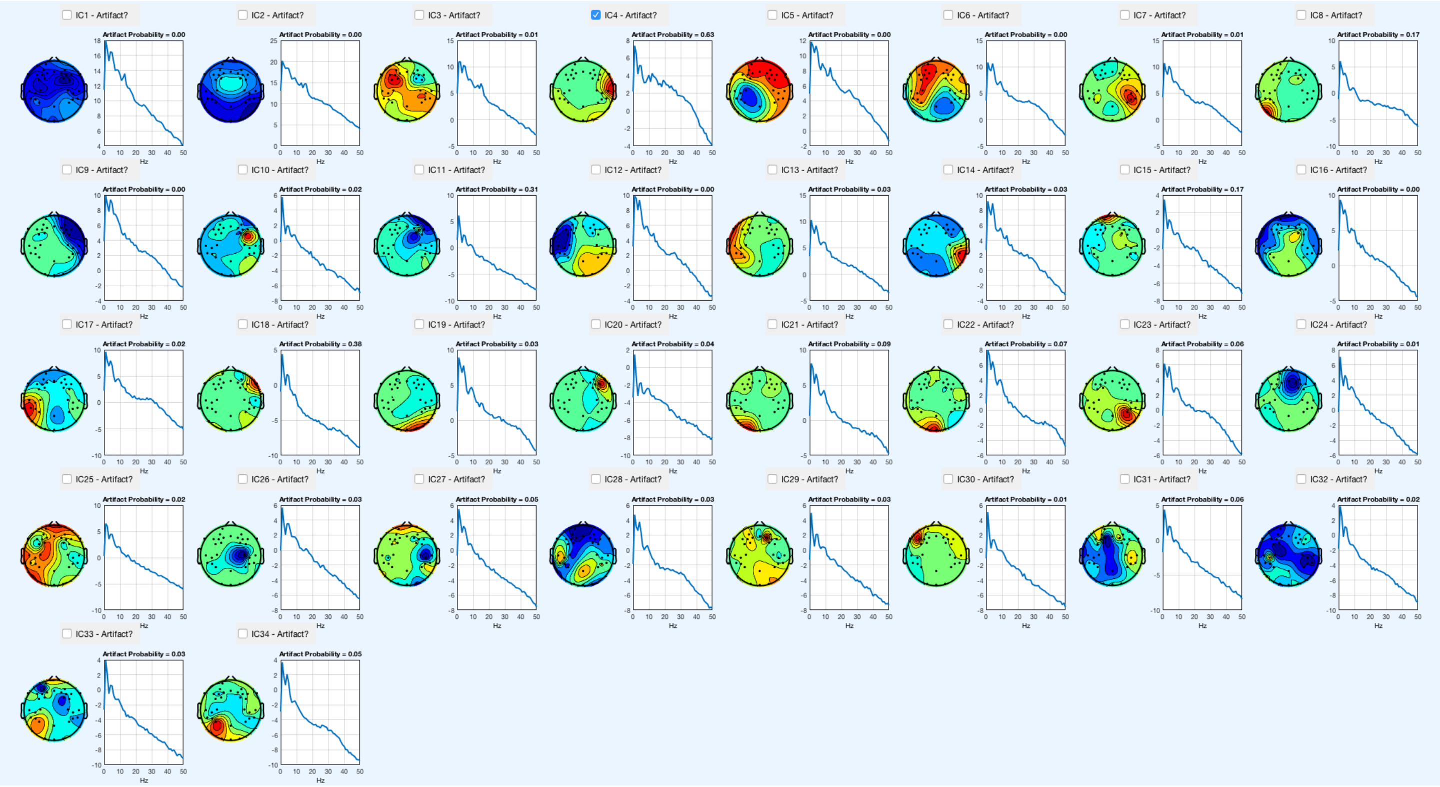

baselineEEG06 HAPPE visualizations continued

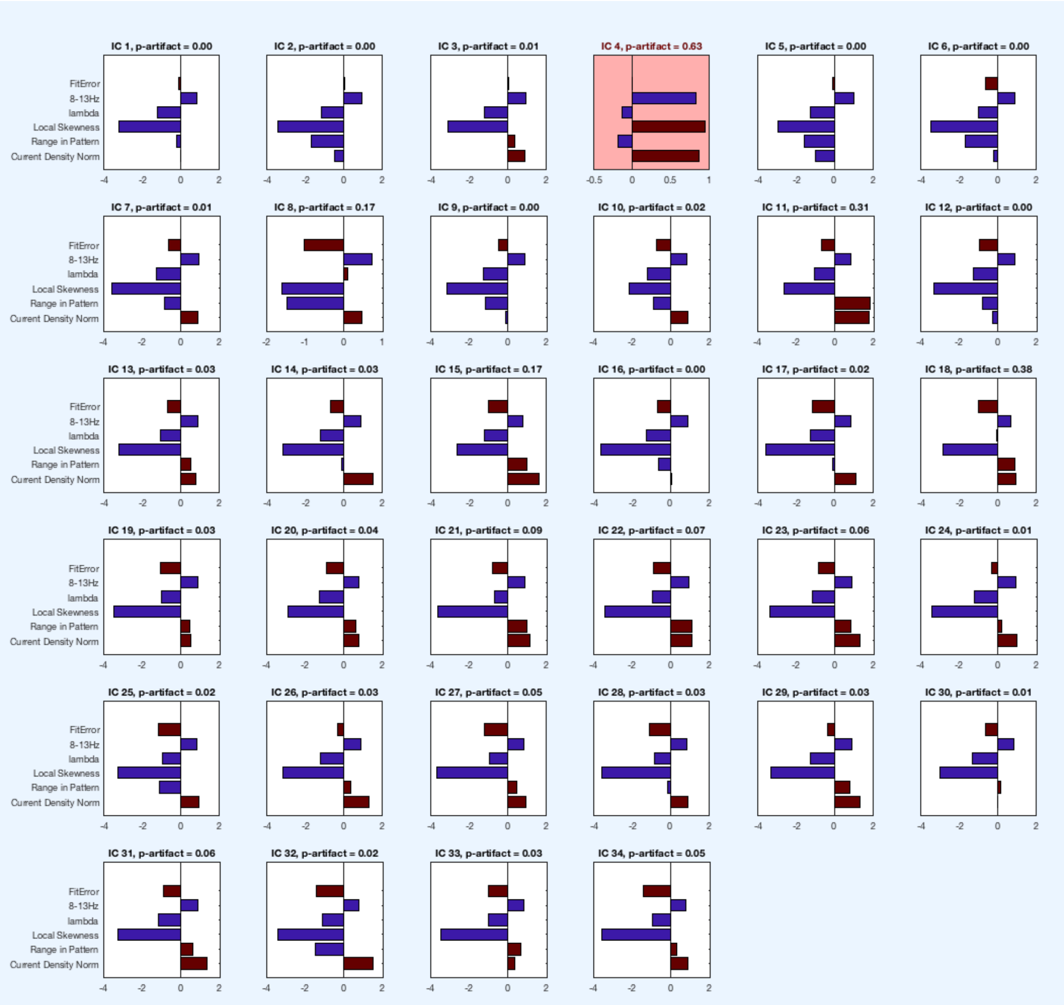

Post-processed power spectrum:

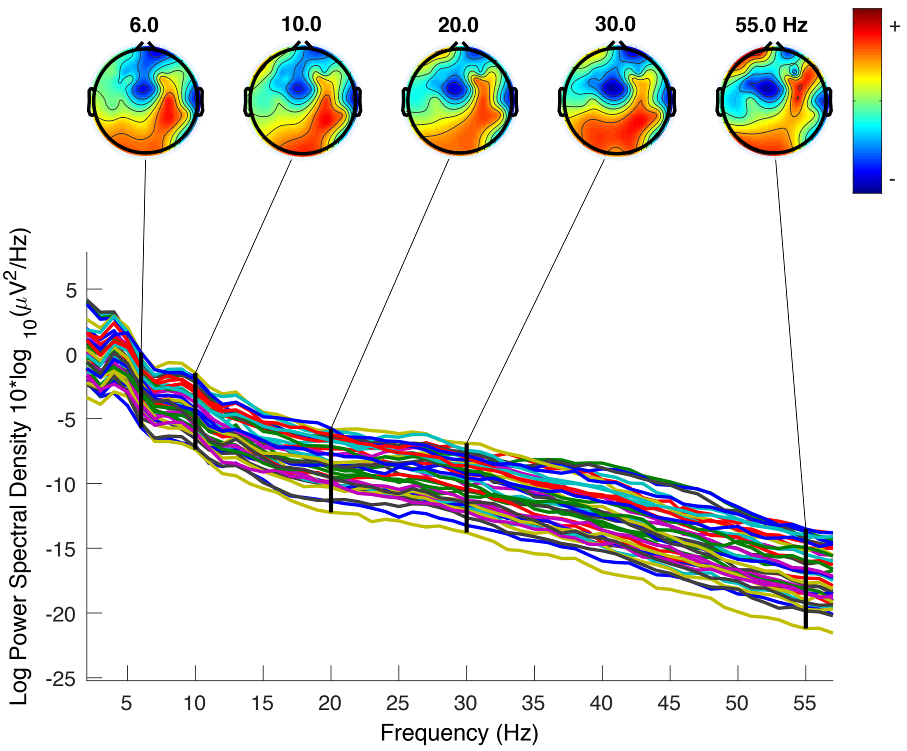

baselineEEG07 HAPPE visualizations:

W-ICA visual:

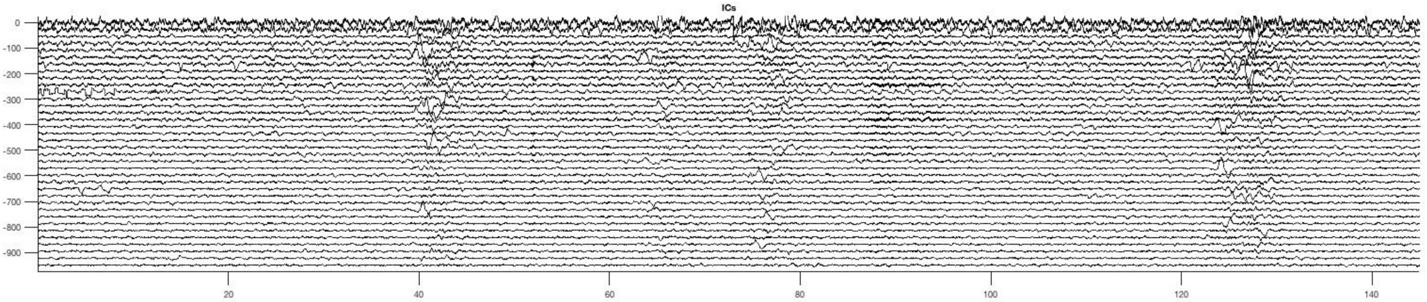

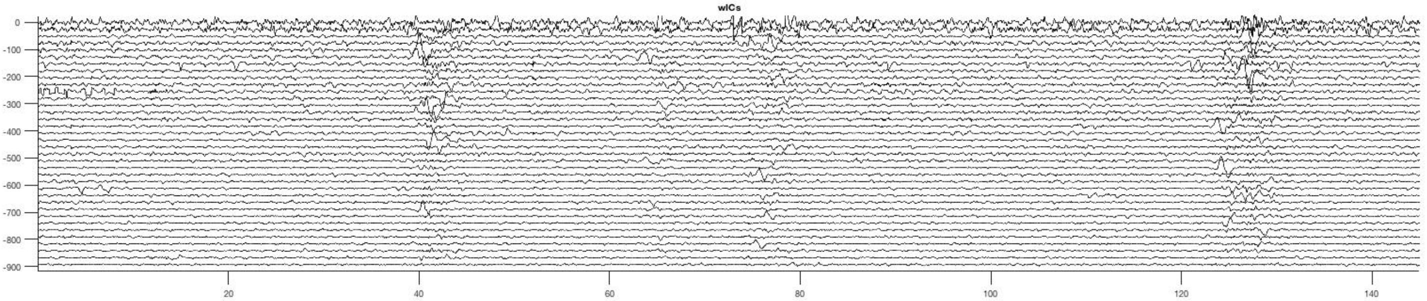

MARA visuals:

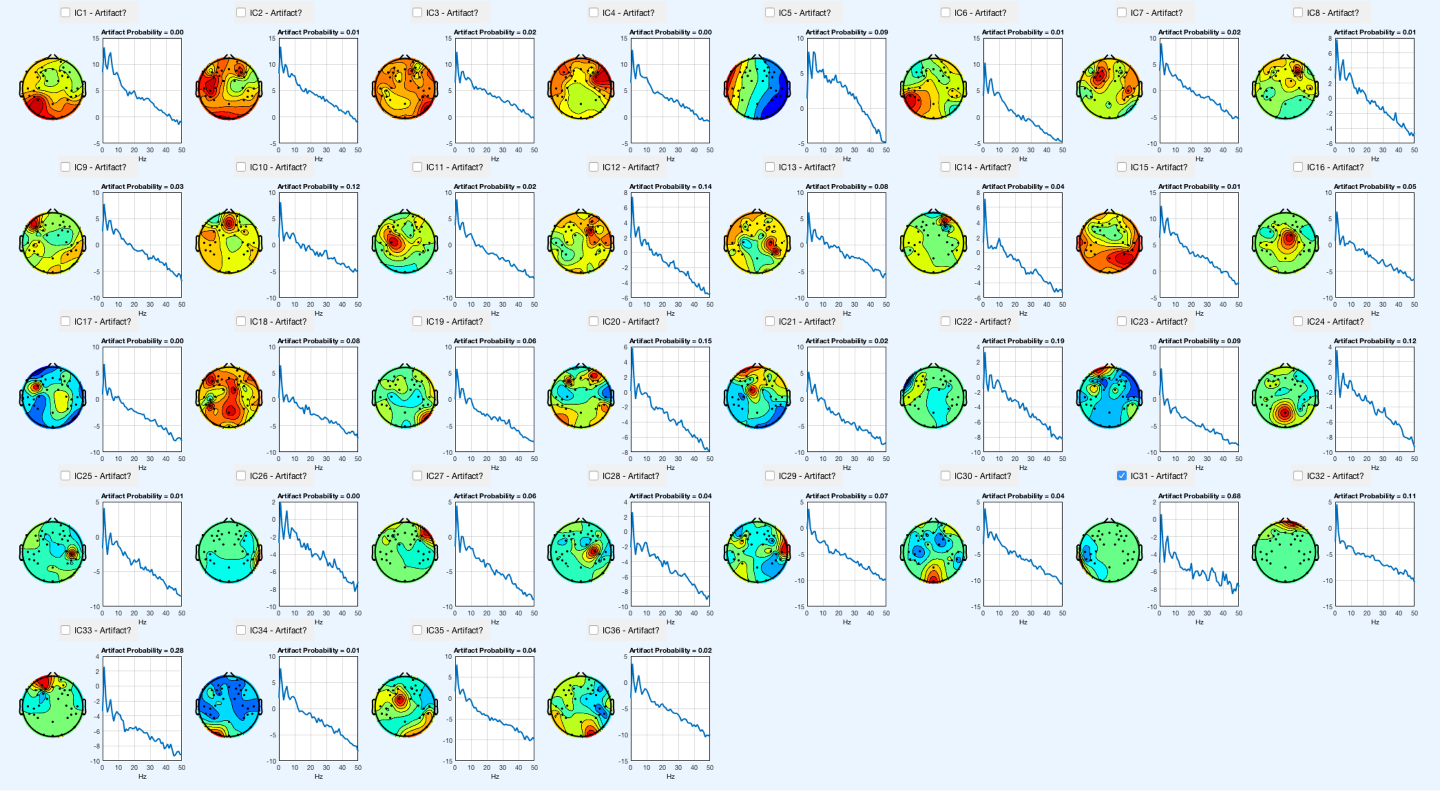

baselineEEG07 HAPPE visualizations continued

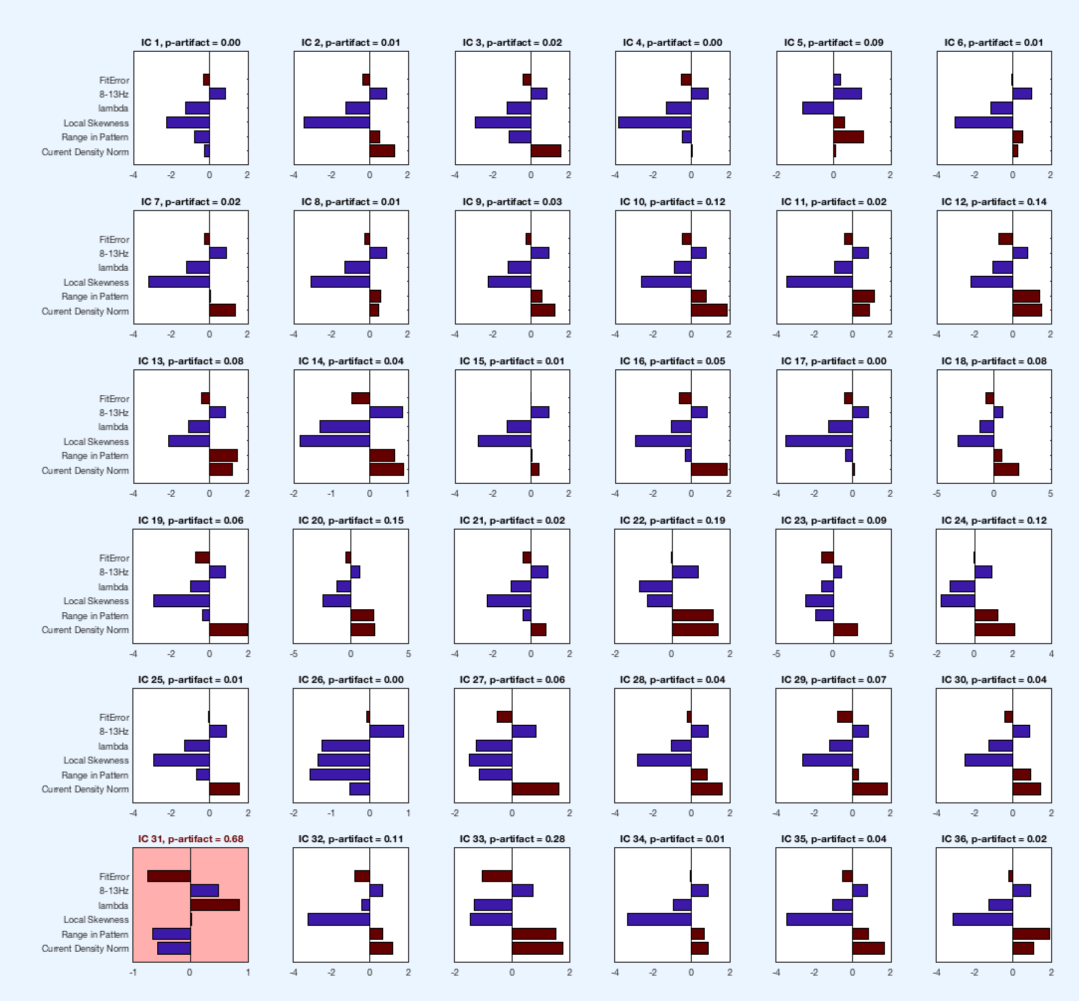

Post-processed power spectrum:

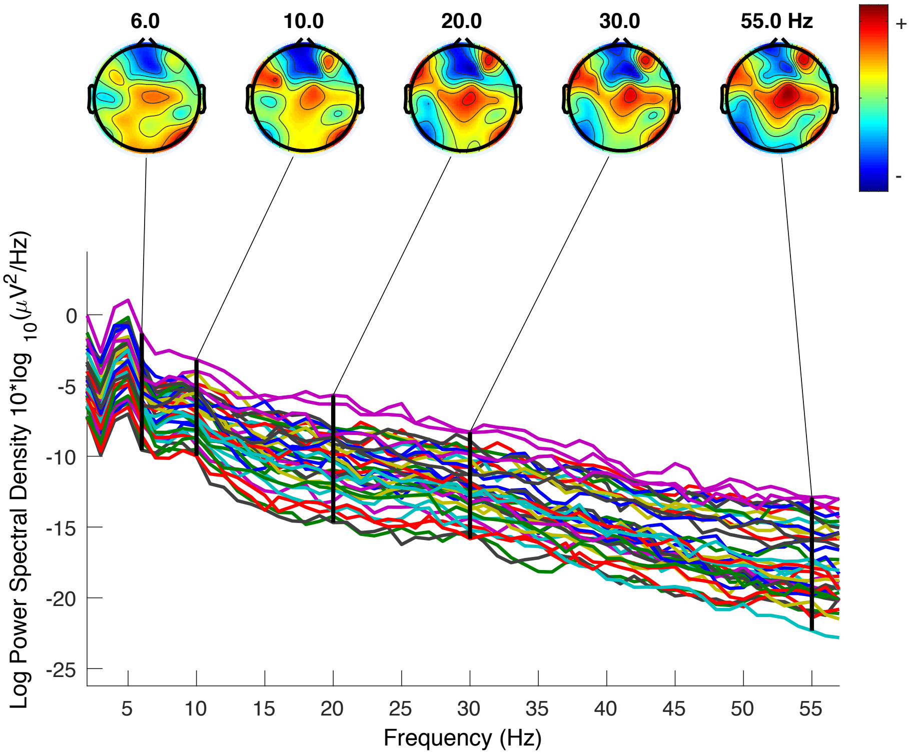

baselineEEG08 HAPPE visualizations:

W-ICA visual:

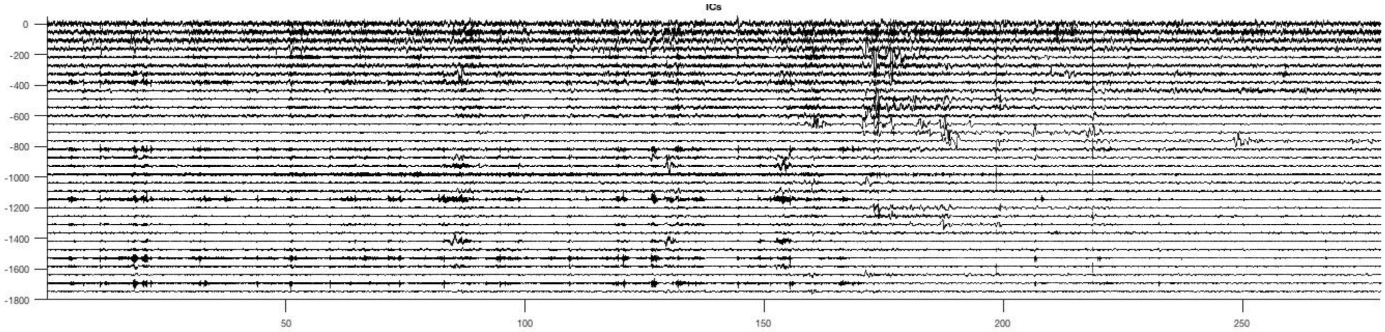

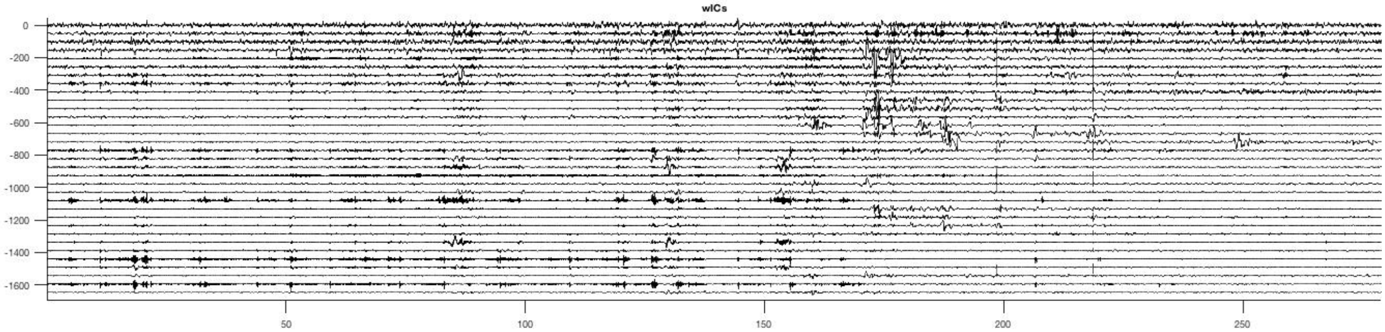

MARA visuals:

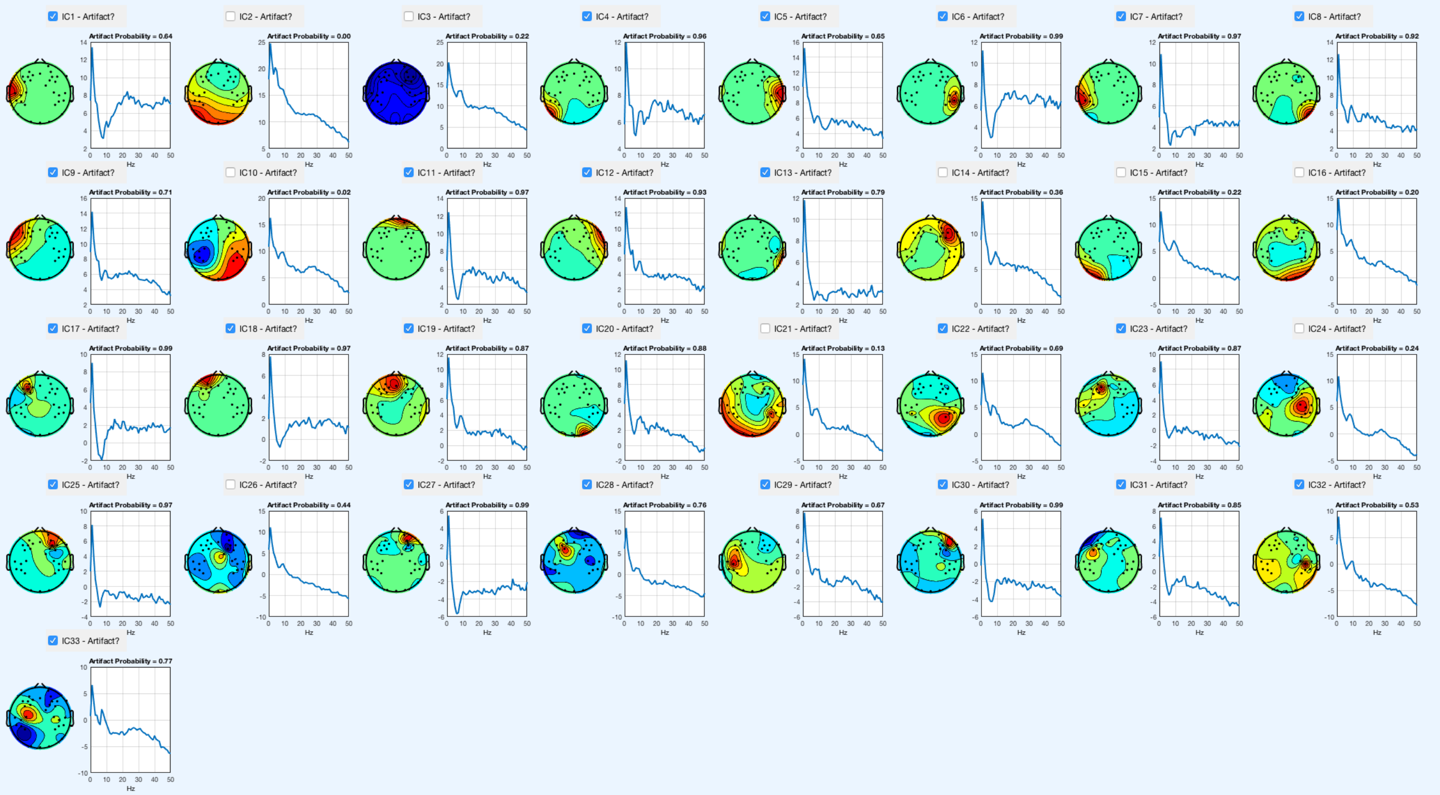

baselineEEG08 HAPPE visualizations continued

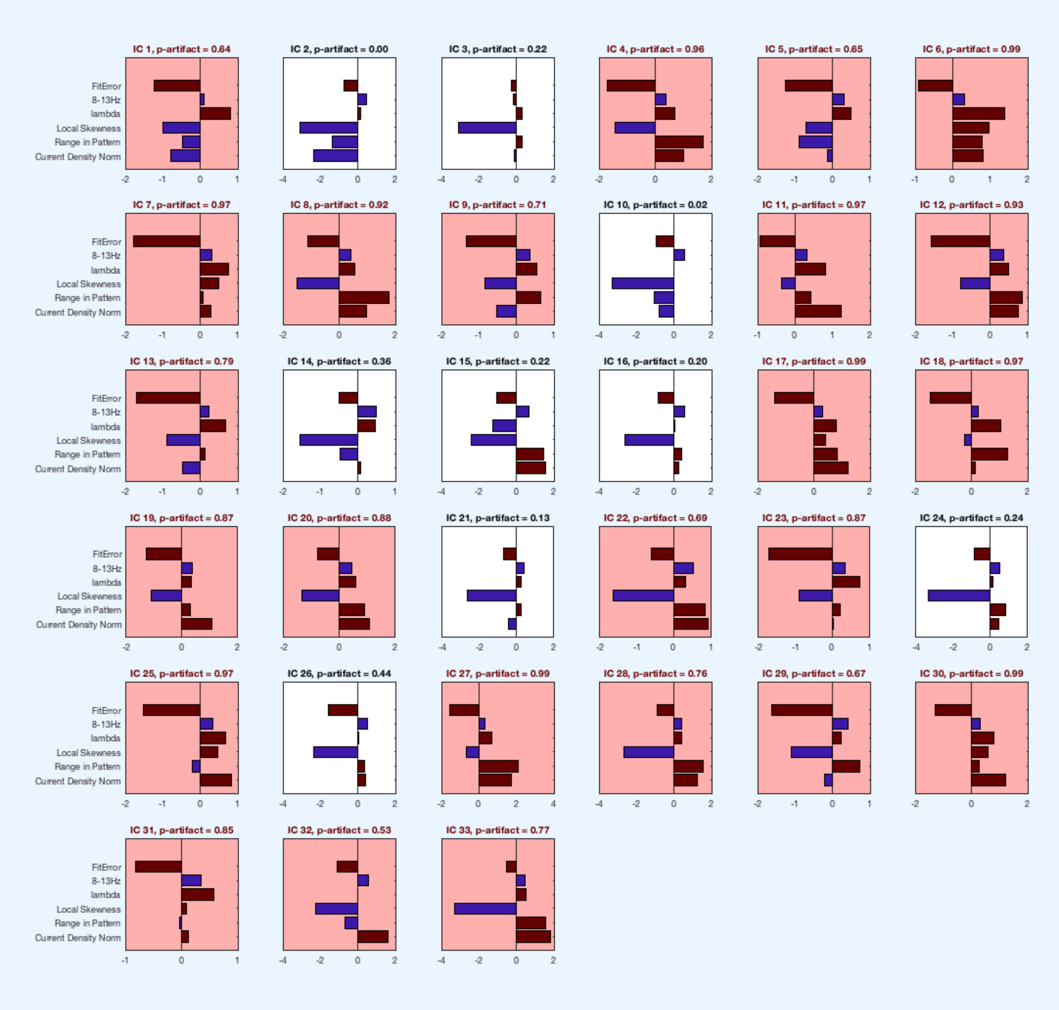

Post-processed power spectrum:

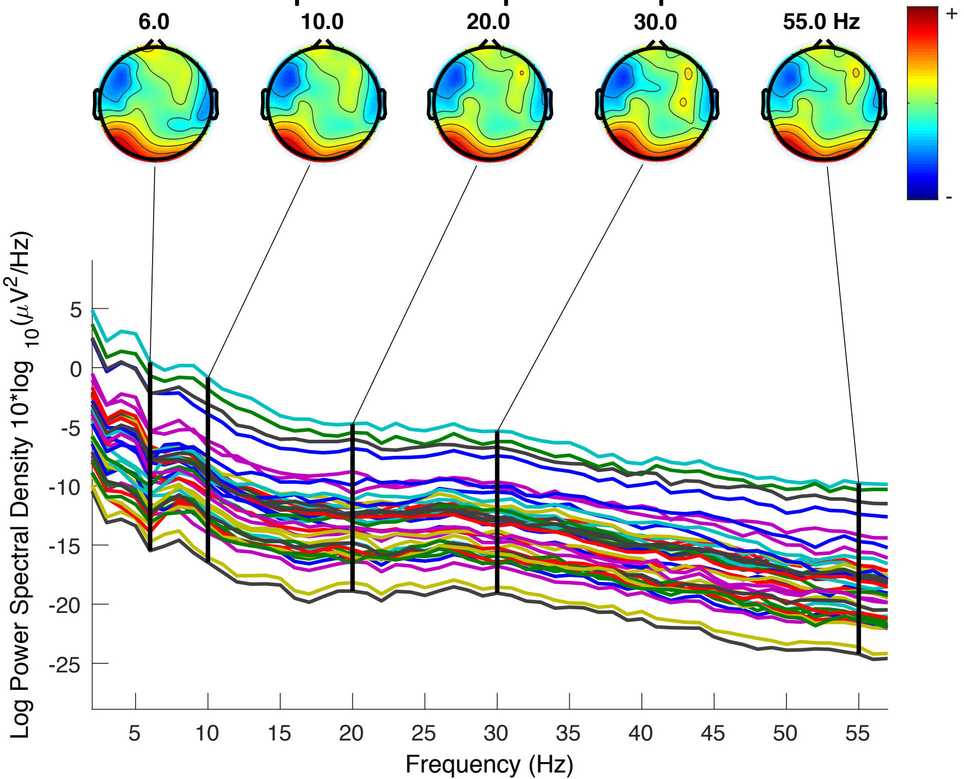

baselineEEG09 HAPPE visualizations:

W-ICA visual:

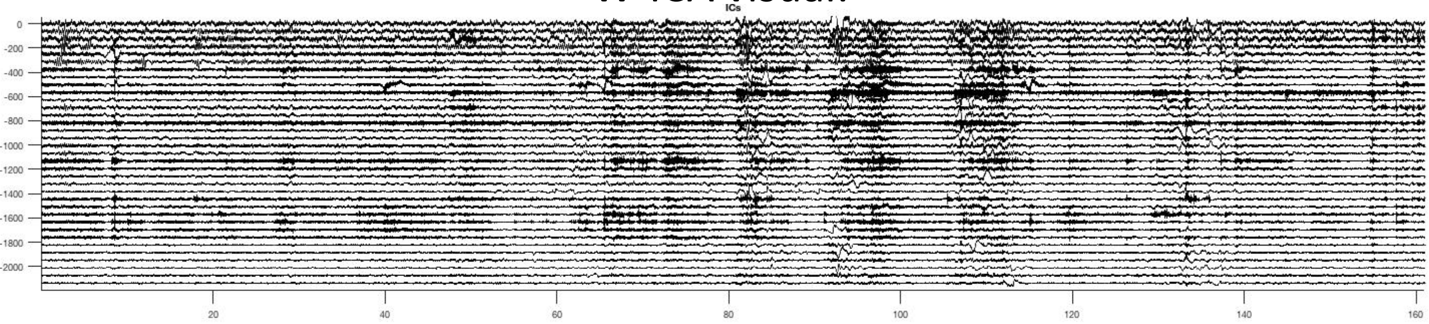

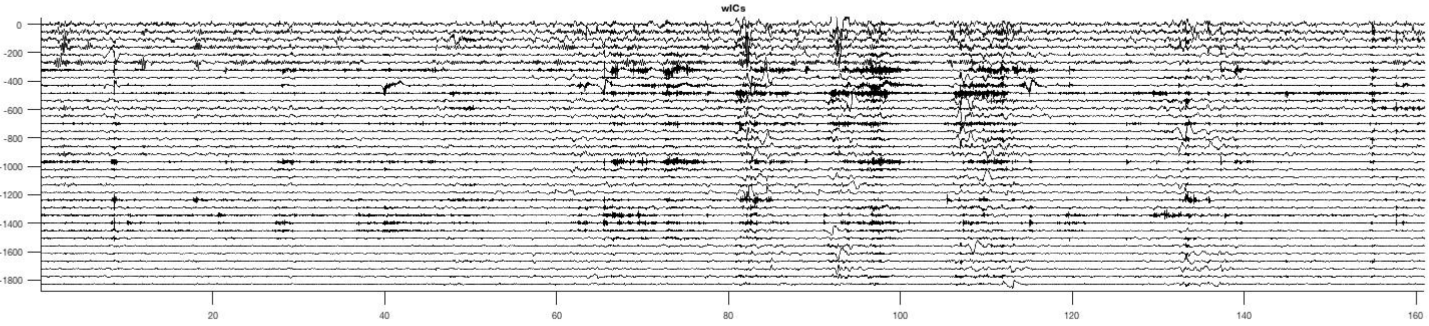

MARA visuals:

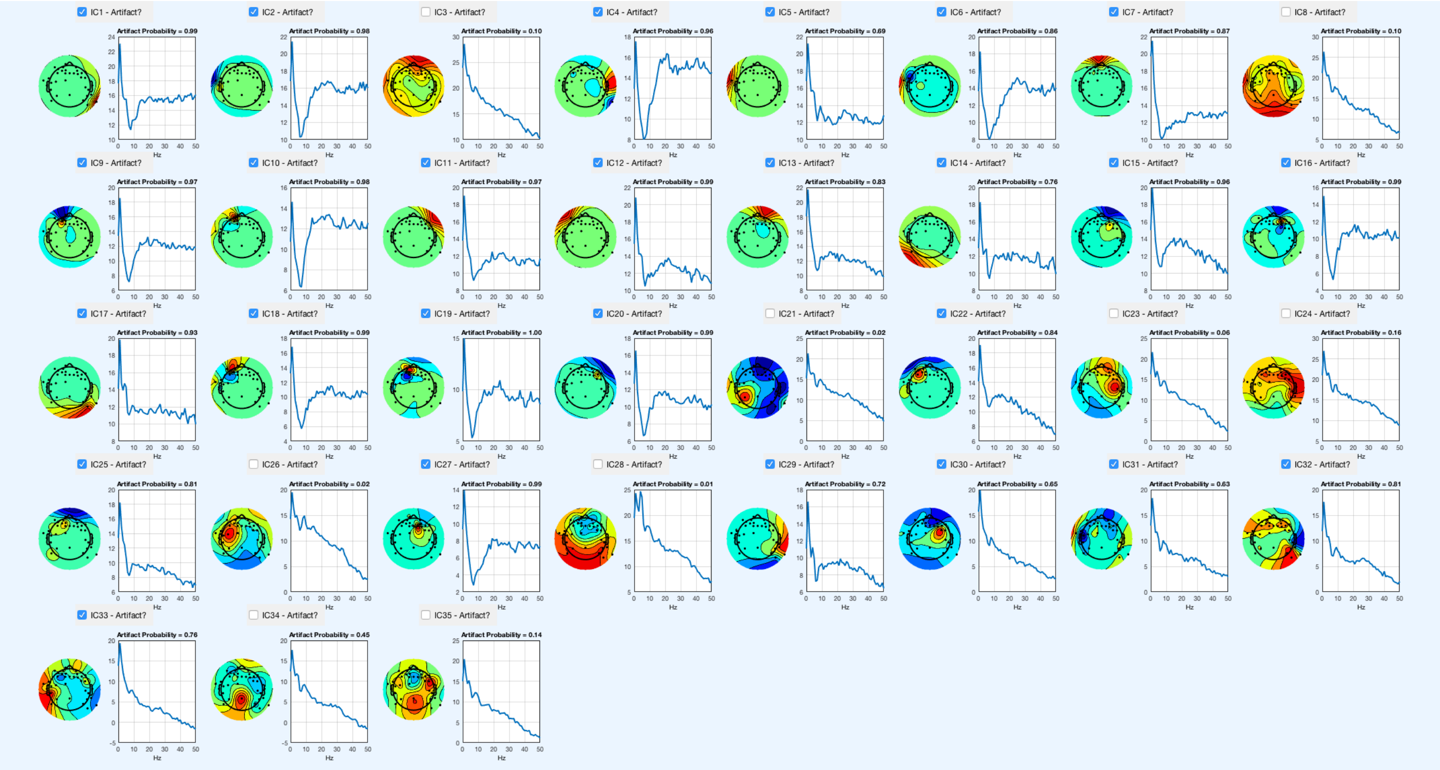

baselineEEG09 HAPPE visualizations continued

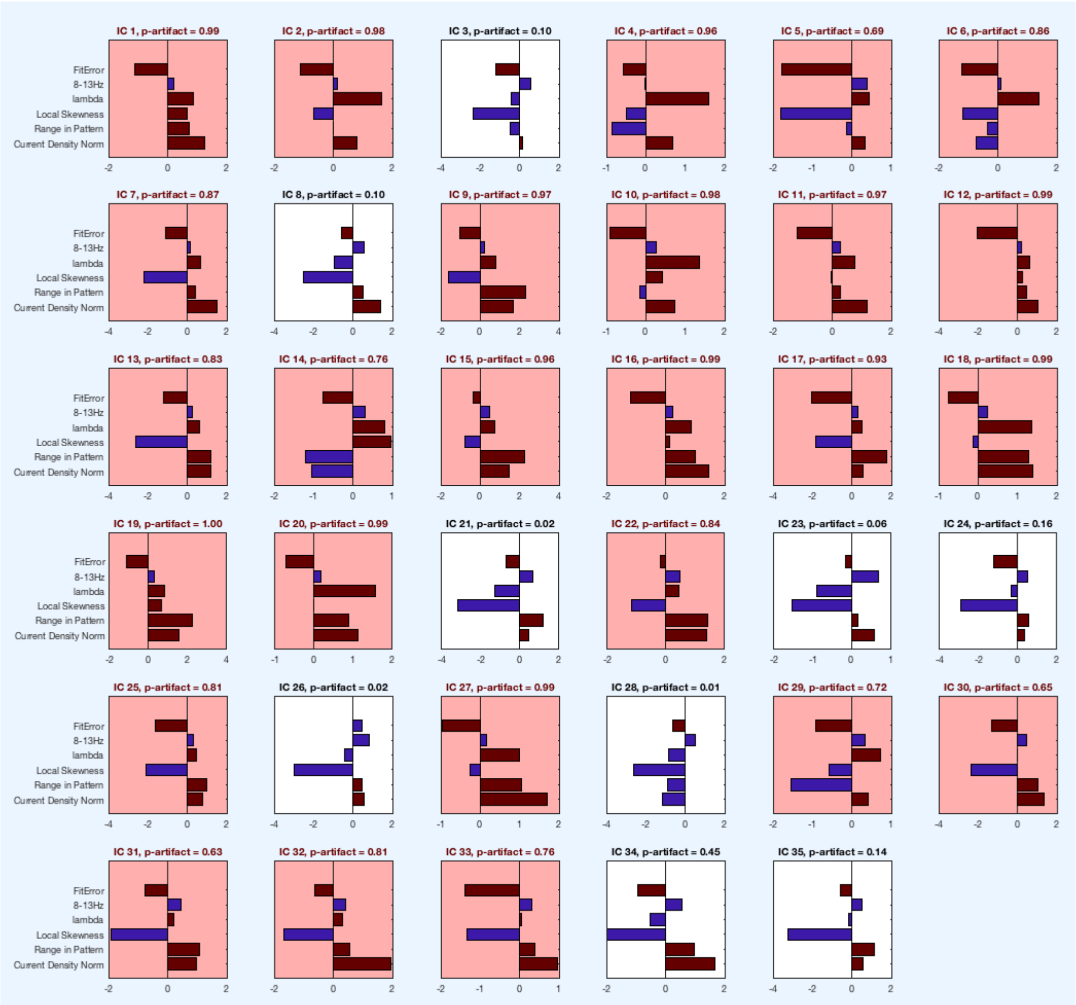

Post-processed power spectrum:

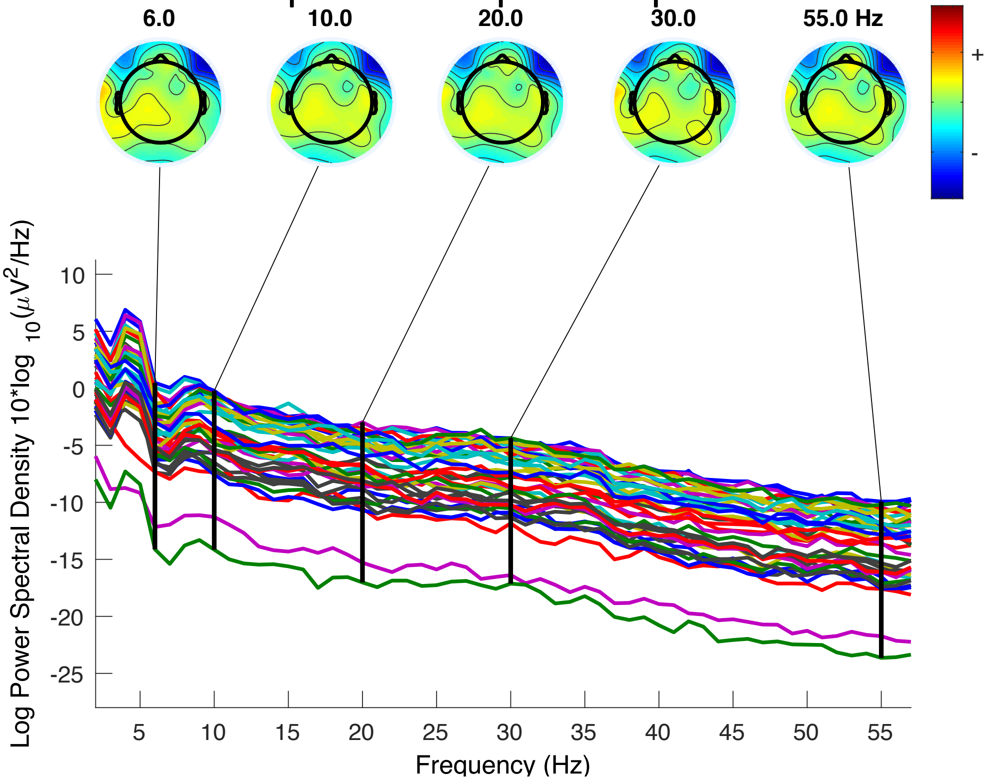

baselineEEG10 HAPPE visualizations:

W-ICA visual:

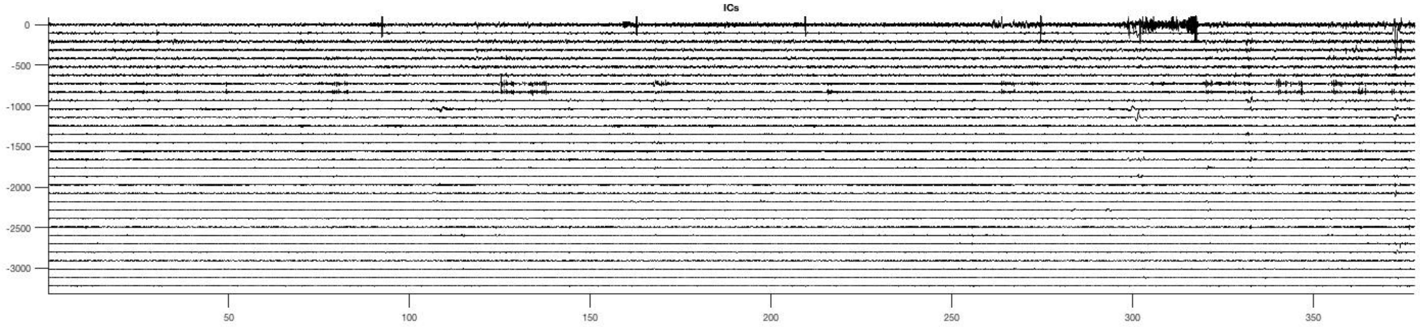

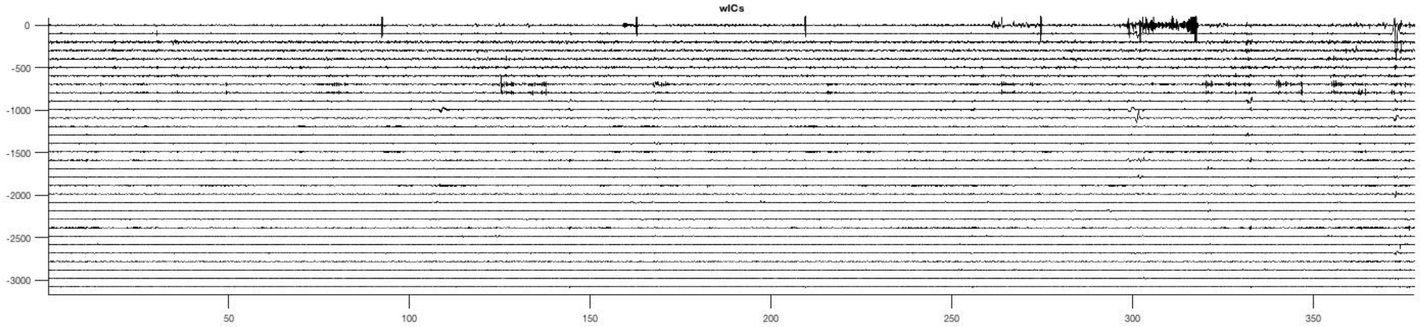

MARA visuals:

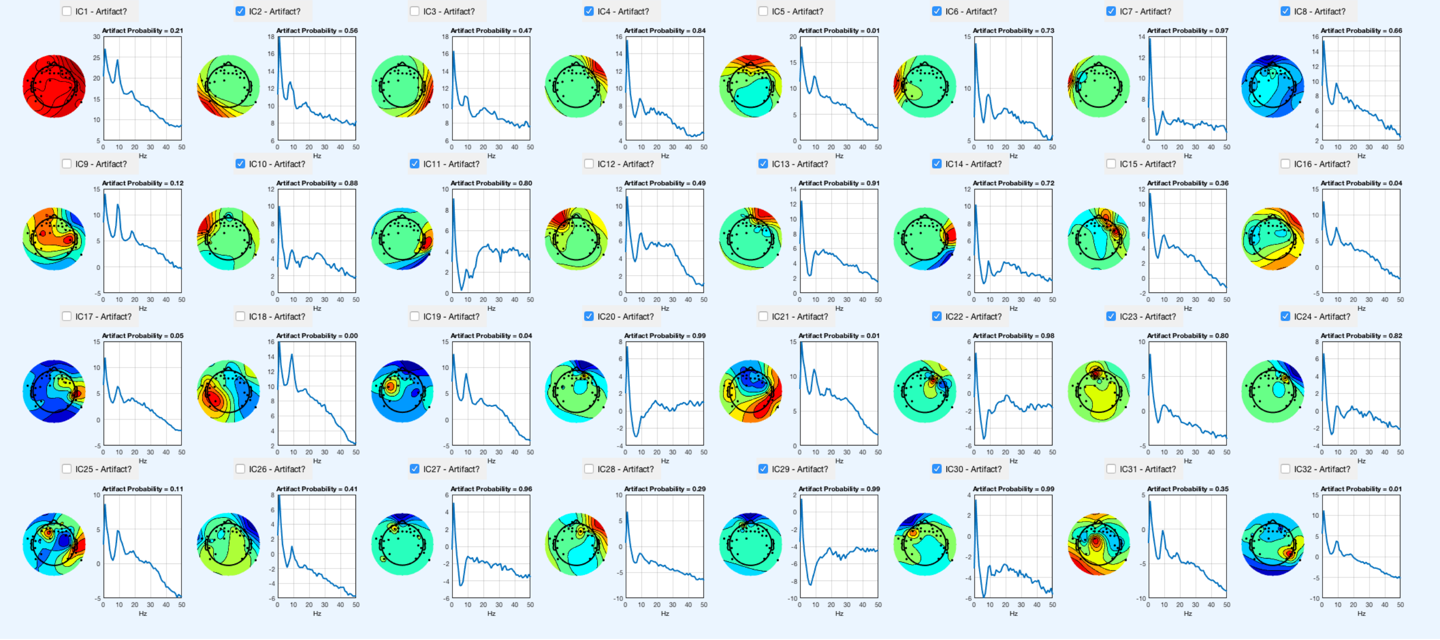

baselineEEG10 HAPPE visualizations continued

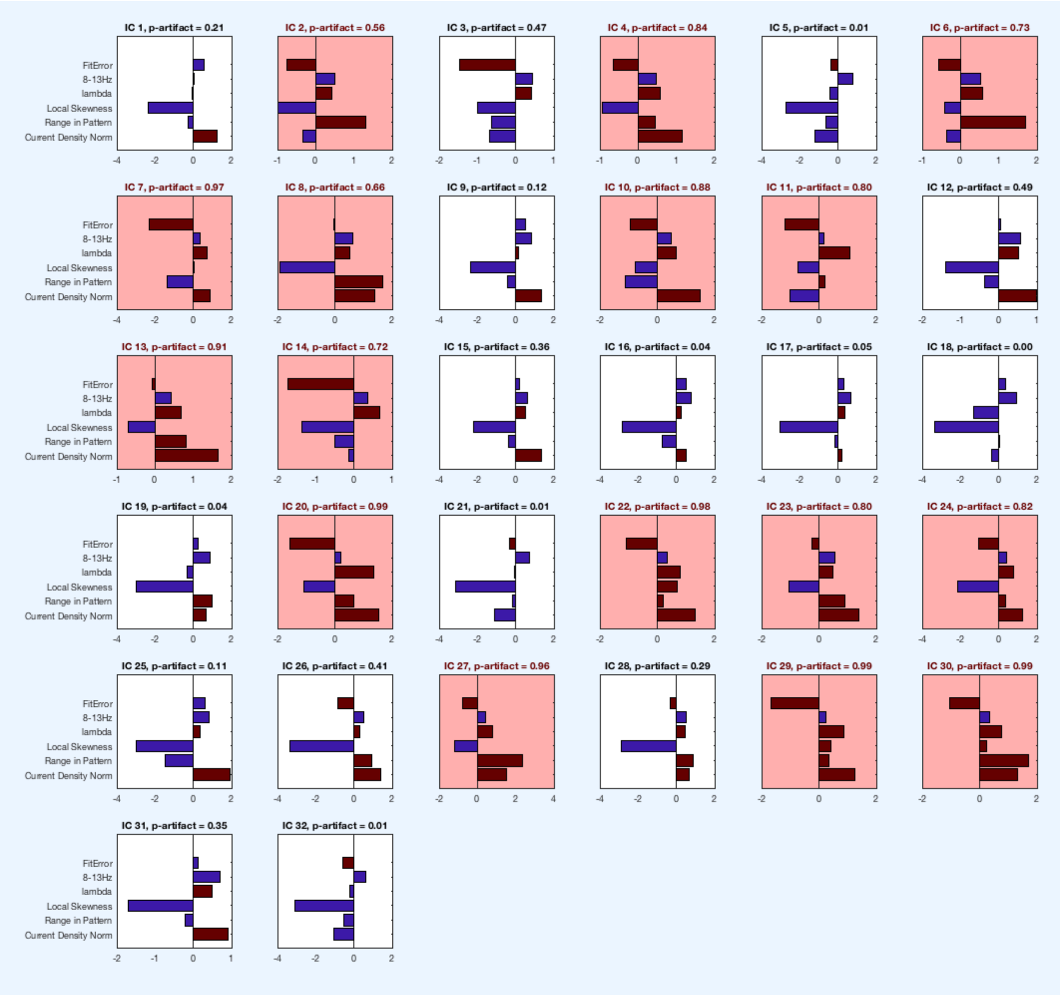

Post-processed power spectrum:

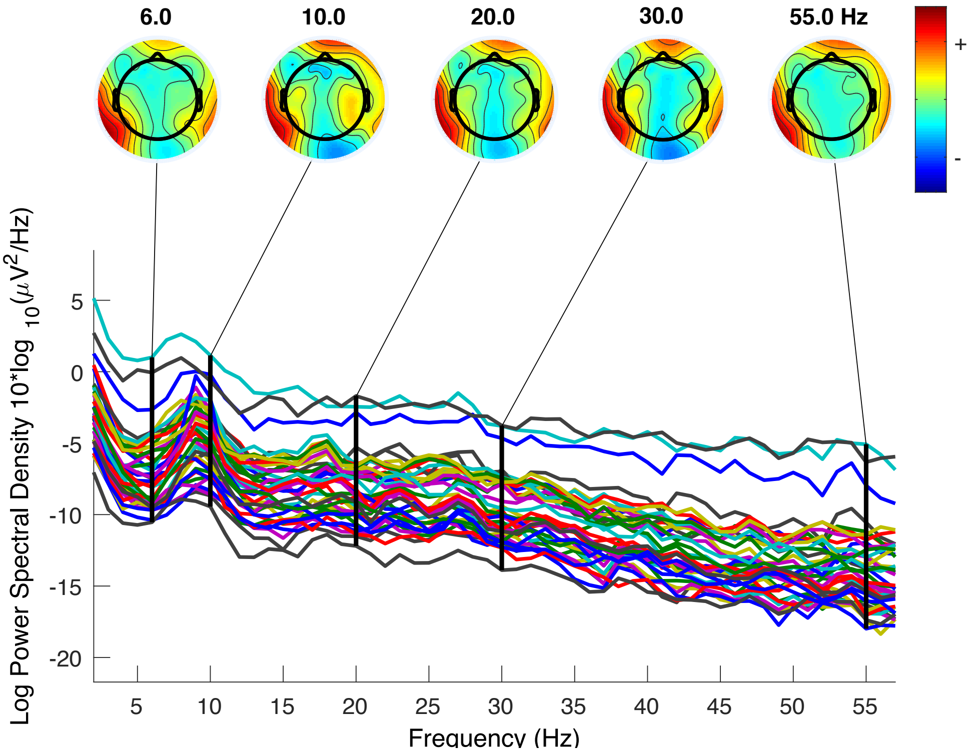

baselineEEG11 HAPPE visualizations:

W-ICA visual:

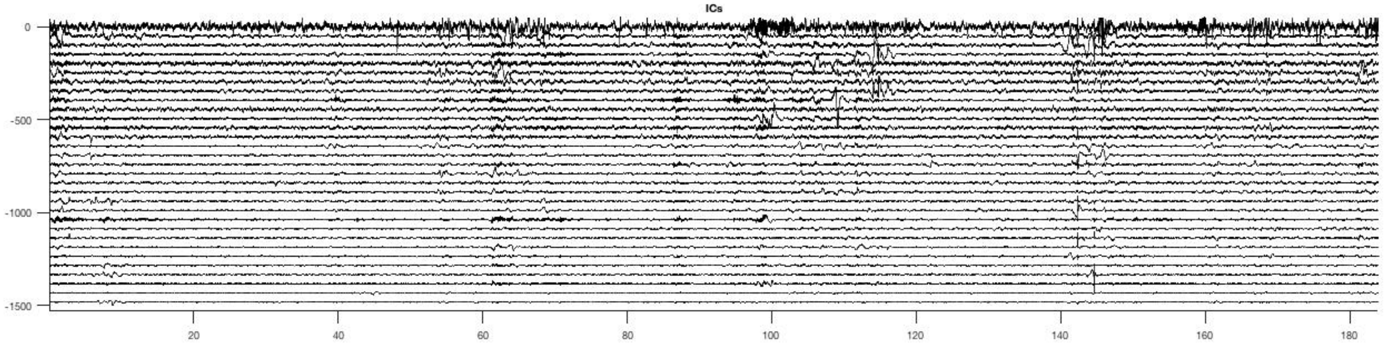

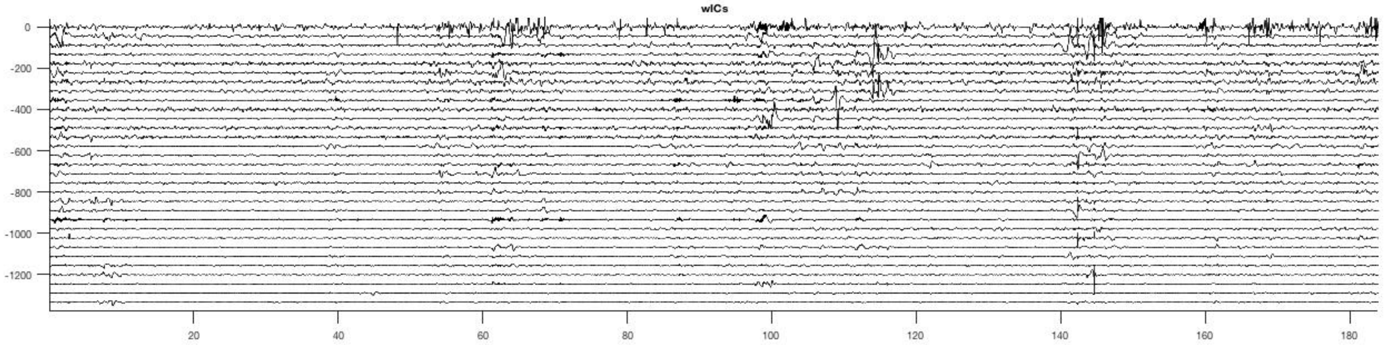

MARA visuals:

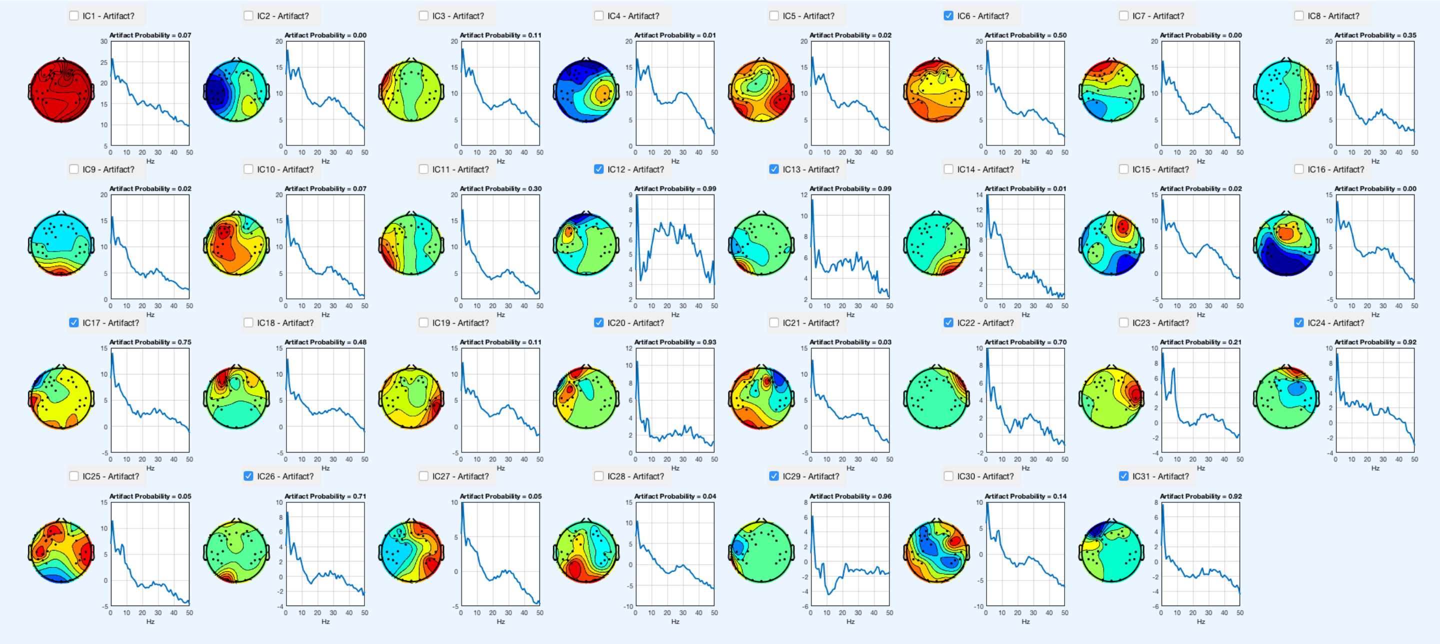

baselineEEG11 HAPPE visualizations continued

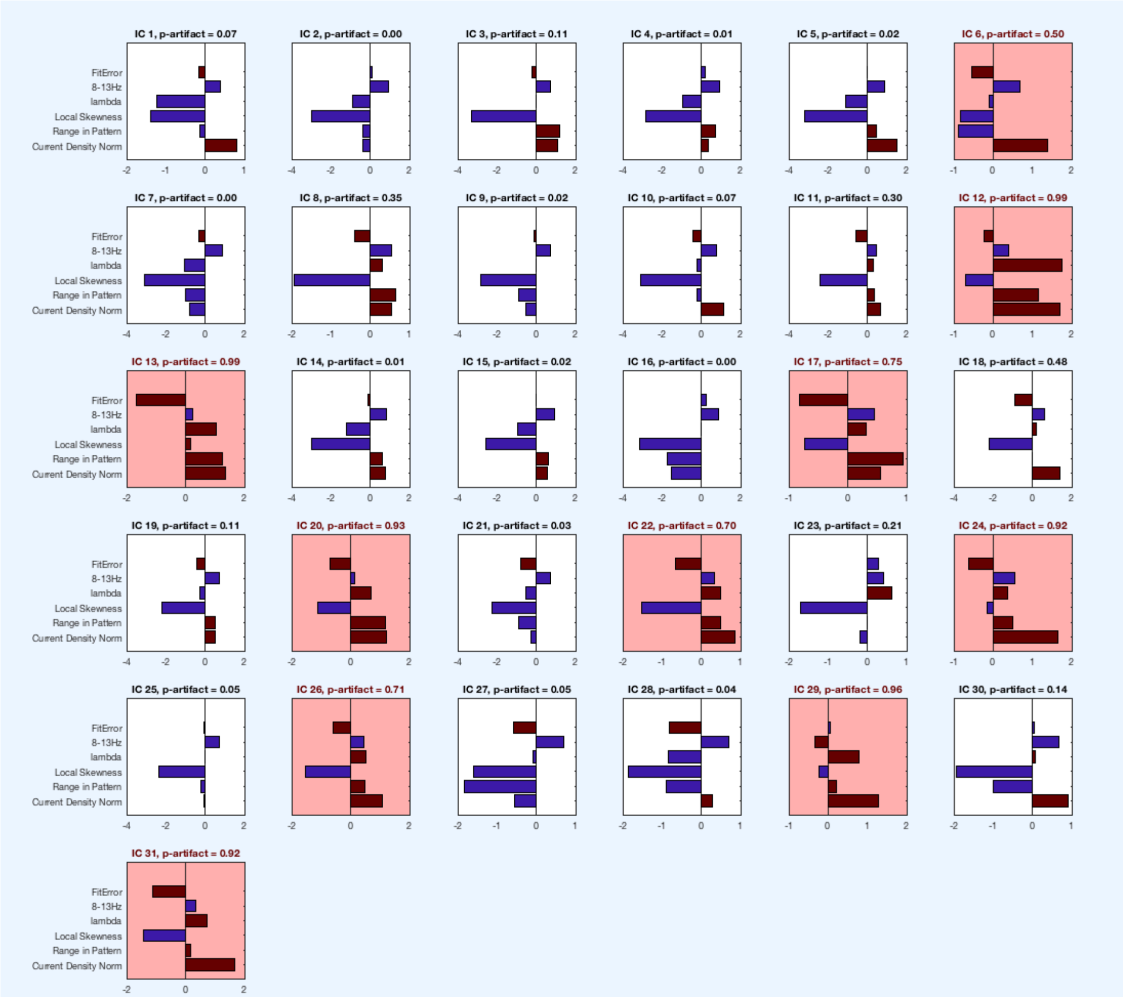

Post-processed power spectrum:

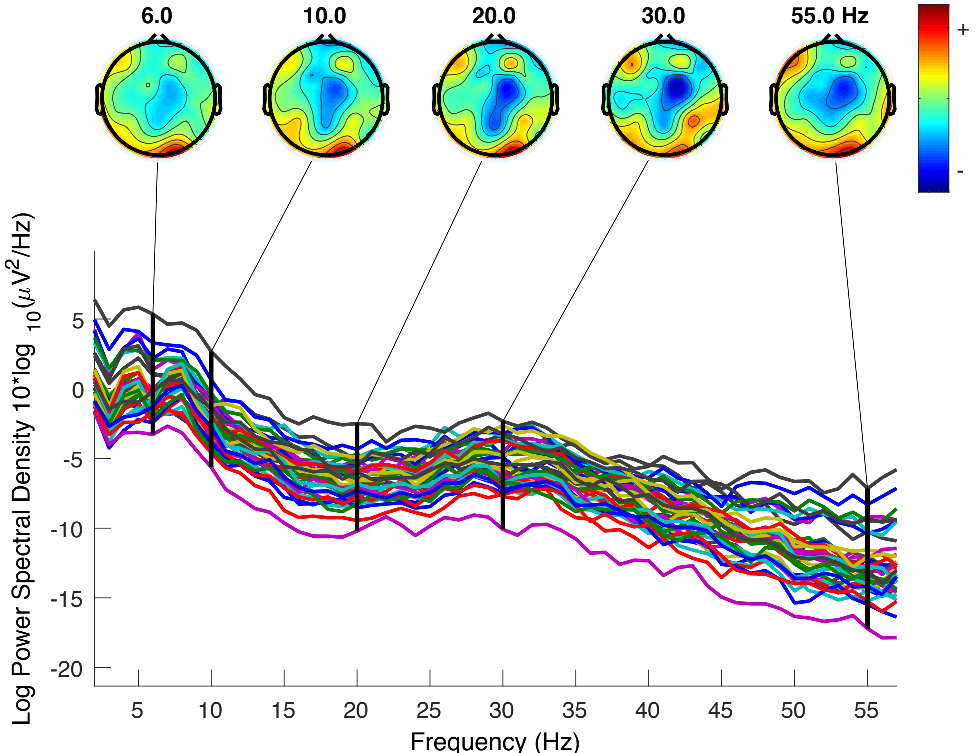

baselineEEG12 HAPPE visualizations:

W-ICA visual:

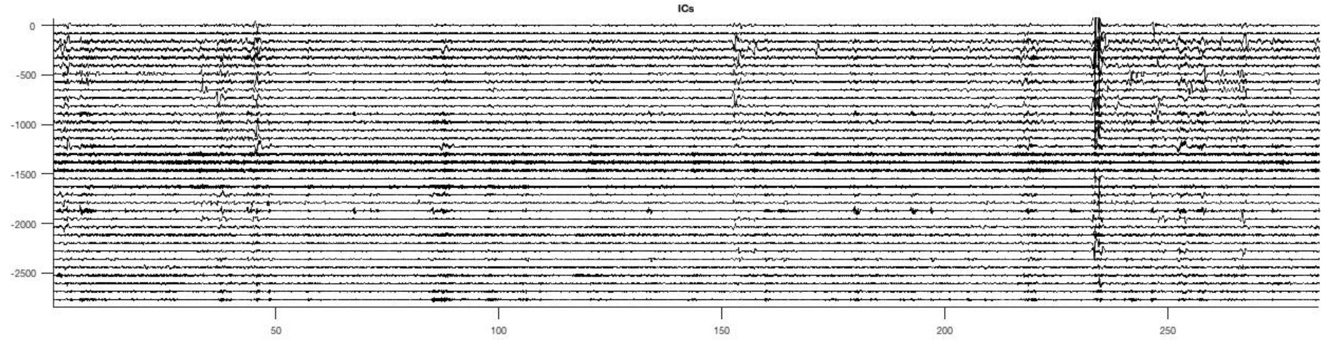

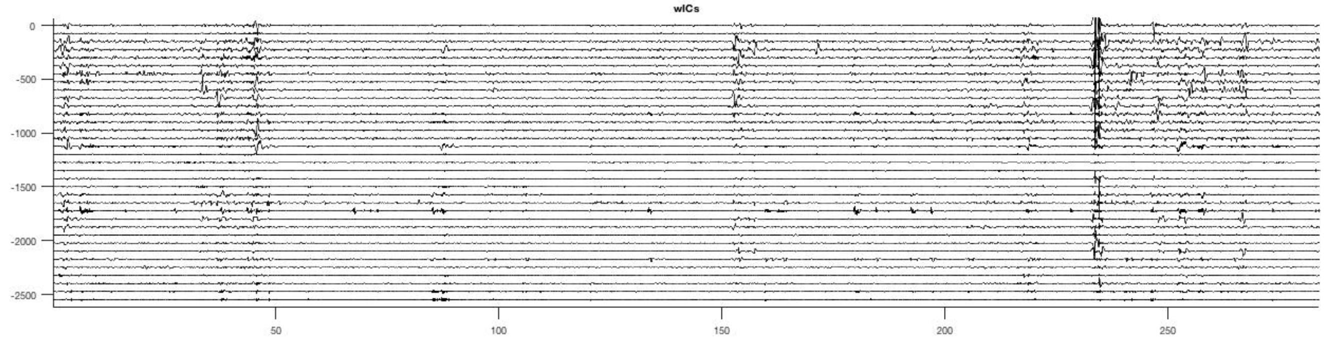

MARA visuals:

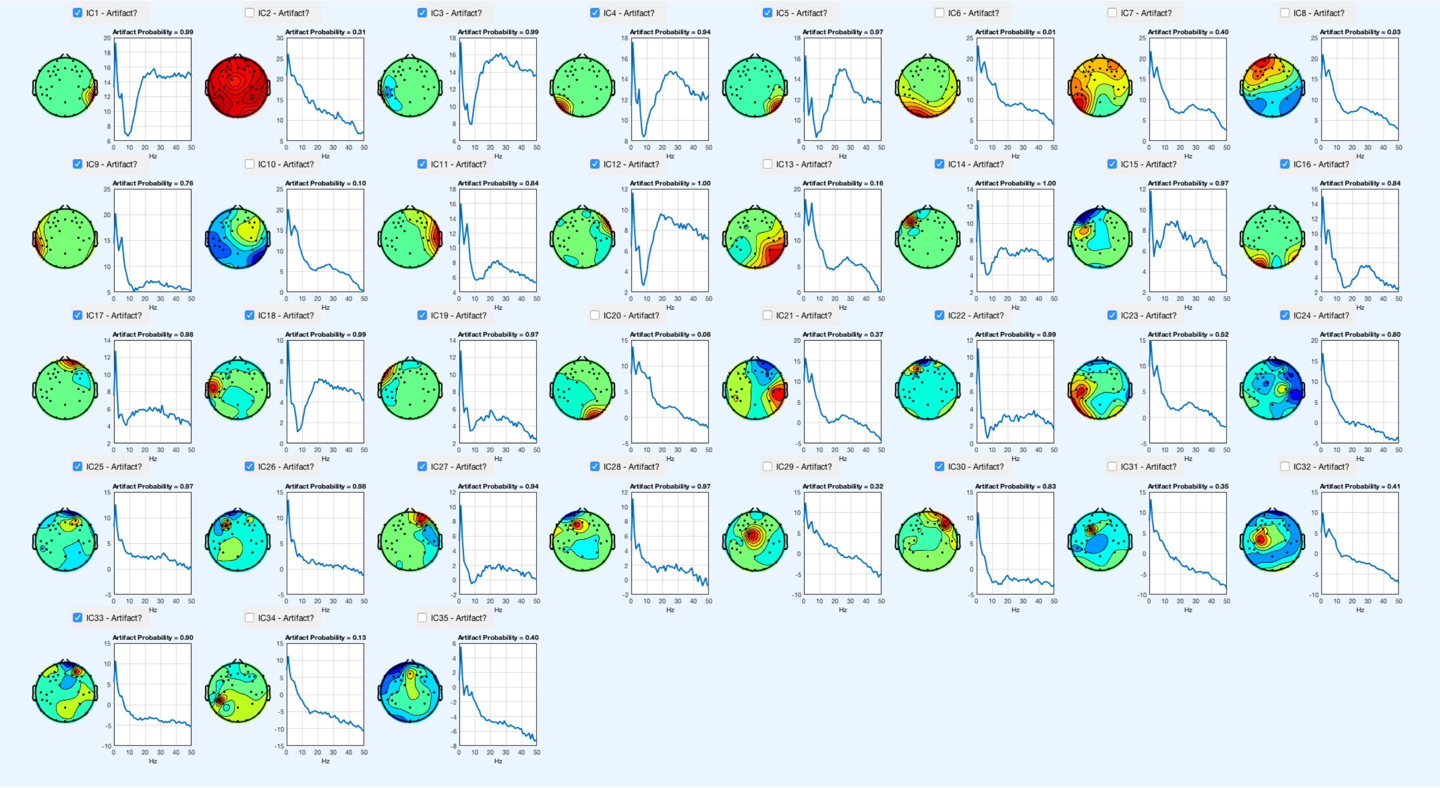

baselineEEG12 HAPPE visualizations continued

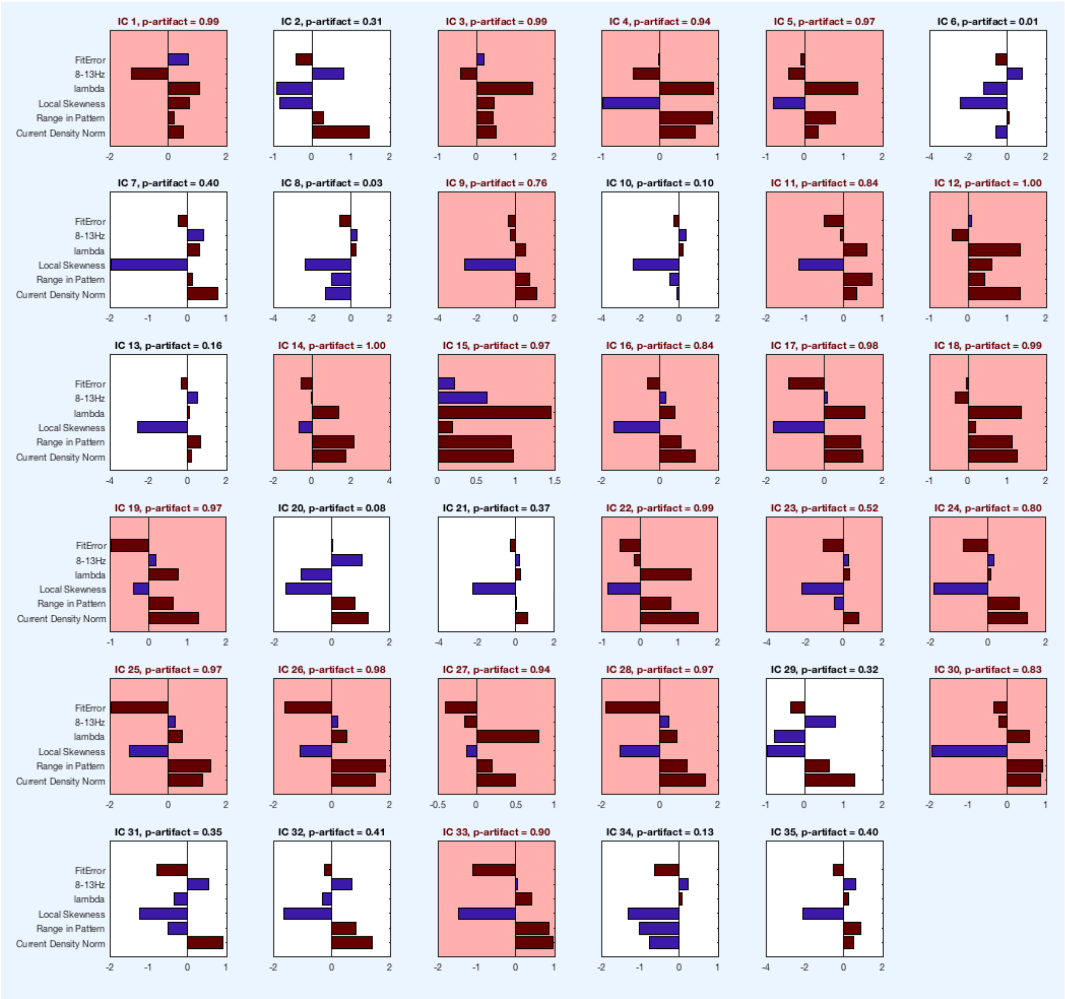

Post-processed power spectrum:

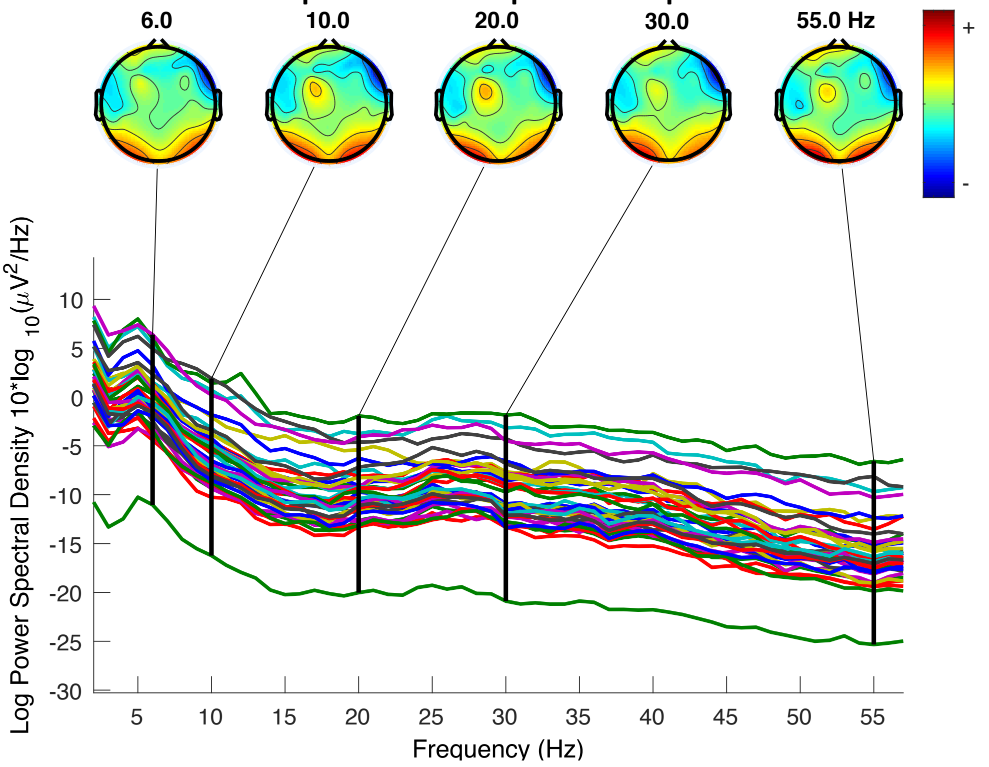

Supplement: Supplementary file 2 [file Image1.PDF]
